# Supplementary material for: VirClust—A Tool for Hierarchical Clustering, Core Protein Detection and Annotation of (Prokaryotic) Viruses
Source: Viruses. 2023 Apr 19;15(4):1007. doi: 10.3390/v15041007 (PMC10143988; doi:10.3390/v15041007)
Supplement: Supplementary file 1 [file SI_File7_dsDNA_DB_PSC_trees.pdf › SI_File7_dsDNA_DB_PSC_trees.pdf]

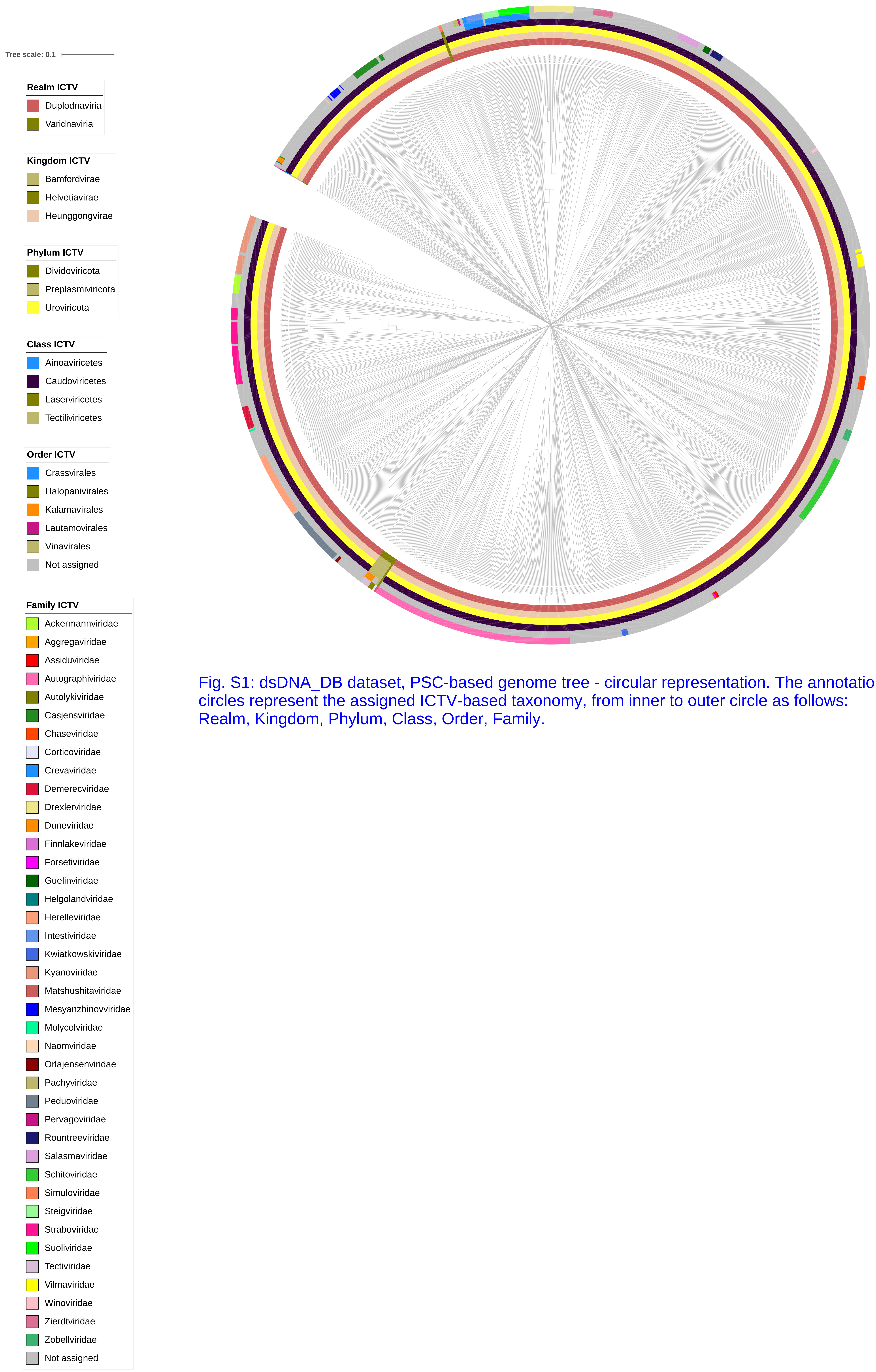

Fig. S1: dsDNA_DB dataset, PSC-based genome tree - circular representation. The annotation circles represent the assigned ICTV-based taxonomy, from inner to outer circle as follows: Realm, Kingdom, Phylum, Class, Order, Family.

Fig. S2: dsDNA_DB dataset, PSC-based genome tree - rectangular representation.

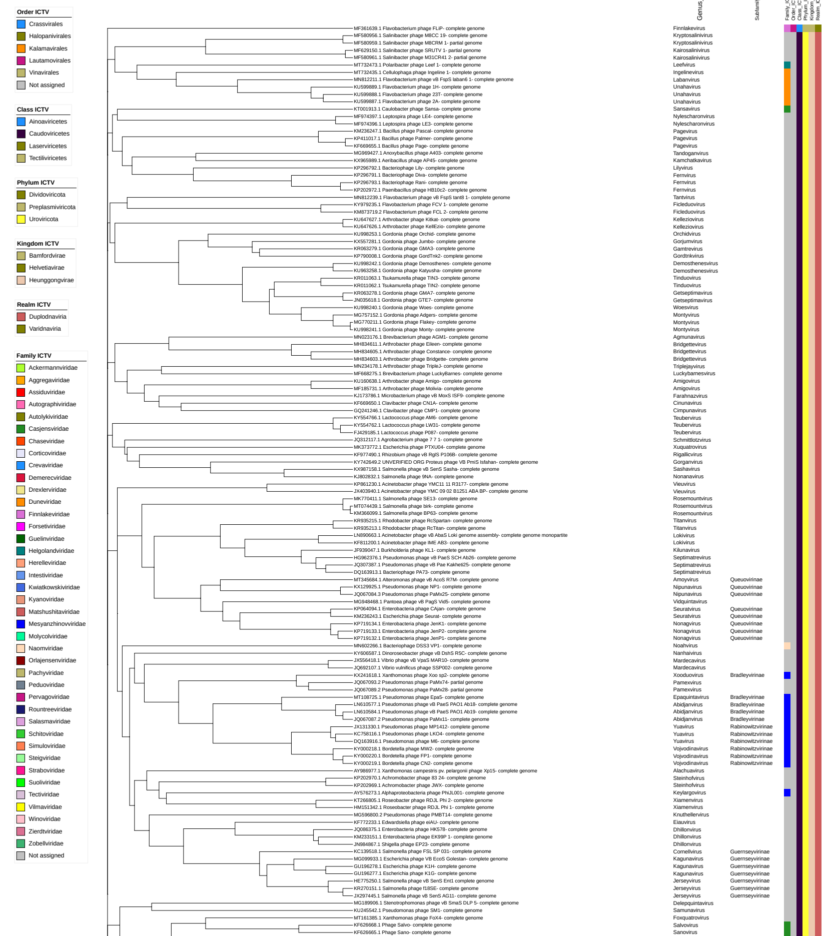

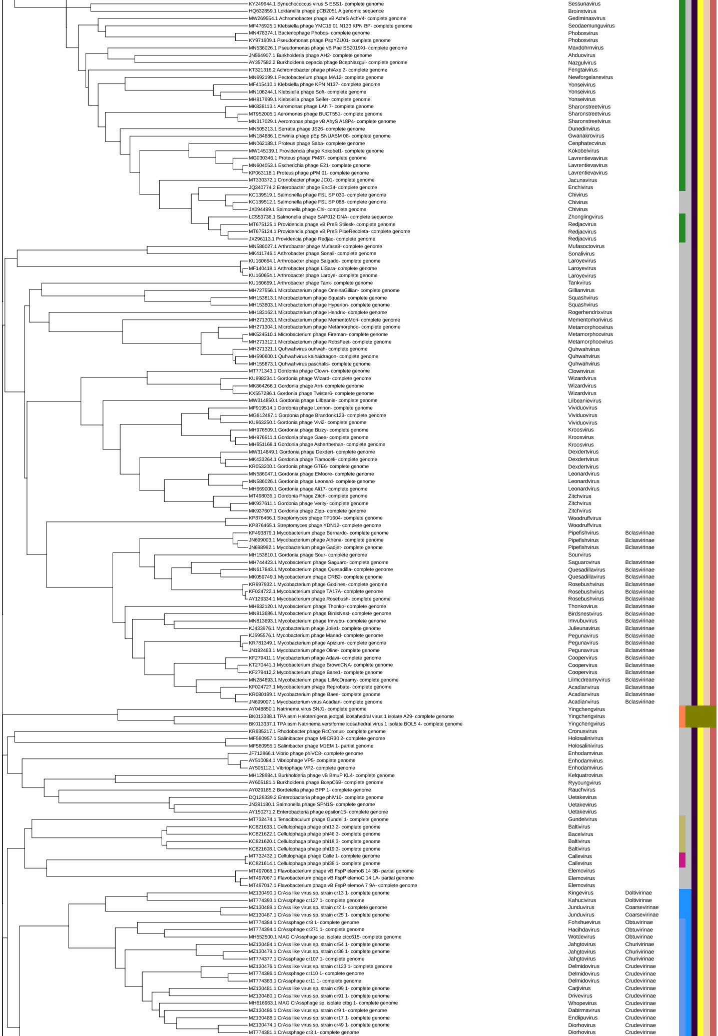
KY249644.1 Synechococcus virus S ESS1- complete genome Sessunavirus
HQ632859.1 Loktanella phage pCB2051 A genomic sequence Broinstvirus
MW269554.1 Achromobacter phage vB AchrS AchV4- complete genome Gediminasvirus
MF476925.1 Klebsiella phage YMC16 01 N133 KPN BP- complete genome Seodaemunguvirus
MN478374.1 Bacteriophage Phobos- complete genome Phobosvirus
KY971609.1 Pseudomonas phage PspYZU01- complete genome Phobosvirus
MN536026.1 Pseudomonas phage vB Pae SS2019XI- complete genome Maxdohrnvirus
JN564907.1 Burkholderia phage AH2- complete genome Ahduovirus
AY357582.2 Burkholderia cepacia phage BcepNazgul- complete genome Nazgulvirus
KT321316.2 Achromobacter phage phiAxp 2- complete genome Fengtaivirus
MN692199.1 Pectobacterium phage MA12- complete genome Newforgelanevirus
MF415410.1 Klebsiella phage KPN N137- complete genome Yonseivirus
MN106244.1 Klebsiella phage Soft- complete genome Yonseivirus
MH817999.1 Klebsiella phage Seifer- complete genome Yonseivirus
MK838113.1 Aeromonas phage LAh 7- complete genome Sharonstreetvirus
MT952005.1 Aeromonas phage BUCT551- complete genome Sharonstreetvirus
MN317029.1 Aeromonas phage vB AhyS A18P4- complete genome Sharonstreetvirus
MN505213.1 Serratia phage JS26- complete genome Dunedinvirus
MN184886.1 Erwinia phage pEp SNUABM 08- complete genome Gwanakrovirus
MN062188.1 Proteus phage Saba- complete genome Cenphatecvirus
MW145139.1 Providencia phage Kokobel1- complete genome Kokobelvirus
MG030346.1 Proteus phage PM87- complete genome Lavrentievavirus
MN604053.1 Escherichia phage E21- complete genome Lavrentievavirus
KP063118.1 Proteus phage pPM 01- complete genome Lavrentievavirus
MT330372.1 Cronobacter phage JC01- complete genome Jacunavirus
JQ340774.2 Enterobacter phage Enc34- complete genome Enchivirus
KC139519.1 Salmonella phage FSL SP 030- complete genome Chivirus
KC139512.1 Salmonella phage FSL SP 088- complete genome Chivirus
JX094499.1 Salmonella phage Chi- complete genome Chivirus
LC553736.1 Salmonella phage SAP012 DNA- complete sequence Zhonglingvirus
MT675125.1 Providencia phage vB PreS Stilesk- complete genome Redjacvirus
MT675124.1 Providencia phage vB PreS PibeRecoleta- complete genome Redjacvirus
JX296113.1 Providencia phage Redjac- complete genome Redjacvirus
MN586027.1 Arthrobacter phage Mufasa8- complete genome Mufasoctovirus
MK411746.1 Arthrobacter phage Sonali- complete genome Sonalivirus
KU160664.1 Arthrobacter phage Salgado- complete genome Laroyevirus
MF140418.1 Arthrobacter phage LiSara- complete genome Laroyevirus
KU160654.1 Arthrobacter phage Laroye- complete genome Laroyevirus
KU160669.1 Arthrobacter phage Tank- complete genome Tankvirus
MH727556.1 Microbacterium phage OneinaGillian- complete genome Gillianvirus
MH153813.1 Microbacterium phage Squash- complete genome Squashvirus
MH153803.1 Microbacterium phage Hyperion- complete genome Squashvirus
MH183162.1 Microbacterium phage Hendrix- complete genome Rogerhendrixvirus
MH271303.1 Microbacterium phage MementoMori- complete genome Mementomorivirus
MH271304.1 Microbacterium phage Metamorphoo- complete genome Metamorphoovirus
MK524510.1 Microbacterium phage Fireman- complete genome Metamorphoovirus
MH271312.1 Microbacterium phage RobsFeet- complete genome Metamorphoovirus
MH271321.1 Quhwahvirus ouhwah- complete genome Quhwahvirus
MH590600.1 Quhwahvirus kaihaidragon- complete genome Quhwahvirus
MH155873.1 Quhwahvirus paschalis- complete genome Quhwahvirus
MT771343.1 Gordonia phage Clown- complete genome Clownvirus
KU998234.1 Gordonia phage Wizard- complete genome Wizardvirus
MK864266.1 Gordonia phage Arri- complete genome Wizardvirus
KX557286.1 Gordonia phage Twister6- complete genome Wizardvirus
MW314850.1 Gordonia phage Lilbeanie- complete genome Lilbeanievirus
MF919514.1 Gordonia phage Lennon- complete genome Vividuovirus
MG812487.1 Gordonia phage Brandonk123- complete genome Vividuovirus
KU963250.1 Gordonia phage Vivi2- complete genome Vividuovirus
MH976509.1 Gordonia phage Bizzy- complete genome Kroosvirus
MH976511.1 Gordonia phage Gaea- complete genome Kroosvirus
MH651168.1 Gordonia phage Ashertheman- complete genome Kroosvirus
MW314849.1 Gordonia phage Dexdert- complete genome Dexdertvirus
MK433264.1 Gordonia phage Tiamoceli- complete genome Dexdertvirus
KR053200.1 Gordonia phage GTE6- complete genome Dexdertvirus
MN586047.1 Gordonia phage EMoore- complete genome Leonardvirus
MN586026.1 Gordonia phage Leonard- complete genome Leonardvirus
MH669000.1 Gordonia phage Ali17- complete genome Leonardvirus
MT498036.1 Gordonia Phage Zitch- complete genome Zitchvirus
MK937611.1 Gordonia phage Verity- complete genome Zitchvirus
MK937607.1 Gordonia phage Zipp- complete genome Zitchvirus
KP876466.1 Streptomyces phage TP1604- complete genome Woodruffvirus
KP876465.1 Streptomyces phage YDN12- complete genome Woodruffvirus
KF493879.1 Mycobacterium phage Bernardo- complete genome Pipefishvirus Bclasvirinae
JN699003.1 Mycobacterium phage Athena- complete genome Pipefishvirus Bclasvirinae
JN698992.1 Mycobacterium phage Gadjet- complete genome Pipefishvirus Bclasvirinae
MH153810.1 Gordonia phage Sour- complete genome Sourvirus
MH744423.1 Mycobacterium phage Saguaro- complete genome Saguarovirus Bclasvirinae
MN617843.1 Mycobacterium phage Quesadilla- complete genome Quesadillavirus Bclasvirinae
MK059749.1 Mycobacterium phage CRB2- complete genome Quesadillavirus Bclasvirinae
KR997932.1 Mycobacterium phage Godines- complete genome Rosebushvirus Bclasvirinae
KF024722.1 Mycobacterium phage TA17A- complete genome Rosebushvirus Bclasvirinae
AY129334.1 Mycobacterium phage Rosebush- complete genome Rosebushvirus Bclasvirinae
MH632120.1 Mycobacterium phage Thonko- complete genome Thonkovirus Bclasvirinae
MN813686.1 Mycobacterium phage BirdsNest- complete genome Birdsnestvirus Bclasvirinae
MN813693.1 Mycobacterium phage Imvubu- complete genome Imvubuvirus Bclasvirinae
KJ433976.1 Mycobacterium phage Jolie1- complete genome Julieunavirus Bclasvirinae
KJ595576.1 Mycobacterium phage Manad- complete genome Pegunavirus Bclasvirinae
KR781349.1 Mycobacterium phage Apizium- complete genome Pegunavirus Bclasvirinae
JN192463.1 Mycobacterium phage Oline- complete genome Pegunavirus Bclasvirinae
KF279411.1 Mycobacterium phage Adawi- complete genome Coopervirus Bclasvirinae
KT270441.1 Mycobacterium phage BrownCNA- complete genome Coopervirus Bclasvirinae
KF279412.2 Mycobacterium phage Bane1- complete genome Coopervirus Bclasvirinae
MN284893.1 Mycobacterium phage LilMcDreamy- complete genome Lilmcdreamyvirus Bclasvirinae
KF024727.1 Mycobacterium phage Reprobate- complete genome Acadianvirus Bclasvirinae
KR080199.1 Mycobacterium phage Baee- complete genome Acadianvirus Bclasvirinae
JN699007.1 Mycobacterium virus Acadian- complete genome Acadianvirus Bclasvirinae
AY048850.1 Natrinema virus SNJ1- complete genome Yingchengvirus
BK013338.1 TPA asm Haloterrigena jeotgali icosahedral virus 1 isolate A29- complete genome Yingchengvirus
BK013337.1 TPA asm Natrinema versiforme icosahedral virus 1 isolate BOL5 4- complete genome Yingchengvirus
KR935217.1 Rhodobacter phage RcCronus- complete genome Cronusvirus
MF580957.1 Salinibacter phage M8CR30 2- complete genome Holosalinivirus
MF580955.1 Salinibacter phage M1EM 1- partial genome Holosalinivirus
JF712866.1 Vibrio phage phiVC8- complete genome Enhodamvirus
AY510084.1 Vibriophage VP5- complete genome Enhodamvirus
AY505112.1 Vibriophage VP2- complete genome Enhodamvirus
MH128984.1 Burkholderia phage vB BmuP KL4- complete genome Kelquatrovirus
AY605181.1 Burkholderia phage BcepC6B- complete genome Ryyoungvirus
AY029185.2 Bordetella phage BPP 1- complete genome Rauchvirus
DQ126339.2 Enterobacteria phage phiV10- complete genome Uetakevirus
JN391180.1 Salmonella phage SPN1S- complete genome Uetakevirus
AY150271.2 Enterobacteria phage epsilon15- complete genome Uetakevirus
MT732474.1 Tenacibaculum phage Gundel 1- complete genome Gundelvirus
KC821633.1 Cellulophaga phage phi13 2- complete genome Baltivirus
KC821622.1 Cellulophaga phage phi46 3- complete genome Bacelvirus
KC821620.1 Cellulophaga phage phi18 3- complete genome Baltivirus
KC821608.1 Cellulophaga phage phi19 3- complete genome Baltivirus
MT732432.1 Cellulophaga phage Calle 1- complete genome Callevirus
KC821614.1 Cellulophaga phage phi38 1- complete genome Callevirus
MT497068.1 Flavobacterium phage vB FspP elemoB 14 3B- partial genome Elemovirus
MT497067.1 Flavobacterium phage vB FspP elemoC 14 1A- partial genome Elemovirus
MT497017.1 Flavobacterium phage vB FspP elemoA 7 9A- complete genome Elemovirus
MZ130490.1 CrAss like virus sp. strain cr13 1- complete genome Kingevirus Doltivirinae
MT774393.1 CrAssphage cr127 1- complete genome Kahucivirus Doltivirinae
MZ130489.1 CrAss like virus sp. strain cr2 1- complete genome Jundunvirus Coarsevirinae
MZ130487.1 CrAss like virus sp. strain cr25 1- complete genome Jundunvirus Coarsevirinae
MT774384.1 CrAssphage cr8 1- complete genome Fohxhuevirus Obtuvirinae
MT774394.1 CrAssphage cr271 1- complete genome Hacihdavirus Obtuvirinae
MH552500.1 MAG CrAssphage sp. isolate ctcc615- complete genome Wotdevirus Obtuvirinae
MZ130484.1 CrAss like virus sp. strain cr54 1- complete genome Jahgtovirus Churivirinae
MZ130479.1 CrAss like virus sp. strain cr36 1- complete genome Jahgtovirus Churivirinae
MT774377.1 CrAssphage cr107 1- complete genome Jahgtovirus Churivirinae
MZ130476.1 CrAss like virus sp. strain cr123 1- complete genome Delmidovirus Crudevirinae
MT774386.1 CrAssphage cr110 1- complete genome Delmidovirus Crudevirinae
MT774383.1 CrAssphage cr11 1- complete genome Delmidovirus Crudevirinae
MZ130481.1 CrAss like virus sp. strain cr99 1- complete genome Carjivirus Crudevirinae
MZ130480.1 CrAss like virus sp. strain cr91 1- complete genome Drivevirus Crudevirinae
MH616963.1 MAG CrAssphage sp. isolate ctbg 1- complete genome Whopevirus Crudevirinae
MZ130486.1 CrAss like virus sp. strain cr9 1- complete genome Dabirmavirus Crudevirinae
MZ130488.1 CrAss like virus sp. strain cr17 1- complete genome Endlipuvirus Crudevirinae
MZ130474.1 CrAss like virus sp. strain cr49 1- complete genome Diorhovirus Crudevirinae
MT774381.1 CrAssphage cr3 1- complete genome Diorhovirus Crudevirinae

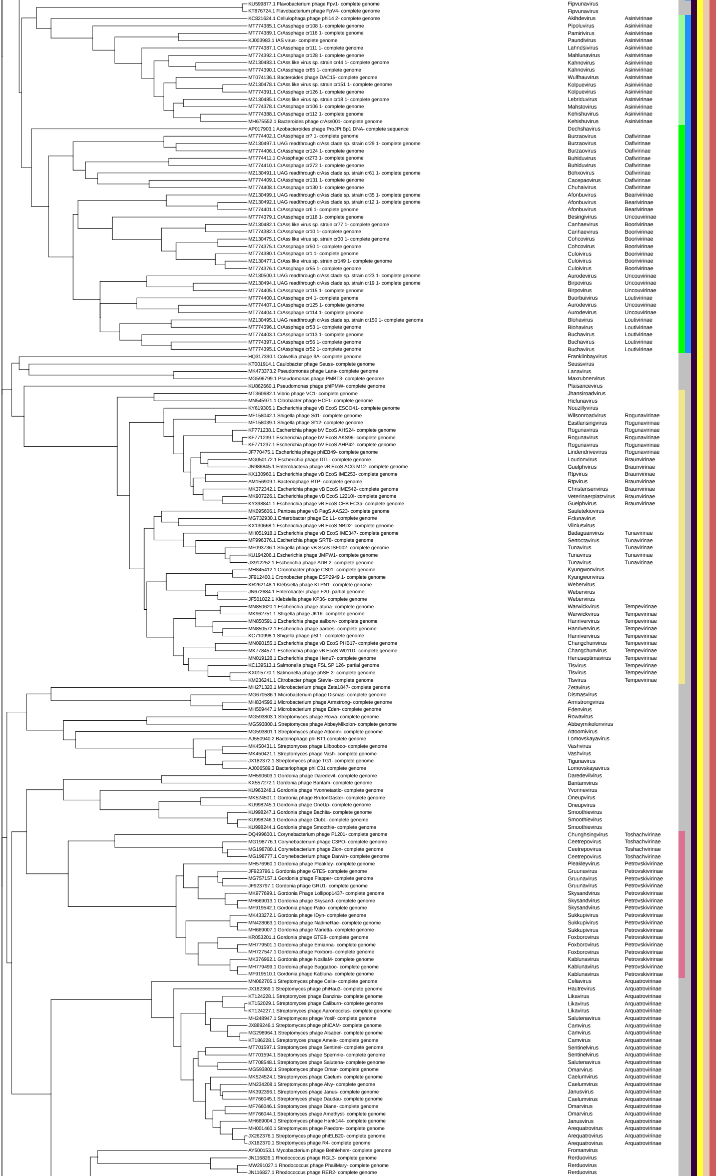

KU599877.1 Flavobacterium phage Fpv1- complete genome Fipvunavirus
KT876724.1 Flavobacterium phage FpV4- complete genome Fipvunavirus
KC821624.1 Cellulophaga phage phi14 2- complete genome Akihdevirus Asinivirinae
MT774385.1 CrAssphage cr108 1- complete genome Pipoluvirus Asinivirinae
MT774389.1 CrAssphage cr116 1- complete genome Pamirivirus Asinivirinae
KJ003983.1 IAS virus- complete genome Paundivirus Asinivirinae
MT774387.1 CrAssphage cr111 1- complete genome Lahndsivirus Asinivirinae
MT774392.1 CrAssphage cr128 1- complete genome Mahlunavirus Asinivirinae
MZ130483.1 CrAss like virus sp. strain cr44 1- complete genome Kahnovirus Asinivirinae
MT774390.1 CrAssphage cr85 1- complete genome Kahnovirus Asinivirinae
MT074136.1 Bacteroides phage DAC15- complete genome Wulfhauvirus Asinivirinae
MZ130478.1 CrAss like virus sp. strain cr151 1- complete genome Kolpuevirus Asinivirinae
MT774391.1 CrAssphage cr126 1- complete genome Kolpuevirus Asinivirinae
MZ130485.1 CrAss like virus sp. strain cr18 1- complete genome Lebriduvirus Asinivirinae
MT774378.1 CrAssphage cr106 1- complete genome Mahstovirus Asinivirinae
MT774388.1 CrAssphage cr112 1- complete genome Kehishuvirus Asinivirinae
MH675552.1 Bacteroides phage crAss001- complete genome Kehishuvirus Asinivirinae
AP017903.1 Azobacteroides phage ProJPt Bp1 DNA- complete sequence Dechshavirus
MT774402.1 CrAssphage cr7 1- complete genome Burzaovirus Oafivirinae
MZ130497.1 UAG readthrough crAss clade sp. strain cr29 1- complete genome Burzaovirus Oafivirinae
MT774406.1 CrAssphage cr124 1- complete genome Burzaovirus Oafivirinae
MT774411.1 CrAssphage cr273 1- complete genome Buhlduvirus Oafivirinae
MT774410.1 CrAssphage cr272 1- complete genome Buhlduvirus Oafivirinae
MZ130491.1 UAG readthrough crAss clade sp. strain cr61 1- complete genome Bohxovirus Oafivirinae
MT774409.1 CrAssphage cr131 1- complete genome Cacepaovirus Oafivirinae
MT774408.1 CrAssphage cr130 1- complete genome Chuhaivirus Oafivirinae
MZ130499.1 UAG readthrough crAss clade sp. strain cr35 1- complete genome Afonbuvirus Bearivirinae
MZ130492.1 UAG readthrough crAss clade sp. strain cr12 1- complete genome Afonbuvirus Bearivirinae
MT774401.1 CrAssphage cr6 1- complete genome Afonbuvirus Bearivirinae
MT774379.1 CrAssphage cr118 1- complete genome Besingivirus Uncouvirinae
MZ130482.1 CrAss like virus sp. strain cr77 1- complete genome Canhaevirus Boorivirinae
MT774382.1 CrAssphage cr10 1- complete genome Canhaevirus Boorivirinae
MZ130475.1 CrAss like virus sp. strain cr30 1- complete genome Cohcovirus Boorivirinae
MT774375.1 CrAssphage cr50 1- complete genome Cohcovirus Boorivirinae
MT774380.1 CrAssphage cr1 1- complete genome Culoivirus Boorivirinae
MZ130477.1 CrAss like virus sp. strain cr149 1- complete genome Culoivirus Boorivirinae
MT774376.1 CrAssphage cr55 1- complete genome Culoivirus Boorivirinae
MZ130500.1 UAG readthrough crAss clade sp. strain cr23 1- complete genome Aurodevirus Uncouvirinae
MZ130494.1 UAG readthrough crAss clade sp. strain cr19 1- complete genome Birpovirus Uncouvirinae
MT774405.1 CrAssphage cr115 1- complete genome Birpovirus Uncouvirinae
MT774400.1 CrAssphage cr4 1- complete genome Buorbuivirus Loutivirinae
MT774407.1 CrAssphage cr125 1- complete genome Aurodevirus Uncouvirinae
MT774404.1 CrAssphage cr114 1- complete genome Aurodevirus Uncouvirinae
MZ130495.1 UAG readthrough crAss clade sp. strain cr150 1- complete genome Blohavirus Loutivirinae
MT774396.1 CrAssphage cr53 1- complete genome Blohavirus Loutivirinae
MT774403.1 CrAssphage cr113 1- complete genome Buchavirus Loutivirinae
MT774397.1 CrAssphage cr56 1- complete genome Buchavirus Loutivirinae
MT774395.1 CrAssphage cr52 1- complete genome Buchavirus Loutivirinae
HQ317390.1 Colwellia phage 9A- complete genome Franklinbayvirus
KT001914.1 Caulobacter phage Seuss- complete genome Seussvirus
MK473373.2 Pseudomonas phage Lana- complete genome Lanavirus
MG596799.1 Pseudomonas phage PMBT3- complete genome Maxrubnervirus
KU862660.1 Pseudomonas phage phiPMW- complete genome Plaisancevirus
MT360682.1 Vibrio phage VC1- complete genome Jhansiroadvirus
MN545971.1 Citrobacter phage HCF1- complete genome Hicfunavirus
KY619305.1 Escherichia phage vB EcoS ESCO41- complete genome Nouzillyvirus
MF158042.1 Shigella phage Sd1- complete genome Wilsonroadvirus Rogunavirinae
MF158039.1 Shigella phage Sf12- complete genome Eastlansingvirus Rogunavirinae
KF771238.1 Escherichia phage bV EcoS AHS24- complete genome Rogunavirus Rogunavirinae
KF771239.1 Escherichia phage bV EcoS AKS96- complete genome Rogunavirus Rogunavirinae
KF771237.1 Escherichia phage bV EcoS AHP42- complete genome Rogunavirus Rogunavirinae
JF770475.1 Escherichia phage phiEB49- complete genome Lindendrivevirus Rogunavirinae
MG050172.1 Escherichia phage DTL- complete genome Loudonvirus Braunvirinae
JN986845.1 Enterobacteria phage vB EcoS ACG M12- complete genome Guelphvirus Braunvirinae
KX130960.1 Escherichia phage vB EcoS IME253- complete genome Rtpvirus Braunvirinae
AM156909.1 Bacteriophage RTP- complete genome Rtpvirus Braunvirinae
MK372342.1 Escherichia phage vB EcoS IME542- complete genome Christensenvirus Braunvirinae
MK907226.1 Escherichia phage vB EcoS 12210I- complete genome Veterinaerplatzvirus Braunvirinae
KY398841.1 Escherichia phage vB EcoS CEB EC3a- complete genome Guelphvirus Braunvirinae
MK095606.1 Pantoea phage vB PagS AAS23- complete genome Sauletekiovirus
MG732930.1 Enterobacter phage Ec L1- complete genome Eclunavirus
KX130668.1 Escherichia phage vB EcoS NBD2- complete genome Vilniusvirus
MH051918.1 Escherichia phage vB EcoS IME347- complete genome Badaguanvirus Tunavirinae
MF996376.1 Escherichia phage SRT8- complete genome Sertoctavirus Tunavirinae
MF093736.1 Shigella phage vB SsoS ISF002- complete genome Tunavirus Tunavirinae
KU194206.1 Escherichia phage JMPW1- complete genome Tunavirus Tunavirinae
JX912252.1 Escherichia phage ADB 2- complete genome Tunavirus Tunavirinae
MH845412.1 Cronobacter phage CS01- complete genome Kyungwonvirus
JF912400.1 Cronobacter phage ESP2949 1- complete genome Kyungwonvirus
KR262148.1 Klebsiella phage KLPN1- complete genome Webervirus
JN672684.1 Enterobacter phage F20- partial genome Webervirus
JF501022.1 Klebsiella phage KP36- complete genome Webervirus
MN850620.1 Escherichia phage atuna- complete genome Warwickvirus Tempevirinae
MK962751.1 Shigella phage JK16- complete genome Warwickvirus Tempevirinae
MN850591.1 Escherichia phage aalborv- complete genome Hanrivervirus Tempevirinae
MN850572.1 Escherichia phage aaroes- complete genome Hanrivervirus Tempevirinae
KC710998.1 Shigella phage pSf 1- complete genome Hanrivervirus Tempevirinae
MN090155.1 Escherichia phage vB EcoS PHB17- complete genome Changchunvirus Tempevirinae
MK778457.1 Escherichia phage vB EcoS W011D- complete genome Changchunvirus Tempevirinae
MN019128.1 Escherichia phage Henu7- complete genome Henuseptimavirus Tempevirinae
KC139513.1 Salmonella phage FSL SP 126- partial genome Tlsvirus Tempevirinae
KX015770.1 Salmonella phage phSE 2- complete genome Tlsvirus Tempevirinae
KM236241.1 Citrobacter phage Stevie- complete genome Tlsvirus Tempevirinae
MH271320.1 Microbacterium phage Zeta1847- complete genome Zetavirus
MG670586.1 Microbacterium phage Dismas- complete genome Dismasvirus
MH834596.1 Microbacterium phage Armstrong- complete genome Armstrongvirus
MH509447.1 Microbacterium phage Eden- complete genome Edenvirus
MG593803.1 Streptomyces phage Rowa- complete genome Rowavirus
MG593800.1 Streptomyces phage AbbeyMikolon- complete genome Abbeymikolonvirus
MG593801.1 Streptomyces phage Attoomi- complete genome Attoomivirus
AJ550940.2 Bacteriophage phi BT1 complete genome Lomovskayavirus
MK450431.1 Streptomyces phage Lilbooboo- complete genome Vashvirus
MK450421.1 Streptomyces phage Vash- complete genome Vashvirus
JX182372.1 Streptomyces phage TG1- complete genome Tigunavirus
AJ006589.3 Bacteriophage phi C31 complete genome Lomovskayavirus
MH590603.1 Gordonia phage Daredevil- complete genome Daredevilvirus
KX557272.1 Gordonia phage Bantam- complete genome Bantamvirus
KU963248.1 Gordonia phage Yvonnetastic- complete genome Yvonnevirus
MK524501.1 Gordonia phage BrutonGaster- complete genome Oneupvirus
KU998245.1 Gordonia phage OneUp- complete genome Oneupvirus
KU998247.1 Gordonia phage Bachita- complete genome Smoothievirus
KU998246.1 Gordonia phage ClubL- complete genome Smoothievirus
KU998244.1 Gordonia phage Smoothie- complete genome Smoothievirus
DQ499600.1 Corynebacterium phage P1201- complete genome Chunghsingvirus Toshachvirinae
MG198776.1 Corynebacterium phage C3PO- complete genome Ceetrepovirus Toshachvirinae
MG198780.1 Corynebacterium phage Zion- complete genome Ceetrepovirus Toshachvirinae
MG198777.1 Corynebacterium phage Darwin- complete genome Ceetrepovirus Toshachvirinae
MH576960.1 Gordonia phage Pleakley- complete genome Pleakleyvirus Petrovskivirinae
JF923796.1 Gordonia phage GTE5- complete genome Gruunavirus Petrovskivirinae
MG757157.1 Gordonia phage Flapper- complete genome Gruunavirus Petrovskivirinae
JF923797.1 Gordonia phage GRU1- complete genome Gruunavirus Petrovskivirinae
MK977699.1 Gordonia Phage Lollipop1437- complete genome Skysandvirus Petrovskivirinae
MH669013.1 Gordonia phage Skysand- complete genome Skysandvirus Petrovskivirinae
MF919542.1 Gordonia phage Patio- complete genome Skysandvirus Petrovskivirinae
MK433272.1 Gordonia phage IDyn- complete genome Sukkupivirus Petrovskivirinae
MN428063.1 Gordonia phage NadineRae- complete genome Sukkupivirus Petrovskivirinae
MH669007.1 Gordonia phage Marietta- complete genome Sukkupivirus Petrovskivirinae
KR053201.1 Gordonia phage GTE8- complete genome Foxborovirus Petrovskivirinae
MH779501.1 Gordonia phage Emianna- complete genome Foxborovirus Petrovskivirinae
MH727547.1 Gordonia phage Foxboro- complete genome Foxborovirus Petrovskivirinae
MK376962.1 Gordonia phage NosilaM- complete genome Kablunavirus Petrovskivirinae
MH779499.1 Gordonia phage Buggaboo- complete genome Kablunavirus Petrovskivirinae
MF919510.1 Gordonia phage Kabluna- complete genome Kablunavirus Petrovskivirinae
MN062705.1 Streptomyces phage Celia- complete genome Celiavirus Arquatrovirinae
JX182369.1 Streptomyces phage phiHau3- complete genome Hautrevirus Arquatrovirinae
KT124228.1 Streptomyces phage Danzina- complete genome Likavirus Arquatrovirinae
KT152029.1 Streptomyces phage Caliburn- complete genome Likavirus Arquatrovirinae
KT124227.1 Streptomyces phage Aaronocolus- complete genome Likavirus Arquatrovirinae
MH248947.1 Streptomyces phage Yosif- complete genome Salutenavirus Arquatrovirinae
JX889246.1 Streptomyces phage phiCAM- complete genome Camvirus Arquatrovirinae
MG298964.1 Streptomyces phage Alsaber- complete genome Camvirus Arquatrovirinae
KT186228.1 Streptomyces phage Amela- complete genome Camvirus Arquatrovirinae
MT701597.1 Streptomyces phage Sentinel- complete genome Sentinelvirus Arquatrovirinae
MT701594.1 Streptomyces phage Spernnie- complete genome Sentinelvirus Arquatrovirinae
MT708548.1 Streptomyces phage Salutena- complete genome Salutenavirus Arquatrovirinae
MG593802.1 Streptomyces phage Omar- complete genome Omarvirus Arquatrovirinae
MK524524.1 Streptomyces phage Caelum- complete genome Caelumvirus Arquatrovirinae
MN234208.1 Streptomyces phage Alvy- complete genome Caelumvirus Arquatrovirinae
MK392366.1 Streptomyces phage Janus- complete genome Janusvirus Arquatrovirinae
MF766045.1 Streptomyces phage Daudau- complete genome Caelumvirus Arquatrovirinae
MF766046.1 Streptomyces phage Diane- complete genome Omarvirus Arquatrovirinae
MF766044.1 Streptomyces phage Amethyst- complete genome Omarvirus Arquatrovirinae
MH669004.1 Streptomyces phage Hank144- complete genome Janusvirus Arquatrovirinae
MH001460.1 Streptomyces phage Paedore- complete genome Arequatrovirus Arquatrovirinae
JX262376.1 Streptomyces phage phiELB20- complete genome Arequatrovirus Arquatrovirinae
JX182370.1 Streptomyces phage R4- complete genome Arequatrovirus Arquatrovirinae
AY500153.1 Mycobacterium phage Bethlehem- complete genome Fromanvirus
JN116826.1 Rhodococcus phage RGL3- complete genome Rerduovirus
MW291027.1 Rhodococcus phage PhailMary- complete genome Rerduovirus
JN116827.1 Rhodococcus phage RER2- complete genome Rerduovirus

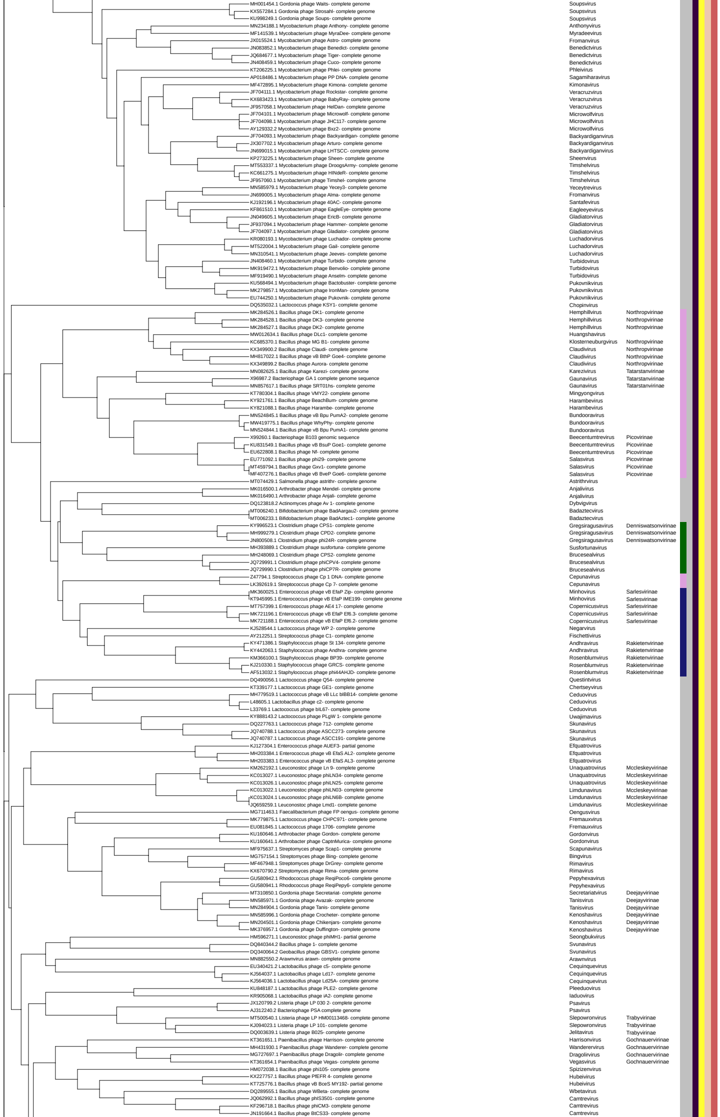
MH001454.1 Gordonia phage Waits- complete genome Soupsvirus
KX557284.1 Gordonia phage Strosahl- complete genome Soupsvirus
KU998249.1 Gordonia phage Soups- complete genome Soupsvirus
MN234188.1 Mycobacterium phage Anthony- complete genome Anthonyvirus
MF141539.1 Mycobacterium phage MyraDee- complete genome Myradeevirus
JX015524.1 Mycobacterium phage Astro- complete genome Fromanvirus
JN083852.1 Mycobacterium phage Benedict- complete genome Benedictvirus
JQ684677.1 Mycobacterium phage Tiger- complete genome Benedictvirus
JN408459.1 Mycobacterium phage Cuco- complete genome Benedictvirus
KT206225.1 Mycobacterium phage Phlei- complete genome Phleivirus
AP018486.1 Mycobacterium phage PP DNA- complete genome Sagamiharavirus
MF472895.1 Mycobacterium phage Kimona- complete genome Kimonavirus
JF704111.1 Mycobacterium phage Rockstar- complete genome Veracruzvirus
KX683423.1 Mycobacterium phage BabyRay- complete genome Veracruzvirus
JF957058.1 Mycobacterium phage HelDan- complete genome Veracruzvirus
JF704101.1 Mycobacterium phage Microwolf- complete genome Microwolfvirus
JF704098.1 Mycobacterium phage JHC117- complete genome Microwolfvirus
AY129332.2 Mycobacterium phage Bxz2- complete genome Microwolfvirus
JF704093.1 Mycobacterium phage Backyardigan- complete genome Backyardiganvirus
JX307702.1 Mycobacterium phage Arturo- complete genome Backyardiganvirus
JN699015.1 Mycobacterium phage LHTSCC- complete genome Backyardiganvirus
KP273225.1 Mycobacterium phage Sheen- complete genome Sheenvirus
MT553337.1 Mycobacterium phage DroogsArmy- complete genome Timshelvirus
KC661275.1 Mycobacterium phage HINdeR- complete genome Timshelvirus
JF957060.1 Mycobacterium phage Timshel- complete genome Timshelvirus
MN585979.1 Mycobacterium phage Yecey3- complete genome Yeceytrevirus
JN699005.1 Mycobacterium phage Alma- complete genome Fromanvirus
KJ192196.1 Mycobacterium phage 40AC- complete genome Santafevirus
KF861510.1 Mycobacterium phage EagleEye- complete genome Eagleeyevirus
JN049605.1 Mycobacterium phage EricB- complete genome Gladiatorvirus
JF937094.1 Mycobacterium phage Hammer- complete genome Gladiatorvirus
JF704097.1 Mycobacterium phage Gladiator- complete genome Gladiatorvirus
KR080193.1 Mycobacterium phage Luchador- complete genome Luchadorvirus
MT522004.1 Mycobacterium phage Gail- complete genome Luchadorvirus
MN310541.1 Mycobacterium phage Jeeves- complete genome Luchadorvirus
JN408460.1 Mycobacterium phage Turbido- complete genome Turbidovirus
MK919472.1 Mycobacterium phage Benvolio- complete genome Turbidovirus
MF919490.1 Mycobacterium phage Anselm- complete genome Turbidovirus
KU568494.1 Mycobacterium phage Bactobuster- complete genome Pukovnikvirus
MK279857.1 Mycobacterium phage IronMan- complete genome Pukovnikvirus
EU744250.1 Mycobacterium phage Pukovnik- complete genome Pukovnikvirus
DQ535032.1 Lactococcus phage KSY1- complete genome Chopinvirus
MK284526.1 Bacillus phage DK1- complete genome Hemphillvirus Northropvirinae
MK284528.1 Bacillus phage DK3- complete genome Hemphillvirus Northropvirinae
MK284527.1 Bacillus phage DK2- complete genome Hemphillvirus Northropvirinae
MW012634.1 Bacillus phage DLc1- complete genome Huangshavirus
KC685370.1 Bacillus phage MG B1- complete genome Klosterneuburgvirus Northropvirinae
KX349900.2 Bacillus phage Claudi- complete genome Claudivirus Northropvirinae
MH817022.1 Bacillus phage vB BthP Goe4- complete genome Claudivirus Northropvirinae
KX349899.2 Bacillus phage Aurora- complete genome Claudivirus Northropvirinae
MN082625.1 Bacillus phage Karezi- complete genome Karezivirus Tatarstanvirinae
X96987.2 Bacteriophage GA 1 complete genome sequence Gaunavirus Tatarstanvirinae
MN857617.1 Bacillus phage SRT01hs- complete genome Gaunavirus Tatarstanvirinae
KT780304.1 Bacillus phage VMY22- complete genome Mingyongvirus
KY921761.1 Bacillus phage BeachBum- complete genome Harambevirus
KY821088.1 Bacillus phage Harambe- complete genome Harambevirus
MN524845.1 Bacillus phage vB Bpu PumA2- complete genome Bundooravirus
MW419775.1 Bacillus phage WhyPhy- complete genome Bundooravirus
MN524844.1 Bacillus phage vB Bpu PumA1- complete genome Bundooravirus
X99260.1 Bacteriophage B103 genomic sequence Beecentumtrevirus Picovirinae
KU831549.1 Bacillus phage vB BsuP Goe1- complete genome Beecentumtrevirus Picovirinae
EU622808.1 Bacillus phage Nf- complete genome Beecentumtrevirus Picovirinae
EU771092.1 Bacillus phage phi29- complete genome Salasvirus Picovirinae
MT459794.1 Bacillus phage Gxv1- complete genome Salasvirus Picovirinae
MF407276.1 Bacillus phage vB BveP Goe6- complete genome Salasvirus Picovirinae
MT074429.1 Salmonella phage astrithr- complete genome Astrithrvirus
MK016500.1 Arthrobacter phage Mendel- complete genome Anjalivirus
MK016490.1 Arthrobacter phage Anjali- complete genome Anjalivirus
DQ123818.2 Actinomyces phage Av 1- complete genome Dybvigvirus
MT006240.1 Bifidobacterium phage BadAargau2- complete genome Badaztecvirus
MT006233.1 Bifidobacterium phage BadAztec1- complete genome Badaztecvirus
KY996523.1 Clostridium phage CPS1- complete genome Gregsiragusavirus Denniswatsonvirinae
MH999279.1 Clostridium phage CPD2- complete genome Gregsiragusavirus Denniswatsonvirinae
JN800508.1 Clostridium phage phi24R- complete genome Gregsiragusavirus Denniswatsonvirinae
MH393889.1 Clostridium phage susfortuna- complete genome Susfortunavirus
MH248069.1 Clostridium phage CPS2- complete genome Brucesealvirus
JQ729991.1 Clostridium phage phiCPV4- complete genome Brucesealvirus
JQ729990.1 Clostridium phage phiCP7R- complete genome Brucesealvirus
Z47794.1 Streptococcus phage Cp 1 DNA- complete genome Cepunavirus
LK392619.1 Streptococcus phage Cp 7- complete genome Cepunavirus
MK360025.1 Enterococcus phage vB EfaP Zip- complete genome Minhovirus Sarlesvirinae
KT945995.1 Enterococcus phage vB EfaP IME199- complete genome Minhovirus Sarlesvirinae
MT757399.1 Enterococcus phage AE4 17- complete genome Copernicusvirus Sarlesvirinae
MK721196.1 Enterococcus phage vB EfaP Ef6.3- complete genome Copernicusvirus Sarlesvirinae
MK721188.1 Enterococcus phage vB EfaP Ef6.2- complete genome Copernicusvirus Sarlesvirinae
KJ528544.1 Lactococcus phage WP 2- complete genome Negarvirus
AY212251.1 Streptococcus phage C1- complete genome Fischettivirus
KY471386.1 Staphylococcus phage St 134- complete genome Andhravirus Rakietenvirinae
KY442063.1 Staphylococcus phage Andhra- complete genome Andhravirus Rakietenvirinae
KM366100.1 Staphylococcus phage BP39- complete genome Rosenblumvirus Rakietenvirinae
KJ210330.1 Staphylococcus phage GRCS- complete genome Rosenblumvirus Rakietenvirinae
AF513032.1 Staphylococcus phage phi44AHJD- complete genome Rosenblumvirus Rakietenvirinae
DQ490056.1 Lactococcus phage Q54- complete genome Questintvirus
KT339177.1 Lactococcus phage GE1- complete genome Chertseyvirus
MH779519.1 Lactococcus phage vB LLc bIBB14- complete genome Ceduovirus
L48605.1 Lactobacillus phage c2- complete genome Ceduovirus
L33769.1 Lactococcus phage bIL67- complete genome Ceduovirus
KY888143.2 Lactococcus phage PLgW 1- complete genome Uwajimavirus
DQ227763.1 Lactococcus phage 712- complete genome Skunavirus
JQ740788.1 Lactococcus phage ASCC273- complete genome Skunavirus
JQ740787.1 Lactococcus phage ASCC191- complete genome Skunavirus
KJ127304.1 Enterococcus phage AUEF3- partial genome Efquatrovirus
MH203384.1 Enterococcus phage vB EfaS AL2- complete genome Efquatrovirus
MH203383.1 Enterococcus phage vB EfaS AL3- complete genome Efquatrovirus
KM262192.1 Leuconostoc phage Ln 9- complete genome Unaquatrovirus Mccleskeyvirinae
KC013027.1 Leuconostoc phage phiLN34- complete genome Unaquatrovirus Mccleskeyvirinae
KC013026.1 Leuconostoc phage phiLN25- complete genome Unaquatrovirus Mccleskeyvirinae
KC013022.1 Leuconostoc phage phiLN03- complete genome Limdunavirus Mccleskeyvirinae
KC013024.1 Leuconostoc phage phiLN6B- complete genome Limdunavirus Mccleskeyvirinae
JQ659259.1 Leuconostoc phage Lmd1- complete genome Limdunavirus Mccleskeyvirinae
MG711463.1 Faecalibacterium phage FP oengus- complete genome Oengusvirus
MK779875.1 Lactococcus phage CHPC971- complete genome Fremauxvirus
EU081845.1 Lactococcus phage 1706- complete genome Fremauxvirus
KU160646.1 Arthrobacter phage Gordon- complete genome Gordonvirus
KU160641.1 Arthrobacter phage CaptnMurica- complete genome Gordonvirus
MF975637.1 Streptomyces phage Scap1- complete genome Scapunavirus
MG757154.1 Streptomyces phage Bing- complete genome Bingvirus
MF467948.1 Streptomyces phage DrGrey- complete genome Rimavirus
KX670790.2 Streptomyces phage Rima- complete genome Rimavirus
GU580942.1 Rhodococcus phage ReqiPoco6- complete genome Pepyhexavirus
GU580941.1 Rhodococcus phage ReqiPepy6- complete genome Pepyhexavirus
MT310850.1 Gordonia phage Secretariat- complete genome Secretariatvirus Deejayvirinae
MN585971.1 Gordonia phage Avazak- complete genome Tanisvirus Deejayvirinae
MN284904.1 Gordonia phage Tanis- complete genome Tanisvirus Deejayvirinae
MN585996.1 Gordonia phage Crocheter- complete genome Kenoshavirus Deejayvirinae
MN204501.1 Gordonia phage Chikenjars- complete genome Kenoshavirus Deejayvirinae
MK376957.1 Gordonia phage Duffington- complete genome Kenoshavirus Deejayvirinae
HM596271.1 Leuconostoc phage phiMH1- partial genome Seongbukvirus
DQ840344.2 Bacillus phage 1- complete genome Svunavirus
DQ340064.2 Geobacillus phage GBSV1- complete genome Svunavirus
MN882550.2 Arawnvirus arawn- complete genome Arawnvirus
EU340421.2 Lactobacillus phage c5- complete genome Cequinquevirus
KJ564037.1 Lactobacillus phage Ld17- complete genome Cequinquevirus
KJ564036.1 Lactobacillus phage Ld25A- complete genome Cequinquevirus
KU848187.1 Lactobacillus phage PLE2- complete genome Pleeduovirus
KR905068.1 Lactobacillus phage iA2- complete genome Iaduovirus
JX120799.2 Listeria phage LP 030 2- complete genome Psavirus
AJ312240.2 Bacteriophage PSA complete genome Psavirus
MT500540.1 Listeria phage LP HM00113468- complete genome Slepowronvirus Trabyvirinae
KJ094023.1 Listeria phage LP 101- complete genome Slepowronvirus Trabyvirinae
DQ003639.1 Listeria phage B025- complete genome Jelitavirus Trabyvirinae
KT361651.1 Paenibacillus phage Harrison- complete genome Harrisonvirus Gochnauervirinae
MH431930.1 Paenibacillus phage Wanderer- complete genome Wanderervirus Gochnauervirinae
MG727697.1 Paenibacillus phage Dragolir- complete genome Dragolirvirus Gochnauervirinae
KT361654.1 Paenibacillus phage Vegas- complete genome Vegasvirus Gochnauervirinae
HM072038.1 Bacillus phage phi105- complete genome Spizizenvirus
KX227757.1 Bacillus phage PfEFR 4- complete genome Hubeivirus
KT725776.1 Bacillus phage vB BceS MY192- partial genome Hubeivirus
DQ289555.1 Bacillus phage WBeta- complete genome Wbetavirus
JQ062992.1 Bacillus phage phIS3501- complete genome Camtrevirus
KF296718.1 Bacillus phage phiCM3- complete genome Camtrevirus
JN191664.1 Bacillus phage BtCS33- complete genome Camtrevirus

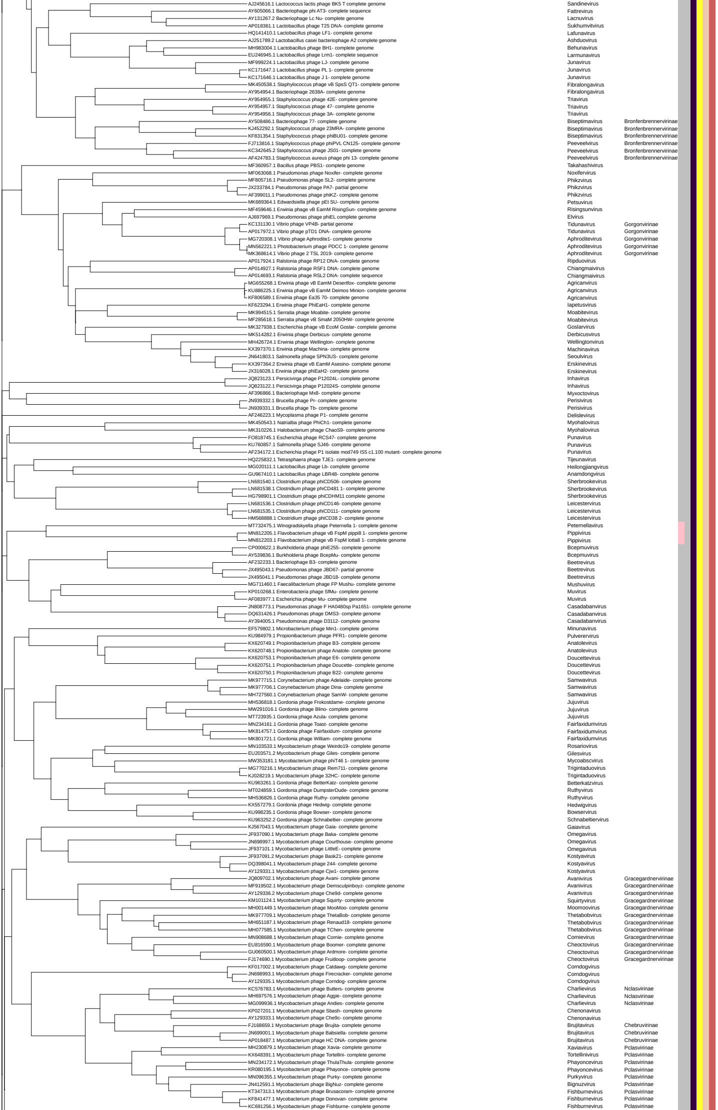

AJ245616.1 Lactococcus lactis phage BK5 T complete genome Sandinevirus
AY605066.1 Bacteriophage phi AT3- complete sequence Fattrevirus
AY131267.2 Bacteriophage Lc Nu- complete genome Lacnuvirus
AP018361.1 Lactobacillus phage T25 DNA- complete genome Sukhumvitvirus
HQ141410.1 Lactobacillus phage LF1- complete genome Lafunavirus
AJ251789.2 Lactobacillus casei bacteriophage A2 complete genome Ashduovirus
MH983004.1 Lactobacillus phage BH1- complete genome Behunavirus
EU246945.1 Lactobacillus phage Lrm1- complete sequence Larmunavirus
MF999224.1 Lactobacillus phage LJ- complete genome Junavirus
KC171647.1 Lactobacillus phage PL 1- complete genome Junavirus
KC171646.1 Lactobacillus phage J 1- complete genome Junavirus
MK450538.1 Staphylococcus phage vB SpsS QT1- complete genome Fibralongavirus
AY954954.1 Bacteriophage 2638A- complete genome Fibralongavirus
AY954955.1 Staphylococcus phage 42E- complete genome Triavirus
AY954957.1 Staphylococcus phage 47- complete genome Triavirus
AY954956.1 Staphylococcus phage 3A- complete genome Triavirus
AY508486.1 Bacteriophage 77- complete genome Biseptimavirus Bronfenbrennervirinae
KJ452292.1 Staphylococcus phage 23MRA- complete genome Biseptimavirus Bronfenbrennervirinae
KF831354.1 Staphylococcus phage phiBU01- complete genome Biseptimavirus Bronfenbrennervirinae
FJ713816.1 Staphylococcus phage phiPVL CN125- complete genome Peeveelvirus Bronfenbrennervirinae
KC342645.2 Staphylococcus phage JS01- complete genome Peeveelvirus Bronfenbrennervirinae
AF424783.1 Staphylococcus aureus phage phi 13- complete genome Peeveelvirus Bronfenbrennervirinae
MF360957.1 Bacillus phage PBS1- complete genome Takahashivirus
MF063068.1 Pseudomonas phage Noxifer- complete genome Noxifervirus
MF805716.1 Pseudomonas phage SL2- complete genome Phikzvirus
JX233784.1 Pseudomonas phage PA7- partial genome Phikzvirus
AF399011.1 Pseudomonas phage phiKZ- complete genome Phikzvirus
MK689364.1 Edwardsiella phage pEt SU- complete genome Petsuvirus
MF459646.1 Erwinia phage vB EamM RisingSun- complete genome Risingsunvirus
AJ697969.1 Pseudomonas phage phiEL complete genome Elvirus
KC131130.1 Vibrio phage VP4B- partial genome Tidunavirus Gorgonvirinae
AP017972.1 Vibrio phage pTD1 DNA- complete genome Tidunavirus Gorgonvirinae
MG720308.1 Vibrio phage Aphrodite1- complete genome Aphroditevirus Gorgonvirinae
MN562221.1 Photobacterium phage PDCC 1- complete genome Aphroditevirus Gorgonvirinae
MK368614.1 Vibrio phage 2 TSL 2019- complete genome Aphroditevirus Gorgonvirinae
AP017924.1 Ralstonia phage RP12 DNA- complete genome Ripduovirus
AP014927.1 Ralstonia phage RSF1 DNA- complete genome Chiangmaivirus
AP014693.1 Ralstonia phage RSL2 DNA- complete sequence Chiangmaivirus
MG655268.1 Erwinia phage vB EamM Desertfox- complete genome Agricanvirus
KU886225.1 Erwinia phage vB EamM Deimos Minion- complete genome Agricanvirus
KF806589.1 Erwinia phage Ea35 70- complete genome Agricanvirus
KF623294.1 Erwinia phage PhiEaH1- complete genome Iapetusvirus
MK994515.1 Serratia phage Moabite- complete genome Moabitevirus
MF285618.1 Serratia phage vB SmaM 2050HW- complete genome Moabitevirus
MK327938.1 Escherichia phage vB EcoM Goslar- complete genome Goslarvirus
MK514282.1 Erwinia phage Derbicus- complete genome Derbicusvirus
MH426724.1 Erwinia phage Wellington- complete genome Wellingtonvirus
KX397370.1 Erwinia phage Machina- complete genome Machinavirus
JN641803.1 Salmonella phage SPN3US- complete genome Seoulvirus
KX397364.2 Erwinia phage vB EamM Asesino- complete genome Erskinevirus
JX316028.1 Erwinia phage phiEaH2- complete genome Erskinevirus
JQ823123.1 Persicivirga phage P12024L- complete genome Inhavirus
JQ823122.1 Persicivirga phage P12024S- complete genome Inhavirus
AF396866.1 Bacteriophage Mx8- complete genome Myxoctovirus
JN939332.1 Brucella phage Pr- complete genome Perisivirus
JN939331.1 Brucella phage Tb- complete genome Perisivirus
AF246223.1 Mycoplasma phage P1- complete genome Delislevirus
MK450543.1 Natrialba phage PhiCh1- complete genome Myohalovirus
MK310226.1 Halobacterium phage ChaoS9- complete genome Myohalovirus
FO818745.1 Escherichia phage RCS47- complete genome Punavirus
KU760857.1 Salmonella phage SJ46- complete genome Punavirus
AF234172.1 Escherichia phage P1 isolate mod749 IS5 c1.100 mutant- complete genome Punavirus
HQ225832.1 Tetrasphaera phage TJE1- complete genome Tijeunavirus
MG020111.1 Lactobacillus phage Lb- complete genome Heilongjiangvirus
GU967410.1 Lactobacillus phage LBR48- complete genome Anamdongvirus
LN681540.1 Clostridium phage phiCD506- complete genome Sherbrookevirus
LN681538.1 Clostridium phage phiCD481 1- complete genome Sherbrookevirus
HG798901.1 Clostridium phage phiCDHM11 complete genome Sherbrookevirus
LN681536.1 Clostridium phage phiCD146- complete genome Leicestervirus
LN681535.1 Clostridium phage phiCD111- complete genome Leicestervirus
HM568888.1 Clostridium phage phiCD38 2- complete genome Leicestervirus
MT732475.1 Winogradskyella phage Peternella 1- complete genome Peternellavirus
MN812205.1 Flavobacterium phage vB FspM pippi8 1- complete genome Pippivirus
MN812203.1 Flavobacterium phage vB FspM lotta8 1- complete genome Pippivirus
CP000622.1 Burkholderia phage phiE255- complete genome Bcepmuvirus
AY539836.1 Burkholderia phage BcepMu- complete genome Bcepmuvirus
AF232233.1 Bacteriophage B3- complete genome Beetrevirus
JX495043.1 Pseudomonas phage JBD67- partial genome Beetrevirus
JX495041.1 Pseudomonas phage JBD18- complete genome Beetrevirus
MG711460.1 Faecalibacterium phage FP Mushu- complete genome Mushuvirus
KP010268.1 Enterobacteria phage SfMu- complete genome Muvirus
AF083977.1 Escherichia phage Mu- complete genome Muvirus
JN808773.1 Pseudomonas phage F HA0480sp Pa1651- complete genome Casadabanvirus
DQ631426.1 Pseudomonas phage DMS3- complete genome Casadabanvirus
AY394005.1 Pseudomonas phage D3112- complete genome Casadabanvirus
EF579802.1 Microbacterium phage Min1- complete genome Minunavirus
KU984979.1 Propionibacterium phage PFR1- complete genome Pulverervirus
KX620749.1 Propionibacterium phage B3- complete genome Anatolevirus
KX620748.1 Propionibacterium phage Anatole- complete genome Anatolevirus
KX620753.1 Propionibacterium phage E6- complete genome Doucettevirus
KX620751.1 Propionibacterium phage Doucette- complete genome Doucettevirus
KX620750.1 Propionibacterium phage B22- complete genome Doucettevirus
MK977715.1 Corynebacterium phage Adelaide- complete genome Samwavirus
MK977706.1 Corynebacterium phage Dina- complete genome Samwavirus
MH727560.1 Corynebacterium phage SamW- complete genome Samwavirus
MH536818.1 Gordonia phage Frokostdame- complete genome Jujuvirus
MW291016.1 Gordonia phage Blino- complete genome Jujuvirus
MT723935.1 Gordonia phage Azula- complete genome Jujuvirus
MN234161.1 Gordonia phage Toast- complete genome Fairfaxidumvirus
MK814757.1 Gordonia phage Fairfaxidum- complete genome Fairfaxidumvirus
MK801721.1 Gordonia phage William- complete genome Fairfaxidumvirus
MN103533.1 Mycobacterium phage Weirdo19- complete genome Rosariovirus
EU203571.2 Mycobacterium phage Giles- complete genome Gilesvirus
MW353181.1 Mycobacterium phage phiT46 1- complete genome Mycoabscvirus
MG770216.1 Mycobacterium phage Rem711- complete genome Trigintaduovirus
KJ028219.1 Mycobacterium phage 32HC- complete genome Trigintaduovirus
KU963261.1 Gordonia phage BetterKatz- complete genome Betterkatzvirus
MT024859.1 Gordonia phage DumpsterDude- complete genome Ruthyvirus
MH536826.1 Gordonia phage Ruthy- complete genome Ruthyvirus
KX557279.1 Gordonia phage Hedwig- complete genome Hedwigvirus
KU998235.1 Gordonia phage Bowser- complete genome Bowservirus
KU963252.2 Gordonia phage Schnabeltier- complete genome Schnabeltiervirus
KJ567043.1 Mycobacterium phage Gaia- complete genome Gaiavirus
JF937090.1 Mycobacterium phage Baka- complete genome Omegavirus
JN698997.1 Mycobacterium phage Courthouse- complete genome Omegavirus
JF937101.1 Mycobacterium phage LittleE- complete genome Omegavirus
JF937091.2 Mycobacterium phage Bask21- complete genome Kostyavirus
DQ398041.1 Mycobacterium phage 244- complete genome Kostyavirus
AY129331.1 Mycobacterium phage Cjw1- complete genome Kostyavirus
JQ809702.1 Mycobacterium phage Avani- complete genome Avanivirus Gracegardnervirinae
MF919502.1 Mycobacterium phage Demsculpinboyz- complete genome Avanivirus Gracegardnervirinae
AY129336.2 Mycobacterium phage Che9d- complete genome Avanivirus Gracegardnervirinae
KM101124.1 Mycobacterium phage Squirty- complete genome Squirtyvirus Gracegardnervirinae
MH001449.1 Mycobacterium phage MooMoo- complete genome Moomoovirus Gracegardnervirinae
MK977709.1 Mycobacterium phage ThetaBob- complete genome Thetabobvirus Gracegardnervirinae
MH651187.1 Mycobacterium phage Renaud18- complete genome Thetabobvirus Gracegardnervirinae
MH077585.1 Mycobacterium phage TChen- complete genome Thetabobvirus Gracegardnervirinae
MN908688.1 Mycobacterium phage Cornie- complete genome Corniev irus Gracegardnervirinae
EU816590.1 Mycobacterium phage Boomer- complete genome Cheoctovirus Gracegardnervirinae
GU060500.1 Mycobacterium phage Ardmore- complete genome Cheoctovirus Gracegardnervirinae
FJ174690.1 Mycobacterium phage Fruitloop- complete genome Cheoctovirus Gracegardnervirinae
KF017002.1 Mycobacterium phage Catdawg- complete genome Corndogvirus
JN698993.1 Mycobacterium phage Firecracker- complete genome Corndogvirus
AY129335.1 Mycobacterium phage Corndog- complete genome Corndogvirus
KC576783.1 Mycobacterium phage Butters- complete genome Charlievirus Nclasvirinae
MH697576.1 Mycobacterium phage Aggie- complete genome Charlievirus Nclasvirinae
MG099936.1 Mycobacterium phage Andies- complete genome Charlievirus Nclasvirinae
KP027201.1 Mycobacterium phage Sbash- complete genome Chenonavirus
AY129333.1 Mycobacterium phage Che9c- complete genome Chenonavirus
FJ168659.1 Mycobacterium phage Brujita- complete genome Brujitavirus Chebruvirinae
JN699001.1 Mycobacterium phage Babsiella- complete genome Brujitavirus Chebruvirinae
AP018487.1 Mycobacterium phage HC DNA- complete genome Brujitavirus Chebruvirinae
MH230879.1 Mycobacterium phage Xavia- complete genome Xaviavirus Pclasvirinae
KX648391.1 Mycobacterium phage Tortellini- complete genome Tortellinivirus Pclasvirinae
MN234172.1 Mycobacterium phage ThulaThula- complete genome Phayoncevirus Pclasvirinae
KR080195.1 Mycobacterium phage Phayonce- complete genome Phayoncevirus Pclasvirinae
MN096355.1 Mycobacterium phage Purky- complete genome Purkyvirus Pclasvirinae
JN412591.1 Mycobacterium phage BigNuz- complete genome Bignuzvirus Pclasvirinae
KT347313.1 Mycobacterium phage Brusacoram- complete genome Fishburnevirus Pclasvirinae
KF841477.1 Mycobacterium phage Donovan- complete genome Fishburnevirus Pclasvirinae
KC691256.1 Mycobacterium phage Fishburne- complete genome Fishburnevirus Pclasvirinae

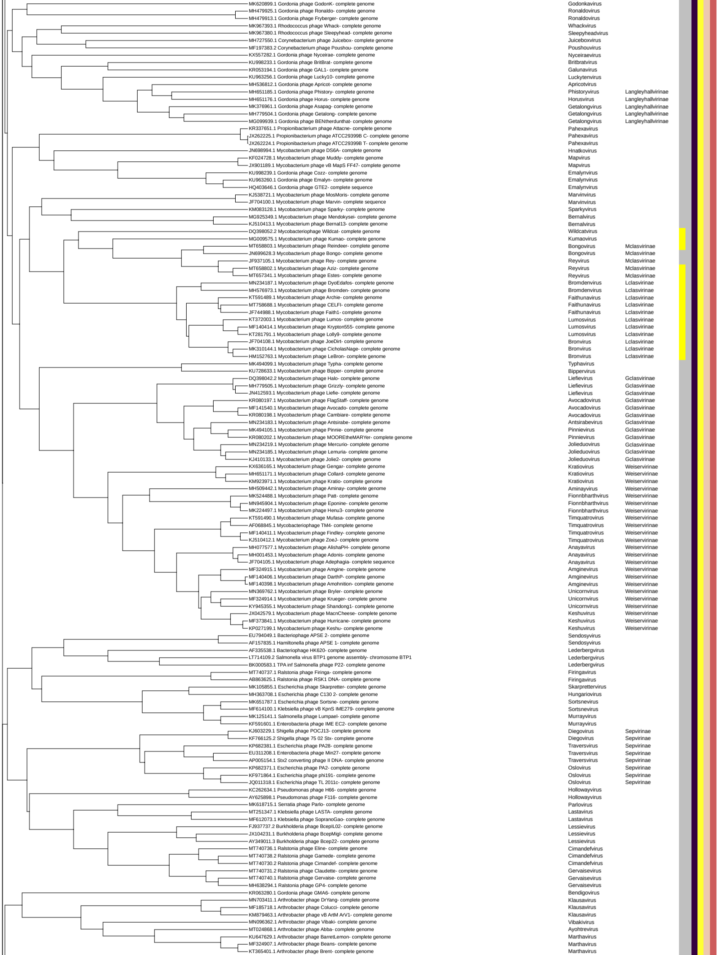
MK620899.1 Gordonia phage GodonK- complete genome Godonkavirus
MH479925.1 Gordonia phage Ronaldo- complete genome Ronaldovirus
MH479913.1 Gordonia phage Fryberger- complete genome Ronaldovirus
MK967393.1 Rhodococcus phage Whack- complete genome Whackvirus
MK967380.1 Rhodococcus phage Sleepyhead- complete genome Sleepyheadvirus
MH727550.1 Corynebacterium phage Juicebox- complete genome Juiceboxvirus
MF197383.2 Corynebacterium phage Poushou- complete genome Poushouvirus
KX557282.1 Gordonia phage Nyceirae- complete genome Nyceiraevirus
KU998233.1 Gordonia phage BritBrat- complete genome Britbratvirus
KR053194.1 Gordonia phage GAL1- complete genome Galunavirus
KU963256.1 Gordonia phage Lucky10- complete genome Luckytenvirus
MH536812.1 Gordonia phage Apricot- complete genome Apricotvirus
MH651185.1 Gordonia phage Phistory- complete genome Phistoryvirus Langleyhallvirinae
MH651176.1 Gordonia phage Horus- complete genome Horusvirus Langleyhallvirinae
MK376961.1 Gordonia phage Asapag- complete genome Getalongvirus Langleyhallvirinae
MH779504.1 Gordonia phage Getalong- complete genome Getalongvirus Langleyhallvirinae
MG099939.1 Gordonia phage BENtherdunthat- complete genome Getalongvirus Langleyhallvirinae
KR337651.1 Propionibacterium phage Attacne- complete genome Pahexavirus
JX262225.1 Propionibacterium phage ATCC29399B C- complete genome Pahexavirus
JX262224.1 Propionibacterium phage ATCC29399B T- complete genome Pahexavirus
JN698994.1 Mycobacterium phage DS6A- complete genome Hnatkovirus
KF024728.1 Mycobacterium phage Muddy- complete genome Mapvirus
JX901189.1 Mycobacterium phage vB MapS FF47- complete genome Mapvirus
KU998239.1 Gordonia phage Cozz- complete genome Emalynvirus
KU963260.1 Gordonia phage Emalyn- complete genome Emalynvirus
HQ403646.1 Gordonia phage GTE2- complete sequence Emalynvirus
KJ538721.1 Mycobacterium phage MosMoris- complete genome Marvinvirus
JF704100.1 Mycobacterium phage Marvin- complete sequence Marvinvirus
KM083128.1 Mycobacterium phage Sparky- complete genome Sparkyvirus
MG925349.1 Mycobacterium phage Mendokysei- complete genome Bernalvirus
KJ510413.1 Mycobacterium phage Bernal13- complete genome Bernalvirus
DQ398052.2 Mycobacteriophage Wildcat- complete genome Wildcatvirus
MG009575.1 Mycobacterium phage Kumao- complete genome Kumaovirus
MT658803.1 Mycobacterium phage Reindeer- complete genome Bongovirus Mclasvirinae
JN699628.3 Mycobacterium phage Bongo- complete genome Bongovirus Mclasvirinae
JF937105.1 Mycobacterium phage Rey- complete genome Reyvirus Mclasvirinae
MT658802.1 Mycobacterium phage Aziz- complete genome Reyvirus Mclasvirinae
MT657341.1 Mycobacterium phage Estes- complete genome Reyvirus Mclasvirinae
MN234187.1 Mycobacterium phage DyoEdafos- complete genome Bromdenvirus Lclasvirinae
MH576973.1 Mycobacterium phage Bromden- complete genome Bromdenvirus Lclasvirinae
KT591489.1 Mycobacterium phage Archie- complete genome Faithunavirus Lclasvirinae
MT758688.1 Mycobacterium phage CELFI- complete genome Faithunavirus Lclasvirinae
JF744988.1 Mycobacterium phage Faith1- complete genome Faithunavirus Lclasvirinae
KT372003.1 Mycobacterium phage Lumos- complete genome Lumosvirus Lclasvirinae
MF140414.1 Mycobacterium phage Krypton555- complete genome Lumosvirus Lclasvirinae
KT281791.1 Mycobacterium phage Lolly9- complete genome Lumosvirus Lclasvirinae
JF704108.1 Mycobacterium phage JoeDirt- complete genome Bronvirus Lclasvirinae
MK310144.1 Mycobacterium phage CicholasNage- complete genome Bronvirus Lclasvirinae
HM152763.1 Mycobacterium phage LeBron- complete genome Bronvirus Lclasvirinae
MK494099.1 Mycobacterium phage Typha- complete genome Typhavirus
KU728633.1 Mycobacterium phage Bipper- complete genome Bippervirus
DQ398042.2 Mycobacterium phage Halo- complete genome Liefievirus Gclasvirinae
MH779505.1 Mycobacterium phage Grizzly- complete genome Liefievirus Gclasvirinae
JN412593.1 Mycobacterium phage Liefie- complete genome Liefievirus Gclasvirinae
KR080197.1 Mycobacterium phage FlagStaff- complete genome Avocadovirus Gclasvirinae
MF141540.1 Mycobacterium phage Avocado- complete genome Avocadovirus Gclasvirinae
KR080198.1 Mycobacterium phage Cambiare- complete genome Avocadovirus Gclasvirinae
MN234183.1 Mycobacterium phage Antsirabe- complete genome Antsirabevirus Gclasvirinae
MK494105.1 Mycobacterium phage Pinnie- complete genome Pinnievirus Gclasvirinae
KR080202.1 Mycobacterium phage MOOREtheMARYer- complete genome Pinnievirus Gclasvirinae
MN234219.1 Mycobacterium phage Mercurio- complete genome Jolieduovirus Gclasvirinae
MN234185.1 Mycobacterium phage Lemuria- complete genome Jolieduovirus Gclasvirinae
KJ410133.1 Mycobacterium phage Jolie2- complete genome Jolieduovirus Gclasvirinae
KX636165.1 Mycobacterium phage Gengar- complete genome Kratiovirus Weiservirinae
MH651171.1 Mycobacterium phage Collard- complete genome Kratiovirus Weiservirinae
KM923971.1 Mycobacterium phage Kratio- complete genome Kratiovirus Weiservirinae
MH509442.1 Mycobacterium phage Aminay- complete genome Aminayvirus Weiservirinae
MK524488.1 Mycobacterium phage Patt- complete genome Fionnbharthvirus Weiservirinae
MN945904.1 Mycobacterium phage Eponine- complete genome Fionnbharthvirus Weiservirinae
MK224497.1 Mycobacterium phage Henu3- complete genome Fionnbharthvirus Weiservirinae
KT591490.1 Mycobacterium phage Mufasa- complete genome Timquatrovirus Weiservirinae
AF068845.1 Mycobacteriophage TM4- complete genome Timquatrovirus Weiservirinae
MF140411.1 Mycobacterium phage Findley- complete genome Timquatrovirus Weiservirinae
KJ510412.1 Mycobacterium phage ZoeJ- complete genome Timquatrovirus Weiservirinae
MH077577.1 Mycobacterium phage AlishaPH- complete genome Anayavirus Weiservirinae
MH001453.1 Mycobacterium phage Adonis- complete genome Anayavirus Weiservirinae
JF704105.1 Mycobacterium phage Adephagia- complete sequence Anayavirus Weiservirinae
MF324915.1 Mycobacterium phage Amgine- complete genome Amginevirus Weiservirinae
MF140406.1 Mycobacterium phage DarthP- complete genome Amginevirus Weiservirinae
MF140398.1 Mycobacterium phage Amohnition- complete genome Amginevirus Weiservirinae
MN369762.1 Mycobacterium phage Bryler- complete genome Unicornvirus Weiservirinae
MF324914.1 Mycobacterium phage Krueger- complete genome Unicornvirus Weiservirinae
KY945355.1 Mycobacterium phage Shandong1- complete genome Unicornvirus Weiservirinae
JX042579.1 Mycobacterium phage MacnCheese- complete genome Keshuvirus Weiservirinae
MF373841.1 Mycobacterium phage Hurricane- complete genome Keshuvirus Weiservirinae
KP027199.1 Mycobacterium phage Keshu- complete genome Keshuvirus Weiservirinae
EU794049.1 Bacteriophage APSE 2- complete genome Sendosyvirus
AF157835.1 Hamiltonella phage APSE 1- complete genome Sendosyvirus
AF335538.1 Bacteriophage HK620- complete genome Lederbergvirus
LT714109.2 Salmonella virus BTP1 genome assembly- chromosome BTP1 Lederbergvirus
BK000583.1 TPA inf Salmonella phage P22- complete genome Lederbergvirus
MT740737.1 Ralstonia phage Firinga- complete genome Firingavirus
AB863625.1 Ralstonia phage RSK1 DNA- complete genome Firingavirus
MK105855.1 Escherichia phage Skarpretter- complete genome Skarprettervirus
MH363708.1 Escherichia phage C130 2- complete genome Hungariovirus
MK651787.1 Escherichia phage Sortsne- complete genome Sortsnevirus
MF614100.1 Klebsiella phage vB KpnS IME279- complete genome Sortsnevirus
MK125141.1 Salmonella phage Lumpael- complete genome Murrayvirus
KF591601.1 Enterobacteria phage IME EC2- complete genome Murrayvirus
KJ603229.1 Shigella phage POCJ13- complete genome Diegovirus Sepvirinae
KF766125.2 Shigella phage 75 02 Stx- complete genome Diegovirus Sepvirinae
KP682381.1 Escherichia phage PA28- complete genome Traversvirus Sepvirinae
EU311208.1 Enterobacteria phage Min27- complete genome Traversvirus Sepvirinae
AP005154.1 Stx2 converting phage II DNA- complete genome Traversvirus Sepvirinae
KP682371.1 Escherichia phage PA2- complete genome Oslovirus Sepvirinae
KF971864.1 Escherichia phage phi191- complete genome Oslovirus Sepvirinae
JQ011318.1 Escherichia phage TL 2011c- complete genome Oslovirus Sepvirinae
KC262634.1 Pseudomonas phage H66- complete genome Hollowayvirus
AY625898.1 Pseudomonas phage F116- complete genome Hollowayvirus
MK618715.1 Serratia phage Parlo- complete genome Parlovirus
MT251347.1 Klebsiella phage LASTA- complete genome Lastavirus
MF612073.1 Klebsiella phage SopranoGao- complete genome Lastavirus
FJ937737.2 Burkholderia phage BcepIL02- complete genome Lessievirus
JX104231.1 Burkholderia phage BcepMigl- complete genome Lessievirus
AY349011.3 Burkholderia phage Bcep22- complete genome Lessievirus
MT740736.1 Ralstonia phage Eline- complete genome Cimandefvirus
MT740738.2 Ralstonia phage Gamede- complete genome Cimandefvirus
MT740730.2 Ralstonia phage Cimandef- complete genome Cimandefvirus
MT740731.2 Ralstonia phage Claudette- complete genome Gervaisevirus
MT740740.1 Ralstonia phage Gervaise- complete genome Gervaisevirus
MH638294.1 Ralstonia phage GP4- complete genome Gervaisevirus
KR063280.1 Gordonia phage GMA6- complete genome Bendigovirus
MN703411.1 Arthrobacter phage DrYang- complete genome Klausavirus
MF185718.1 Arthrobacter phage Colucci- complete genome Klausavirus
KM879463.1 Arthrobacter phage vB ArtM ArV1- complete genome Klausavirus
MN096362.1 Arthrobacter phage Vibaki- complete genome Vibakivirus
MT024868.1 Arthrobacter phage Abba- complete genome Ayohtrevirus
KU647629.1 Arthrobacter phage BarrettLemon- complete genome Marthavirus
MF324907.1 Arthrobacter phage Beans- complete genome Marthavirus
KT365401.1 Arthrobacter phage Brent- complete genome Marthavirus

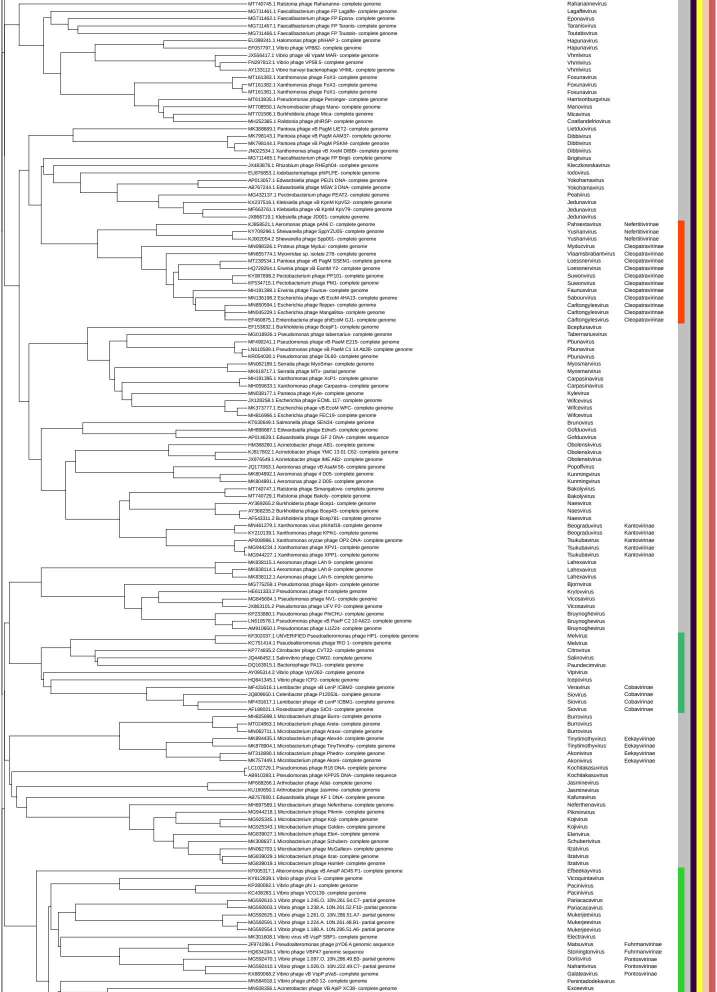
MT740745.1 Ralstonia phage Raharianne- complete genome Rahariannevirus
MG711461.1 Faecalibacterium phage FP Lagaffe- complete genome Lagaffevirus
MG711462.1 Faecalibacterium phage FP Epona- complete genome Eponavirus
MG711467.1 Faecalibacterium phage FP Taranis- complete genome Taranisvirus
MG711466.1 Faecalibacterium phage FP Toutatis- complete genome Toutatisvirus
EU399241.1 Halomonas phage phiHAP 1- complete genome Hapunavirus
EF057797.1 Vibrio phage VP882- complete genome Hapunavirus
JX556417.1 Vibrio phage vB VpaM MAR- complete genome Vhmlvirus
FN297812.1 Vibrio phage VP58.5- complete genome Vhmlvirus
AY133112.1 Vibrio harveyi bacteriophage VHML- complete genome Vhmlvirus
MT161383.1 Xanthomonas phage FoX3- complete genome Foxunavirus
MT161382.1 Xanthomonas phage FoX2- complete genome Foxunavirus
MT161381.1 Xanthomonas phage FoX1- complete genome Foxunavirus
MT613935.1 Pseudomonas phage Persinger- complete genome Harrisonburgvirus
MT708550.1 Achromobacter phage Mano- complete genome Manovirus
MT701586.1 Burkholderia phage Mica- complete genome Micavirus
MH252365.1 Ralstonia phage phiRSP- complete genome Coatlandelriovirus
MK388689.1 Pantoea phage vB PagM LIET2- complete genome Lietduovirus
MK798143.1 Pantoea phage vB PagM AAM37- complete genome Dibbivirus
MK798144.1 Pantoea phage vB PagM PSKM- complete genome Dibbivirus
JN022534.1 Xanthomonas phage vB XveM DIBBI- complete genome Dibbivirus
MG711465.1 Faecalibacterium phage FP Brigit- complete genome Brigitvirus
JX483876.1 Rhizobium phage RHEph04- complete genome Kleczkowskavirus
EU876853.1 Iodobacteriophage phiPLPE- complete genome Iodovirus
AP013057.1 Edwardsiella phage PEi21 DNA- complete genome Yokohamavirus
AB767244.1 Edwardsiella phage MSW 3 DNA- complete genome Yokohamavirus
MG432137.1 Pectinobacterium phage PEAT2- complete genome Peatvirus
KX237516.1 Klebsiella phage vB KpnM KpV52- complete genome Jedunavirus
MF663761.1 Klebsiella phage vB KpnM KpV79- complete genome Jedunavirus
JX866719.1 Klebsiella phage JD001- complete genome Jedunavirus
KJ858521.1 Aeromonas phage pAh6 C- complete genome Pahsextavirus Nefertitivirinae
KY709296.1 Shewanella phage SppYZU05- complete genome Yushanvirus Nefertitivirinae
KJ002054.2 Shewanella phage Spp001- complete genome Yushanvirus Nefertitivirinae
MN098326.1 Proteus phage Myduc- complete genome Myducvirus Cleopatravirinae
MN855774.1 Myoviridae sp. isolate 278- complete genome Vlaamsbrabantvirus Cleopatravirinae
MT230534.1 Pantoea phage vB PagM SSEM1- complete genome Loessnervirus Cleopatravirinae
HQ728264.1 Erwinia phage vB EamM Y2- complete genome Loessnervirus Cleopatravirinae
KY087898.2 Pectobacterium phage PP101- complete genome Suwonvirus Cleopatravirinae
KF534715.1 Pectobacterium phage PM1- complete genome Suwonvirus Cleopatravirinae
MH191398.1 Erwinia phage Faunus- complete genome Faunusvirus Cleopatravirinae
MN136198.2 Escherichia phage vB EcoM 4HA13- complete genome Sabourvirus Cleopatravirinae
MN850594.1 Escherichia phage flopper- complete genome Carltongylesvirus Cleopatravirinae
MN045229.1 Escherichia phage Mangalitsa- complete genome Carltongylesvirus Cleopatravirinae
EF460875.1 Enterobacteria phage phiEcoM GJ1- complete genome Carltongylesvirus Cleopatravirinae
EF153632.1 Burkholderia phage BcepF1- complete genome Bcepfunavirus
MG018926.1 Pseudomonas phage tabernarius- complete genome Tabernariusvirus
MF490241.1 Pseudomonas phage vB PaeM E215- complete genome Pbunavirus
LN610589.1 Pseudomonas phage vB PaeM C1 14 Ab28- complete genome Pbunavirus
KR054030.1 Pseudomonas phage DL60- complete genome Pbunavirus
MN062189.1 Serratia phage MyoSmar- complete genome Myosmarvirus
MK618717.1 Serratia phage MTx- partial genome Myosmarvirus
MH191395.1 Xanthomonas phage XcP1- complete genome Carpasinavirus
MH059633.1 Xanthomonas phage Carpasina- complete genome Carpasinavirus
MN038177.1 Panteoa phage Kyle- complete genome Kylevirus
JX128258.1 Escherichia phage ECML 117- complete genome Wifcevirus
MK373777.1 Escherichia phage vB EcoM WFC- complete genome Wifcevirus
MH816966.1 Escherichia phage FEC19- complete genome Wifcevirus
KT630649.1 Salmonella phage SEN34- complete genome Brunovirus
MH898687.1 Edwardsiella phage Edno5- complete genome Gofduovirus
AP014629.1 Edwardsiella phage GF 2 DNA- complete sequence Gofduovirus
HM368260.1 Acinetobacter phage AB1- complete genome Obolenskvirus
KJ817802.1 Acinetobacter phage YMC 13 01 C62- complete genome Obolenskvirus
JX976549.1 Acinetobacter phage IME AB2- complete genome Obolenskvirus
JQ177063.1 Aeromonas phage vB AsaM 56- complete genome Popoffvirus
MK804892.1 Aeromonas phage 4 D05- complete genome Kunmingvirus
MK804891.1 Aeromonas phage 2 D05- complete genome Kunmingvirus
MT740747.1 Ralstonia phage Simangalove- complete genome Bakolyvirus
MT740729.1 Ralstonia phage Bakoly- complete genome Bakolyvirus
AY369265.2 Burkholderia phage Bcep1- complete genome Naesvirus
AY368235.2 Burkholderia phage Bcep43- complete genome Naesvirus
AF543311.2 Burkholderia phage Bcep781- complete genome Naesvirus
MN461279.1 Xanthomonas virus phiXaf18- complete genome Beograduvirus Kantovirinae
KY210139.1 Xanthomonas phage KPhi1- complete genome Beograduvirus Kantovirinae
AP008986.1 Xanthomonas oryzae phage OP2 DNA- complete genome Tsukubavirus Kantovirinae
MG944234.1 Xanthomonas phage XPV1- complete genome Tsukubavirus Kantovirinae
MG944227.1 Xanthomonas phage XPP1- complete genome Tsukubavirus Kantovirinae
MK838115.1 Aeromonas phage LAh 9- complete genome Lahexavirus
MK838114.1 Aeromonas phage LAh 8- complete genome Lahexavirus
MK838112.1 Aeromonas phage LAh 6- complete genome Lahexavirus
MG775259.1 Pseudomonas phage Bjorn- complete genome Bjornvirus
HE611333.2 Pseudomonas phage tf complete genome Krylovvirus
MG845684.1 Pseudomonas phage NV1- complete genome Vicosavirus
JX863101.2 Pseudomonas phage UFV P2- complete genome Vicosavirus
KP233880.1 Pseudomonas phage PhiCHU- complete genome Bruynoghevirus
LN610578.1 Pseudomonas phage vB PaeP C2 10 Ab22- complete genome Bruynoghevirus
AM910650.1 Pseudomonas phage LUZ24- complete genome Bruynoghevirus
KF302037.1 UNVERIFIED Pseudoalteromonas phage HP1- complete genome Melvirus
KC751414.1 Pseudoalteromonas phage RIO 1- complete genome Melvirus
KP774835.2 Citrobacter phage CVT22- complete genome Citrovirus
JQ446452.1 Salinivibrio phage CW02- complete genome Salinovirus
DQ163915.1 Bacteriophage PA11- complete genome Paundecimvirus
AY095314.2 Vibrio phage VpV262- complete genome Vipivirus
HQ641345.1 Vibrio phage ICP2- complete genome Icepovirus
MF431616.1 Lentibacter phage vB LenP ICBM2- complete genome Veravirus Cobavirinae
JQ809650.1 Celeribacter phage P12053L- complete genome Siovirus Cobavirinae
MF431617.1 Lentibacter phage vB LenP ICBM1- complete genome Siovirus Cobavirinae
AF189021.1 Roseobacter phage SIO1- complete genome Siovirus Cobavirinae
MH825698.1 Microbacterium phage Burro- complete genome Burrovirus
MT024863.1 Microbacterium phage Arete- complete genome Burrovirus
MN062711.1 Microbacterium phage Araxxi- complete genome Burrovirus
MK894435.1 Microbacterium phage Alex44- complete genome Tinytimothyvirus Eekayvirinae
MK878904.1 Microbacterium phage TinyTimothy- complete genome Tinytimothyvirus Eekayvirinae
MT310890.1 Microbacterium phage Phedro- complete genome Akonivirus Eekayvirinae
MK757449.1 Microbacterium phage Akoni- complete genome Akonivirus Eekayvirinae
LC102729.1 Pseudomonas phage R18 DNA- complete genome Kochitakasuvirus
AB910393.1 Pseudomonas phage KPP25 DNA- complete sequence Kochitakasuvirus
MF668266.1 Arthrobacter phage Adat- complete genome Jasminevirus
KU160650.1 Arthrobacter phage Jasmine- complete genome Jasminevirus
AB757800.1 Edwardsiella phage KF 1 DNA- complete genome Kafunavirus
MH697589.1 Microbacterium phage Neferthena- complete genome Neferthenavirus
MG944218.1 Microbacterium phage Pikmin- complete genome Pikminvirus
MG925345.1 Microbacterium phage Koji- complete genome Kojivirus
MG925343.1 Microbacterium phage Golden- complete genome Kojivirus
MG839027.1 Microbacterium phage Eleri- complete genome Elerivirus
MK308637.1 Microbacterium phage Schubert- complete genome Schubertvirus
MN062703.1 Microbacterium phage McGalleon- complete genome Ilzatvirus
MG839029.1 Microbacterium phage Ilzat- complete genome Ilzatvirus
MG839019.1 Microbacterium phage Hamlet- complete genome Ilzatvirus
KF005317.1 Alteromonas phage vB AmaP AD45 P1- complete genome Efbeekayvirus
KY612839.1 Vibrio phage pVco 5- complete genome Vicoquintavirus
KP280062.1 Vibrio phage phi 1- complete genome Pacinivirus
KC438283.1 Vibrio phage VCO139- complete genome Pacinivirus
MG592610.1 Vibrio phage 1.245.O. 10N.261.54.C7- partial genome Pariacacavirus
MG592603.1 Vibrio phage 1.238.A. 10N.261.52.F10- partial genome Pariacacavirus
MG592625.1 Vibrio phage 1.261.O. 10N.286.51.A7- partial genome Mukerjeevirus
MG592591.1 Vibrio phage 1.224.A. 10N.261.48.B1- partial genome Mukerjeevirus
MG592554.1 Vibrio phage 1.188.A. 10N.286.51.A6- partial genome Mukerjeevirus
MK301608.1 Vibrio virus vB VspP SBP1- complete genome Electravirus
JF974296.1 Pseudoalteromonas phage pYD6 A genomic sequence Matsuvirus Fuhrmanvirinae
HQ634194.1 Vibrio phage VBP47 genomic sequence Stoningtonvirus Fuhrmanvirinae
MG592470.1 Vibrio phage 1.097.O. 10N.286.49.B3- partial genome Dorisvirus Pontosvirinae
MG592410.1 Vibrio phage 1.026.O. 10N.222.49.C7- partial genome Nahantvirus Pontosvirinae
KX889068.2 Vibrio phage vB VspP pVa5- complete genome Galateavirus Pontosvirinae
MN584918.1 Vibrio phage phi50 12- complete genome Penintadodekavirus
MN508356.1 Acinetobacter phage VB ApiP XC38- complete genome Exceevirus

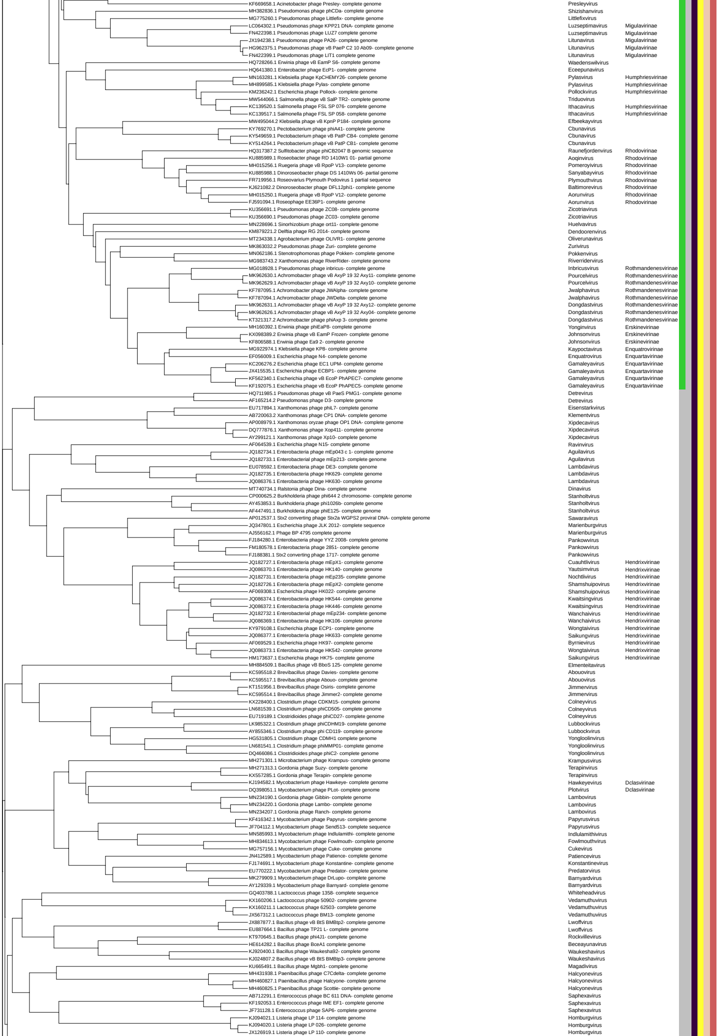
KF669658.1 Acinetobacter phage Presley- complete genome Presleyvirus
MH382836.1 Pseudomonas phage phCDa- complete genome Shizishanvirus
MG775260.1 Pseudomonas phage Littlefix- complete genome Littlefixvirus
LC064302.1 Pseudomonas phage KPP21 DNA- complete genome Luzseptimavirus Migulavirinae
FN422398.1 Pseudomonas phage LUZ7 complete genome Luzseptimavirus Migulavirinae
JX194238.1 Pseudomonas phage PA26- complete genome Litunavirus Migulavirinae
HG962375.1 Pseudomonas phage vB PaeP C2 10 Ab09- complete genome Litunavirus Migulavirinae
FN422399.1 Pseudomonas phage LIT1 complete genome Litunavirus Migulavirinae
HQ728266.1 Erwinia phage vB EamP S6- complete genome Waedenswilvirus
HQ641380.1 Enterobacter phage EcP1- complete genome Eceepunavirus
MN163281.1 Klebsiella phage KpCHEMY26- complete genome Pylasvirus Humphriesvirinae
MH899585.1 Klebsiella phage Pylas- complete genome Pylasvirus Humphriesvirinae
KM236242.1 Escherichia phage Pollock- complete genome Pollockvirus Humphriesvirinae
MW544066.1 Salmonella phage vB SalP TR2- complete genome Triduovirus
KC139520.1 Salmonella phage FSL SP 076- complete genome Ithacavirus Humphriesvirinae
KC139517.1 Salmonella phage FSL SP 058- complete genome Ithacavirus Humphriesvirinae
MW495044.2 Klebsiella phage vB KpnP P184- complete genome Efbeekayvirus
KY769270.1 Pectobacterium phage phiA41- complete genome Cbunavirus
KY549659.1 Pectobacterium phage vB PatP CB4- complete genome Cbunavirus
KY514264.1 Pectobacterium phage vB PatP CB1- complete genome Cbunavirus
HQ317387.2 Sulfitobacter phage phiCB2047 B genomic sequence Raunefjordenvirus Rhodovirinae
KU885989.1 Roseobacter phage RD 1410W1 01- partial genome Aoqinvirus Rhodovirinae
MH015256.1 Ruegeria phage vB RpoP V13- complete genome Pomeroyivirus Rhodovirinae
KU885988.1 Dinoroseobacter phage DS 1410Ws 06- partial genome Sanyabayvirus Rhodovirinae
FR719956.1 Roseovarius Plymouth Podovirus 1 partial sequence Plymouthvirus Rhodovirinae
KJ621082.2 Dinoroseobacter phage DFL12phi1- complete genome Baltimorevirus Rhodovirinae
MH015250.1 Ruegeria phage vB RpoP V12- complete genome Aorunvirus Rhodovirinae
FJ591094.1 Roseophage EE36P1- complete genome Aorunvirus Rhodovirinae
KU356691.1 Pseudomonas phage ZC08- complete genome Zicotriavirus
KU356690.1 Pseudomonas phage ZC03- complete genome Zicotriavirus
MN228696.1 Sinorhizobium phage ort11- complete genome Huelvavirus
KM879221.2 Delftia phage RG 2014- complete genome Dendoorenvirus
MT234338.1 Agrobacterium phage OLIVR1- complete genome Oliverunavirus
MK863032.2 Pseudomonas phage Zuri- complete genome Zurivirus
MN062186.1 Stenotrophomonas phage Pokken- complete genome Pokkenvirus
MG983743.2 Xanthomonas phage RiverRider- complete genome Riverridervirus
MG018928.1 Pseudomonas phage inbricus- complete genome Inbricusvirus Rothmandenesvirinae
MK962630.1 Achromobacter phage vB AxyP 19 32 Axy11- complete genome Pourcelvirus Rothmandenesvirinae
MK962629.1 Achromobacter phage vB AxyP 19 32 Axy10- complete genome Pourcelvirus Rothmandenesvirinae
KF787095.1 Achromobacter phage JWAlpha- complete genome Jwalphavirus Rothmandenesvirinae
KF787094.1 Achromobacter phage JWDelta- complete genome Jwalphavirus Rothmandenesvirinae
MK962631.1 Achromobacter phage vB AxyP 19 32 Axy12- complete genome Dongdastvirus Rothmandenesvirinae
MK962626.1 Achromobacter phage vB AxyP 19 32 Axy04- complete genome Dongdastvirus Rothmandenesvirinae
KT321317.2 Achromobacter phage phiAxp 3- complete genome Dongdastvirus Rothmandenesvirinae
MH160392.1 Erwinia phage phiEaP8- complete genome Yonginvirus Erskinevirinae
KX098389.2 Erwinia phage vB EamP Frozen- complete genome Johnsonvirus Erskinevirinae
KF806588.1 Erwinia phage Ea9 2- complete genome Johnsonvirus Erskinevirinae
MG922974.1 Klebsiella phage KP8- complete genome Kaypoctavirus Enquatrovirinae
EF056009.1 Escherichia phage N4- complete genome Enquatrovirus Enquartavirinae
KC206276.2 Escherichia phage EC1 UPM- complete genome Gamaleyavirus Enquartavirinae
JX415535.1 Escherichia phage ECBP1- complete genome Gamaleyavirus Enquartavirinae
KF562340.1 Escherichia phage vB EcoP PhAPEC7- complete genome Gamaleyavirus Enquartavirinae
KF192075.1 Escherichia phage vB EcoP PhAPEC5- complete genome Gamaleyavirus Enquartavirinae
HQ711985.1 Pseudomonas phage vB PaeS PMG1- complete genome Detrevirus
AF165214.2 Pseudomonas phage D3- complete genome Detrevirus
EU717894.1 Xanthomonas phage phiL7- complete genome Eisenstarkvirus
AB720063.2 Xanthomonas phage CP1 DNA- complete genome Klementvirus
AP008979.1 Xanthomonas oryzae phage OP1 DNA- complete genome Xipdecavirus
DQ777876.1 Xanthomonas phage Xop411- complete genome Xipdecavirus
AY299121.1 Xanthomonas phage Xp10- complete genome Xipdecavirus
AF064539.1 Escherichia phage N15- complete genome Ravinvirus
JQ182734.1 Enterobacteria phage mEp043 c 1- complete genome Aguilavirus
JQ182733.1 Enterobacterial phage mEp213- complete genome Aguilavirus
EU078592.1 Enterobacteria phage DE3- complete genome Lambdavirus
JQ182735.1 Enterobacteria phage HK629- complete genome Lambdavirus
JQ086376.1 Enterobacteria phage HK630- complete genome Lambdavirus
MT740734.1 Ralstonia phage Dina- complete genome Dinavirus
CP000625.2 Burkholderia phage phi644 2 chromosome- complete genome Stanholtvirus
AY453853.1 Burkholderia phage phi1026b- complete genome Stanholtvirus
AF447491.1 Burkholderia phage phiE125- complete genome Stanholtvirus
AP012537.1 Stx2 converting phage Stx2a WGPS2 proviral DNA- complete genome Sawaravirus
JQ347801.1 Escherichia phage JLK 2012- complete sequence Marienburgvirus
AJ556162.1 Phage BP 4795 complete genome Marienburgvirus
FJ184280.1 Enterobacteria phage YYZ 2008- complete genome Pankowvirus
FM180578.1 Enterobacteria phage 2851- complete genome Pankowvirus
FJ188381.1 Stx2 converting phage 1717- complete genome Pankowvirus
JQ182727.1 Enterobacteria phage mEpX1- complete genome Cuauhtlivirus Hendrixvirinae
JQ086370.1 Enterobacteria phage HK140- complete genome Yautsimvirus Hendrixvirinae
JQ182731.1 Enterobacteria phage mEp235- complete genome Nochtlivirus Hendrixvirinae
JQ182726.1 Enterobacteria phage mEpX2- complete genome Shamshuipovirus Hendrixvirinae
AF069308.1 Escherichia phage HK022- complete genome Shamshuipovirus Hendrixvirinae
JQ086374.1 Enterobacteria phage HK544- complete genome Kwaitsingvirus Hendrixvirinae
JQ086372.1 Enterobacteria phage HK446- complete genome Kwaitsingvirus Hendrixvirinae
JQ182732.1 Enterobacterial phage mEp234- complete genome Wanchaivirus Hendrixvirinae
JQ086369.1 Enterobacteria phage HK106- complete genome Wanchaivirus Hendrixvirinae
KY979108.1 Escherichia phage ECP1- complete genome Wongtaivirus Hendrixvirinae
JQ086377.1 Enterobacteria phage HK633- complete genome Saikungvirus Hendrixvirinae
AF069529.1 Escherichia phage HK97- complete genome Byrnievirus Hendrixvirinae
JQ086373.1 Enterobacteria phage HK542- complete genome Wongtaivirus Hendrixvirinae
HM173637.1 Escherichia phage HK75- complete genome Saikungvirus Hendrixvirinae
MH884509.1 Bacillus phage vB BboS 125- complete genome Elmenteitavirus
KC595518.2 Brevibacillus phage Davies- complete genome Abouovirus
KC595517.1 Brevibacillus phage Abouo- complete genome Abouovirus
KT151956.1 Brevibacillus phage Osiris- complete genome Jimmervirus
KC595514.1 Brevibacillus phage Jimmer2- complete genome Jimmervirus
KX228400.1 Clostridium phage CDKM15- complete genome Colneyvirus
LN681539.1 Clostridium phage phiCD505- complete genome Colneyvirus
EU719189.1 Clostridioides phage phiCD27- complete genome Colneyvirus
LK985322.1 Clostridium phage phiCDHM19- complete genome Lubbockvirus
AY855346.1 Clostridium phage phi CD119- complete genome Lubbockvirus
HG531805.1 Clostridium phage CDMH1 complete genome Yongloolinvirus
LN681541.1 Clostridium phage phiMMP01- complete genome Yongloolinvirus
DQ466086.1 Clostridioides phage phiC2- complete genome Yongloolinvirus
MH271301.1 Microbacterium phage Krampus- complete genome Krampusvirus
MH271313.1 Gordonia phage Suzy- complete genome Terapinvirus
KX557285.1 Gordonia phage Terapin- complete genome Terapinvirus
KJ194582.1 Mycobacterium phage Hawkeye- complete genome Hawkeyevirus Dclasvirinae
DQ398051.1 Mycobacterium phage PLot- complete genome Plotvirus Dclasvirinae
MN234190.1 Gordonia phage Gibbin- complete genome Lambovirus
MN234220.1 Gordonia phage Lambo- complete genome Lambovirus
MN234207.1 Gordonia phage Ranch- complete genome Lambovirus
KF416342.1 Mycobacterium phage Papyrus- complete genome Papyrusvirus
JF704112.1 Mycobacterium phage Send513- complete sequence Papyrusvirus
MN585993.1 Mycobacterium phage Indlulamithi- complete genome Indlulamithivirus
MH834613.1 Mycobacterium phage Fowlmouth- complete genome Fowlmouthvirus
MG757156.1 Mycobacterium phage Cuke- complete genome Cukevirus
JN412589.1 Mycobacterium phage Patience- complete genome Patiencevirus
FJ174691.1 Mycobacterium phage Konstantine- complete genome Konstantinevirus
EU770222.1 Mycobacterium phage Predator- complete genome Predatorvirus
MK279909.1 Mycobacterium phage DrLupo- complete genome Barnyardvirus
AY129339.1 Mycobacterium phage Barnyard- complete genome Barnyardvirus
GQ403788.1 Lactococcus phage 1358- complete sequence Whiteheadvirus
KX160206.1 Lactococcus phage 50902- complete genome Vedamuthuvirus
KX160211.1 Lactococcus phage 62503- complete genome Vedamuthuvirus
JX567312.1 Lactococcus phage BM13- complete genome Vedamuthuvirus
JX887877.1 Bacillus phage vB BtS BMBtp2- complete genome Lwoffvirus
EU887664.1 Bacillus phage TP21 L- complete genome Lwoffvirus
KT970645.1 Bacillus phage phi4J1- complete genome Rockvillevirus
HE614282.1 Bacillus phage BceA1 complete genome Beceayunavirus
KJ920400.1 Bacillus phage Waukesha92- complete genome Waukeshavirus
KJ024807.2 Bacillus phage vB BtS BMBtp3- complete genome Waukeshavirus
KU665491.1 Bacillus phage Mgbh1- complete genome Magadivirus
MH431938.1 Paenibacillus phage C7Cdelta- complete genome Halcyonevirus
MH460827.1 Paenibacillus phage Halcyone- complete genome Halcyonevirus
MH460825.1 Paenibacillus phage Scottie- complete genome Halcyonevirus
AB712291.1 Enterococcus phage BC 611 DNA- complete genome Saphexavirus
KF192053.1 Enterococcus phage IME EF1- complete genome Saphexavirus
JF731128.1 Enterococcus phage SAP6- complete genome Saphexavirus
KJ094021.1 Listeria phage LP 114- complete genome Homburgvirus
KJ094020.1 Listeria phage LP 026- complete genome Homburgvirus
JX126919.1 Listeria phage LP 110- complete genome Homburgvirus

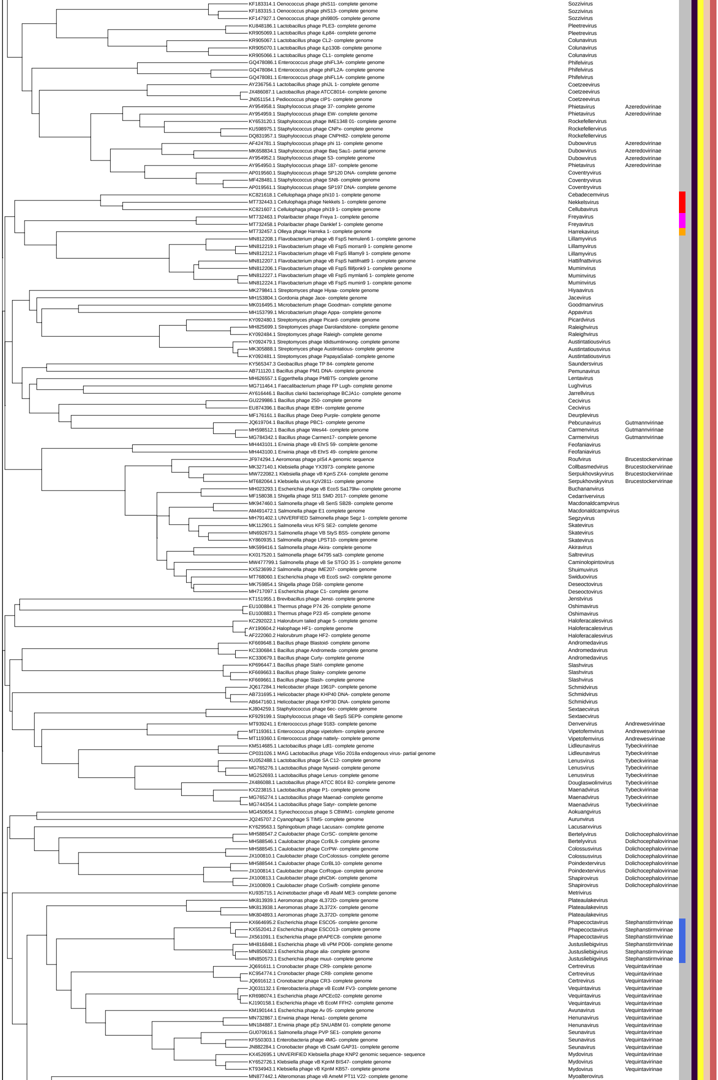
KF183314.1 Oenococcus phage phiS11- complete genome Sozzivirus
KF183315.1 Oenococcus phage phiS13- complete genome Sozzivirus
KF147927.1 Oenococcus phage phi9805- complete genome Sozzivirus
KU848186.1 Lactobacillus phage PLE3- complete genome Pleetrevirus
KR905069.1 Lactobacillus phage iLp84- complete genome Pleetrevirus
KR905067.1 Lactobacillus phage CL2- complete genome Colunavirus
KR905070.1 Lactobacillus phage iLp1308- complete genome Colunavirus
KR905066.1 Lactobacillus phage CL1- complete genome Colunavirus
GQ478086.1 Enterococcus phage phiFL3A- complete genome Phifelvirus
GQ478084.1 Enterococcus phage phiFL2A- complete genome Phifelvirus
GQ478081.1 Enterococcus phage phiFL1A- complete genome Phifelvirus
AY236756.1 Lactobacillus phage phiJL 1- complete genome Coetzeevirus
JX486087.1 Lactobacillus phage ATCC8014- complete genome Coetzeevirus
JN051154.1 Pediococcus phage clP1- complete genome Coetzeevirus
AY954958.1 Staphylococcus phage 37- complete genome Phietavirus Azeredovirinae
AY954959.1 Staphylococcus phage EW- complete genome Phietavirus Azeredovirinae
KY653120.1 Staphylococcus phage IME1348 01- complete genome Rockefellervirus
KU598975.1 Staphylococcus phage CNPx- complete genome Rockefellervirus
DQ831957.1 Staphylococcus phage CNPH82- complete genome Rockefellervirus
AF424781.1 Staphylococcus phage phi 11- complete genome Dubowvirus Azeredovirinae
MK658834.1 Staphylococcus phage Baq Sau1- partial genome Dubowvirus Azeredovirinae
AY954952.1 Staphylococcus phage 53- complete genome Dubowvirus Azeredovirinae
AY954950.1 Staphylococcus phage 187- complete genome Phietavirus Azeredovirinae
AP019560.1 Staphylococcus phage SP120 DNA- complete genome Coventryvirus
MF428481.1 Staphylococcus phage SN8- complete genome Coventryvirus
AP019561.1 Staphylococcus phage SP197 DNA- complete genome Coventryvirus
KC821618.1 Cellulophaga phage phi10 1- complete genome Cebadecemvirus
MT732443.1 Cellulophaga phage Nekkels 1- complete genome Nekkelsvirus
KC821607.1 Cellulophaga phage phi19 1- complete genome Cellubavirus
MT732463.1 Polaribacter phage Freya 1- complete genome Freyavirus
MT732458.1 Polaribacter phage Danklef 1- complete genome Freyavirus
MT732457.1 Olleya phage Harreka 1- complete genome Harrekavirus
MN812208.1 Flavobacterium phage vB FspS hemulen6 1- complete genome Lillamyvirus
MN812219.1 Flavobacterium phage vB FspS morran9 1- complete genome Lillamyvirus
MN812212.1 Flavobacterium phage vB FspS lillamy9 1- complete genome Lillamyvirus
MN812207.1 Flavobacterium phage vB FspS hattifnatt9 1- complete genome Hattifnattvirus
MN812206.1 Flavobacterium phage vB FspS filifjonk9 1- complete genome Muminvirus
MN812227.1 Flavobacterium phage vB FspS mymlan6 1- complete genome Muminvirus
MN812224.1 Flavobacterium phage vB FspS mumin9 1- complete genome Muminvirus
MK279841.1 Streptomyces phage Hiyaa- complete genome Hiyaavirus
MH153804.1 Gordonia phage Jace- complete genome Jacevirus
MK016495.1 Microbacterium phage Goodman- complete genome Goodmanvirus
MH153799.1 Microbacterium phage Appa- complete genome Appavirus
KY092480.1 Streptomyces phage Picard- complete genome Picardvirus
MH825699.1 Streptomyces phage Darolandstone- complete genome Raleighvirus
KY092484.1 Streptomyces phage Raleigh- complete genome Raleighvirus
KY092479.1 Streptomyces phage Ididsumtinwong- complete genome Austintatiousvirus
MK305888.1 Streptomyces phage Austintatious- complete genome Austintatiousvirus
KY092481.1 Streptomyces phage PapayaSalad- complete genome Austintatiousvirus
KY565347.3 Geobacillus phage TP 84- complete genome Saundersvirus
AB711120.1 Bacillus phage PM1 DNA- complete genome Pemunavirus
MH626557.1 Eggerthella phage PMBT5- complete genome Lentavirus
MG711464.1 Faecalibacterium phage FP Lugh- complete genome Lughvirus
AY616446.1 Bacillus clarkii bacteriophage BCJA1c- complete genome Jarrellvirus
GU229986.1 Bacillus phage 250- complete genome Cecivirus
EU874396.1 Bacillus phage IEBH- complete genome Cecivirus
MF176161.1 Bacillus phage Deep Purple- complete genome Deurplevirus
JQ619704.1 Bacillus phage PBC1- complete genome Pebcunavirus Gutmannvirinae
MH598512.1 Bacillus phage Wes44- complete genome Carmenvirus Gutmannvirinae
MG784342.1 Bacillus phage Carmen17- complete genome Carmenvirus Gutmannvirinae
MH443101.1 Erwinia phage vB EhrS 59- complete genome Feofaniavirus
MH443100.1 Erwinia phage vB EhrS 49- complete genome Feofaniavirus
JF974294.1 Aeromonas phage pIS4 A genomic sequence Roufvirus Brucestockervirinae
MK327140.1 Klebsiella phage YX3973- complete genome Collbasmedvirus Brucestockervirinae
MW722082.1 Klebsiella phage vB KpnS ZX4- complete genome Serpukhovskyvirus Brucestockervirinae
MT682064.1 Klebsiella virus KpV2811- complete genome Serpukhovskyvirus Brucestockervirinae
MH023293.1 Escherichia phage vB EcoS Sa179lw- complete genome Buchananvirus
MF158038.1 Shigella phage Sf11 SMD 2017- complete genome Cedarrivervirus
MK947460.1 Salmonella phage vB SenS SB28- complete genome Macdonaldcampvirus
AM491472.1 Salmonella phage E1 complete genome Macdonaldcampvirus
MH791402.1 UNVERIFIED Salmonella phage Segz 1- complete genome Segzyvirus
MK112901.1 Salmonella virus KFS SE2- complete genome Skatevirus
MN692673.1 Salmonella phage VB StyS BS5- complete genome Skatevirus
KY860935.1 Salmonella phage LPST10- complete genome Skatevirus
MK599416.1 Salmonella phage Akira- complete genome Akiravirus
KX017520.1 Salmonella phage 64795 sal3- complete genome Saltrevirus
MW477799.1 Salmonella phage vB Se STGO 35 1- complete genome Caminolopintovirus
KX523699.2 Salmonella phage IME207- complete genome Shuimuvirus
MT768060.1 Escherichia phage vB EcoS swi2- complete genome Swiduovirus
MK759854.1 Shigella phage DS8- complete genome Deseoctovirus
MH717097.1 Escherichia phage C1- complete genome Deseoctovirus
KT151955.1 Brevibacillus phage Jenst- complete genome Jenstvirus
EU100884.1 Thermus phage P74 26- complete genome Oshimavirus
EU100883.1 Thermus phage P23 45- complete genome Oshimavirus
KC292022.1 Halorubrum tailed phage 5- complete genome Haloferacalesvirus
AY190604.2 Halophage HF1- complete genome Haloferacalesvirus
AF222060.2 Halorubrum phage HF2- complete genome Haloferacalesvirus
KF669648.1 Bacillus phage Blastoid- complete genome Andromedavirus
KC330684.1 Bacillus phage Andromeda- complete genome Andromedavirus
KC330679.1 Bacillus phage Curly- complete genome Andromedavirus
KP696447.1 Bacillus phage Stahl- complete genome Slashvirus
KF669663.1 Bacillus phage Staley- complete genome Slashvirus
KF669661.1 Bacillus phage Slash- complete genome Slashvirus
JQ617284.1 Helicobacter phage 1961P- complete genome Schmidvirus
AB731695.1 Helicobacter phage KHP40 DNA- complete genome Schmidvirus
AB647160.1 Helicobacter phage KHP30 DNA- complete genome Schmidvirus
KJ804259.1 Staphylococcus phage 6ec- complete genome Sextaecvirus
KF929199.1 Staphylococcus phage vB SepS SEP9- complete genome Sextaecvirus
MT939241.1 Enterococcus phage 9183- complete genome Denvervirus Andrewesvirinae
MT119361.1 Enterocucus phage vipetofem- complete genome Vipetofemvirus Andrewesvirinae
MT119360.1 Enterococcus phage nattely- complete genome Vipetofemvirus Andrewesvirinae
KM514685.1 Lactobacillus phage Ldl1- complete genome Lidleunavirus Tybeckvirinae
CP031026.1 MAG Lactobacillus phage ViSo 2018a endogenous virus- partial genome Lidleunavirus Tybeckvirinae
KU052488.1 Lactobacillus phage SA C12- complete genome Lenusvirus Tybeckvirinae
MG765276.1 Lactobacillus phage Nyseid- complete genome Lenusvirus Tybeckvirinae
MG252693.1 Lactobacillus phage Lenus- complete genome Lenusvirus Tybeckvirinae
JX486088.1 Lactobacillus phage ATCC 8014 B2- complete genome Douglaswolinvirus Tybeckvirinae
KX223815.1 Lactobacillus phage P1- complete genome Maenadvirus Tybeckvirinae
MG765274.1 Lactobacillus phage Maenad- complete genome Maenadvirus Tybeckvirinae
MG744354.1 Lactobacillus phage Satyr- complete genome Maenadvirus Tybeckvirinae
MG450654.1 Synechococcus phage S CBWM1- complete genome Aokuangvirus
JQ245707.2 Cyanophage S TIM5- complete genome Aurunvirus
KY629563.1 Sphingobium phage Lacusarx- complete genome Lacusarxvirus
MH588547.2 Caulobacter phage CcrSC- complete genome Bertelyvirus Dolichocephalovirinae
MH588546.1 Caulobacter phage CcrBL9- complete genome Bertelyvirus Dolichocephalovirinae
MH588545.1 Caulobacter phage CcrPW- complete genome Colossusvirus Dolichocephalovirinae
JX100810.1 Caulobacter phage CcrColossus- complete genome Colossusvirus Dolichocephalovirinae
MH588544.1 Caulobacter phage CcrBL10- complete genome Poindextervirus Dolichocephalovirinae
JX100814.1 Caulobacter phage CcrRogue- complete genome Poindextervirus Dolichocephalovirinae
JX100813.1 Caulobacter phage phiCbK- complete genome Shapirovirus Dolichocephalovirinae
JX100809.1 Caulobacter phage CcrSwift- complete genome Shapirovirus Dolichocephalovirinae
KU935715.1 Acinetobacter phage vB AbaM ME3- complete genome Metrivirus
MK813939.1 Aeromonas phage 4L372D- complete genome Plateaulakevirus
MK813938.1 Aeromonas phage 2L372X- complete genome Plateaulakevirus
MK804893.1 Aeromonas phage 2L372D- complete genome Plateaulakevirus
KX664695.2 Escherichia phage ESCO5- complete genome Phapecoctavirus Stephanstirmvirinae
KX552041.2 Escherichia phage ESCO13- complete genome Phapecoctavirus Stephanstirmvirinae
JX561091.1 Escherichia phage phAPEC8- complete genome Phapecoctavirus Stephanstirmvirinae
MH816848.1 Escherichia phage vB vPM PD06- complete genome Justusliebigvirus Stephanstirmvirinae
MN850632.1 Escherichia phage alia- complete genome Justusliebigvirus Stephanstirmvirinae
MN850573.1 Escherichia phage muut- complete genome Justusliebigvirus Stephanstirmvirinae
JQ691611.1 Cronobacter phage CR9- complete genome Certrevirus Vequintavirinae
KC954774.1 Cronobacter phage CR8- complete genome Certrevirus Vequintavirinae
JQ691612.1 Cronobacter phage CR3- complete genome Certrevirus Vequintavirinae
JQ031132.1 Enterobacteria phage vB EcoM FV3- complete genome Vequintavirus Vequintavirinae
KR698074.1 Escherichia phage APCEc02- complete genome Vequintavirus Vequintavirinae
KJ190158.1 Escherichia phage vB EcoM FFH2- complete genome Vequintavirus Vequintavirinae
KM190144.1 Escherichia phage Av 05- complete genome Avunavirus Vequintavirinae
MN732867.1 Erwinia phage Hena1- complete genome Henunavirus Vequintavirinae
MN184887.1 Erwinia phage pEp SNUABM 01- complete genome Henunavirus Vequintavirinae
GU070616.1 Salmonella phage PVP SE1- complete genome Seunavirus Vequintavirinae
KF550303.1 Enterobacteria phage 4MG- complete genome Seunavirus Vequintavirinae
JN882284.1 Cronobacter phage vB CsaM GAP31- complete genome Seunavirus Vequintavirinae
KX452695.1 UNVERIFIED Klebsiella phage KNP2 genomic sequence- sequence Mydovirus Vequintavirinae
KY652726.1 Klebsiella phage vB KpnM BIS47- complete genome Mydovirus Vequintavirinae
KT934943.1 Klebsiella phage vB KpnM KB57- complete genome Mydovirus Vequintavirinae
MN877442.1 Alteromonas phage vB AmeM PT11 V22- complete genome Myoalterovirus

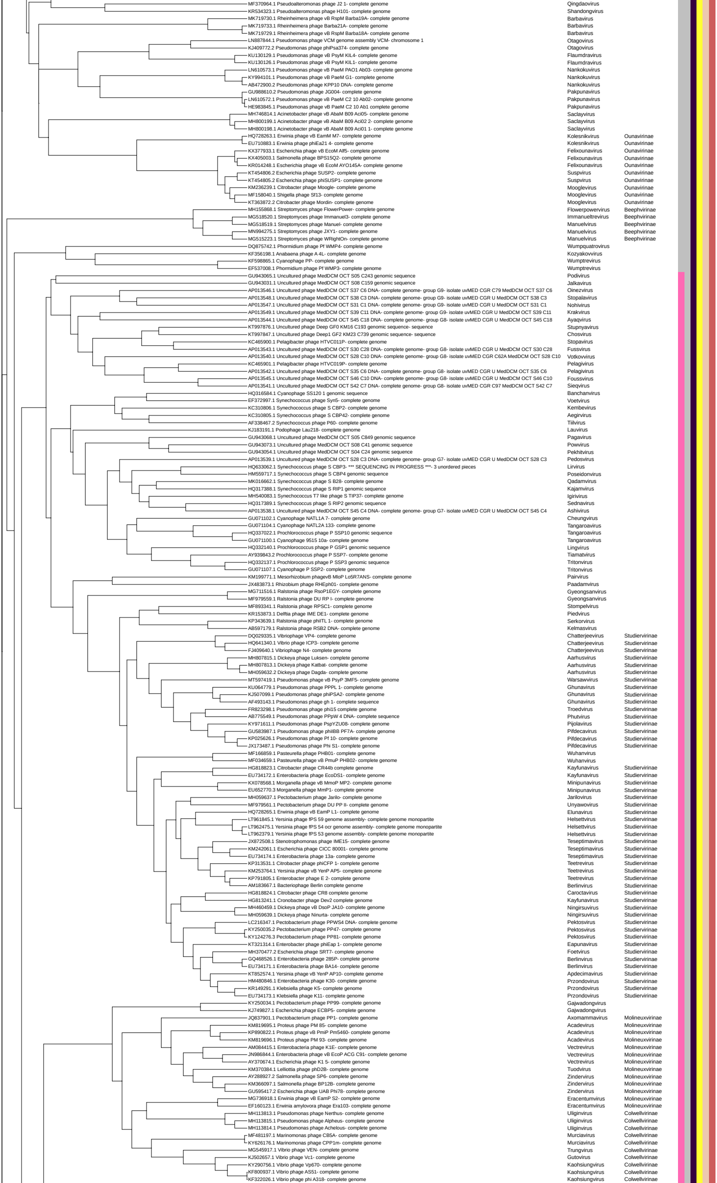

MF370964.1 Pseudoalteromonas phage J2 1- complete genome Qingdaovirus
KR534323.1 Pseudoalteromonas phage H101- complete genome Shandongvirus
MK719730.1 Rheinheimera phage vB RspM Barba19A- complete genome Barbavirus
MK719733.1 Rheinheimera phage Barba21A- complete genome Barbavirus
MK719729.1 Rheinheimera phage vB RspM Barba18A- complete genome Barbavirus
LN887844.1 Pseudomonas phage VCM genome assembly VCM- chromosome 1 Otagovirus
KJ409772.2 Pseudomonas phage phiPsa374- complete genome Otagovirus
KU130129.1 Pseudomonas phage vB PsyM KIL4- complete genome Flaumdravirus
KU130126.1 Pseudomonas phage vB PsyM KIL1- complete genome Flaumdravirus
LN610573.1 Pseudomonas phage vB PaeM PAO1 Ab03- complete genome Nankokuvirus
KY994101.1 Pseudomonas phage vB PaeM G1- complete genome Nankokuvirus
AB472900.2 Pseudomonas phage KPP10 DNA- complete genome Nankokuvirus
GU988610.2 Pseudomonas phage JG004- complete genome Pakpunavirus
LN610572.1 Pseudomonas phage vB PaeM C2 10 Ab02- complete genome Pakpunavirus
HE983845.1 Pseudomonas phage vB PaeM C2 10 Ab1 complete genome Pakpunavirus
MH746814.1 Acinetobacter phage vB AbaM B09 Aci05- complete genome Saclayvirus
MH800199.1 Acinetobacter phage vB AbaM B09 Aci02 2- complete genome Saclayvirus
MH800198.1 Acinetobacter phage vB AbaM B09 Aci01 1- complete genome Saclayvirus
HQ728263.1 Erwinia phage vB EamM M7- complete genome Kolesnikvirus Ounavirinae
EU710883.1 Erwinia phage phiEa21 4- complete genome Kolesnikvirus Ounavirinae
KX377933.1 Escherichia phage vB EcoM Alf5- complete genome Felixounavirus Ounavirinae
KX405003.1 Salmonella phage BPS15Q2- complete genome Felixounavirus Ounavirinae
KR014248.1 Escherichia phage vB EcoM AYO145A- complete genome Felixounavirus Ounavirinae
KT454806.2 Escherichia phage SUSP2- complete genome Suspvirus Ounavirinae
KT454805.2 Escherichia phage phiSUSP1- complete genome Suspvirus Ounavirinae
KM236239.1 Citrobacter phage Moogle- complete genome Mooglevirus Ounavirinae
MF158040.1 Shigella phage Sf13- complete genome Mooglevirus Ounavirinae
KT363872.2 Citrobacter phage Mordin- complete genome Mooglevirus Ounavirinae
MH155868.1 Streptomyces phage FlowerPower- complete genome Flowerpowervirus Beephvirinae
MG518520.1 Streptomyces phage Immanuel3- complete genome Immanueltrevirus Beephvirinae
MG518519.1 Streptomyces phage Manuel- complete genome Manuelvirus Beephvirinae
MN994275.1 Streptomyces phage JXY1- complete genome Manuelvirus Beephvirinae
MG515223.1 Streptomyces phage WRightOn- complete genome Manuelvirus Beephvirinae
DQ875742.1 Phormidium phage Pf WMP4- complete genome Wumpquatrovirus
KF356198.1 Anabaena phage A 4L- complete genome Kozyakovvirus
KF598865.1 Cyanophage PP- complete genome Wumptrevirus
EF537008.1 Phormidium phage Pf WMP3- complete genome Wumptrevirus
GU943065.1 Uncultured phage MedDCM OCT S05 C243 genomic sequence Podivirus
GU943031.1 Uncultured phage MedDCM OCT S08 C159 genomic sequence Jalkavirus
AP013546.1 Uncultured phage MedDCM OCT S37 C6 DNA- complete genome- group G9- isolate uvMED CGR C79 MedDCM OCT S37 C6 Oinezvirus
AP013548.1 Uncultured phage MedDCM OCT S38 C3 DNA- complete genome- group G9- isolate uvMED CGR U MedDCM OCT S38 C3 Stopalavirus
AP013547.1 Uncultured phage MedDCM OCT S31 C1 DNA- complete genome- group G9- isolate uvMED CGR U MedDCM OCT S31 C1 Nohivirus
AP013549.1 Uncultured phage MedDCM OCT S39 C11 DNA- complete genome- group G9- isolate uvMED CGR U MedDCM OCT S39 C11 Krakvirus
AP013544.1 Uncultured phage MedDCM OCT S45 C18 DNA- complete genome- group G8- isolate uvMED CGR U MedDCM OCT S45 C18 Ayaqvirus
KT997876.1 Uncultured phage Deep GF0 KM16 C193 genomic sequence- sequence Stupnyavirus
KT997847.1 Uncultured phage Deep1 GF2 KM23 C739 genomic sequence- sequence Chosvirus
KC465900.1 Pelagibacter phage HTVC011P- complete genome Stopavirus
AP013543.1 Uncultured phage MedDCM OCT S30 C28 DNA- complete genome- group G8- isolate uvMED CGR U MedDCM OCT S30 C28 Fussvirus
AP013540.1 Uncultured phage MedDCM OCT S28 C10 DNA- complete genome- group G8- isolate uvMED CGR C62A MedDCM OCT S28 C10 Votkovvirus
KC465901.1 Pelagibacter phage HTVC019P- complete genome Pelagivirus
AP013542.1 Uncultured phage MedDCM OCT S35 C6 DNA- complete genome- group G8- isolate uvMED CGR U MedDCM OCT S35 C6 Pelagivirus
AP013545.1 Uncultured phage MedDCM OCT S46 C10 DNA- complete genome- group G8- isolate uvMED CGR U MedDCM OCT S46 C10 Foussvirus
AP013541.1 Uncultured phage MedDCM OCT S42 C7 DNA- complete genome- group G8- isolate uvMED CGR C97 MedDCM OCT S42 C7 Sieqvirus
HQ316584.1 Cyanophage SS120 1 genomic sequence Banchanvirus
EF372997.1 Synechococcus phage Syn5- complete genome Voetvirus
KC310806.1 Synechococcus phage S CBP2- complete genome Kembevirus
KC310805.1 Synechococcus phage S CBP42- complete genome Aegirvirus
AF338467.2 Synechococcus phage P60- complete genome Tiilvirus
KJ183191.1 Podophage Lau218- complete genome Lauvirus
GU943068.1 Uncultured phage MedDCM OCT S05 C849 genomic sequence Pagavirus
GU943073.1 Uncultured phage MedDCM OCT S08 C41 genomic sequence Powvirus
GU943054.1 Uncultured phage MedDCM OCT S04 C24 genomic sequence Pekhitvirus
AP013539.1 Uncultured phage MedDCM OCT S28 C3 DNA- complete genome- group G7- isolate uvMED CGR U MedDCM OCT S28 C3 Pedosvirus
HQ633062.1 Synechococcus phage S CBP3- *** SEQUENCING IN PROGRESS ***- 3 unordered pieces Lirvirus
HM559717.1 Synechococcus phage S CBP4 genomic sequence Poseidonvirus
MK016662.1 Synechococcus phage S B28- complete genome Qadamvirus
HQ317388.1 Synechococcus phage S RIP1 genomic sequence Kajamvirus
MH540083.1 Synechococcus T7 like phage S TIP37- complete genome Igirivirus
HQ317389.1 Synechococcus phage S RIP2 genomic sequence Sednavirus
AP013538.1 Uncultured phage MedDCM OCT S45 C4 DNA- complete genome- group G7- isolate uvMED CGR U MedDCM OCT S45 C4 Ashivirus
GU071102.1 Cyanophage NATL1A 7- complete genome Cheungvirus
GU071104.1 Cyanophage NATL2A 133- complete genome Tangaroavirus
HQ337022.1 Prochlorococcus phage P SSP10 genomic sequence Tangaroavirus
GU071100.1 Cyanophage 9515 10a- complete genome Tangaroavirus
HQ332140.1 Prochlorococcus phage P GSP1 genomic sequence Lingvirus
AY939843.2 Prochlorococcus phage P SSP7- complete genome Tiamatvirus
HQ332137.1 Prochlorococcus phage P SSP3 genomic sequence Tritonvirus
GU071107.1 Cyanophage P SSP2- complete genome Tritonvirus
KM199771.1 Mesorhizobium phagevB MloP Lo5R7ANS- complete genome Pairvirus
JX483873.1 Rhizobium phage RHEph01- complete genome Paadamvirus
MG711516.1 Ralstonia phage RsoP1EGY- complete genome Gyeongsanvirus
MF979559.1 Ralstonia phage DU RP I- complete genome Gyeongsanvirus
MF893341.1 Ralstonia phage RPSC1- complete genome Stompelvirus
KR153873.1 Delftia phage IME DE1- complete genome Piedvirus
KP343639.1 Ralstonia phage phiITL 1- complete genome Serkorvirus
AB597179.1 Ralstonia phage RSB2 DNA- complete genome Kelmasvirus
DQ029335.1 Vibriophage VP4- complete genome Chatterjeevirus Studiervirinae
HQ641340.1 Vibrio phage ICP3- complete genome Chatterjeevirus Studiervirinae
FJ409640.1 Vibriophage N4- complete genome Chatterjeevirus Studiervirinae
MH807815.1 Dickeya phage Luksen- complete genome Aarhusvirus Studiervirinae
MH807813.1 Dickeya phage Katbat- complete genome Aarhusvirus Studiervirinae
MH059632.2 Dickeya phage Dagda- complete genome Aarhusvirus Studiervirinae
MT597419.1 Pseudomonas phage vB PsyP 3MF5- complete genome Warsawvirus Studiervirinae
KU064779.1 Pseudomonas phage PPPL 1- complete genome Ghunavirus Studiervirinae
KJ507099.1 Pseudomonas phage phiPSA2- complete genome Ghunavirus Studiervirinae
AF493143.1 Pseudomonas phage gh 1- complete sequence Ghunavirus Studiervirinae
FR823298.1 Pseudomonas phage phi15 complete genome Troedvirus Studiervirinae
AB775549.1 Pseudomonas phage PPpW 4 DNA- complete sequence Phutvirus Studiervirinae
KY971611.1 Pseudomonas phage PspYZU08- complete genome Pijolavirus Studiervirinae
GU583987.1 Pseudomonas phage phiIBB PF7A- complete genome Pifdecavirus Studiervirinae
KP025626.1 Pseudomonas phage Pf 10- complete genome Pifdecavirus Studiervirinae
JX173487.1 Pseudomonas phage Phi S1- complete genome Pifdecavirus Studiervirinae
MF166859.1 Pasteurella phage PHB01- complete genome Wuhanvirus
MF034659.1 Pasteurella phage vB PmuP PHB02- complete genome Wuhanvirus
HG818823.1 Citrobacter phage CR44b complete genome Kayfunavirus Studiervirinae
EU734172.1 Enterobacteria phage EcoDS1- complete genome Kayfunavirus Studiervirinae
KX078568.1 Morganella phage vB MmoP MP2- complete genome Minipunavirus Studiervirinae
EU652770.3 Morganella phage MmP1- complete genome Minipunavirus Studiervirinae
MH059637.1 Pectobacterium phage Jarilo- complete genome Jarilovirus Studiervirinae
MF979561.1 Pectobacterium phage DU PP II- complete genome Unyawovirus Studiervirinae
HQ728265.1 Erwinia phage vB EamP L1- complete genome Elunavirus Studiervirinae
LT961845.1 Yersinia phage fPS 59 genome assembly- complete genome monopartite Helsettvirus Studiervirinae
LT962475.1 Yersinia phage fPS 54 ocr genome assembly- complete genome monopartite Helsettvirus Studiervirinae
LT962379.1 Yersinia phage fPS 53 genome assembly- complete genome monopartite Helsettvirus Studiervirinae
JX872508.1 Stenotrophomonas phage IME15- complete genome Teseptimavirus Studiervirinae
KM242061.1 Escherichia phage CICC 80001- complete genome Teseptimavirus Studiervirinae
EU734174.1 Enterobacteria phage 13a- complete genome Teseptimavirus Studiervirinae
KP313531.1 Citrobacter phage phiCFP 1- complete genome Teetrevirus Studiervirinae
KM253764.1 Yersinia phage vB YenP AP5- complete genome Teetrevirus Studiervirinae
KP791805.1 Enterobacter phage E 2- complete genome Teetrevirus Studiervirinae
AM183667.1 Bacteriophage Berlin complete genome Berlinvirus Studiervirinae
HG818824.1 Citrobacter phage CR8 complete genome Caroctavirus Studiervirinae
HG813241.1 Cronobacter phage Dev2 complete genome Kayfunavirus Studiervirinae
MH460459.1 Dickeya phage vB DsoP JA10- complete genome Ningirsuvirus Studiervirinae
MH059639.1 Dickeya phage Ninurta- complete genome Ningirsuvirus Studiervirinae
LC216347.1 Pectobacterium phage PPWS4 DNA- complete genome Pektosvirus Studiervirinae
KY250035.2 Pectobacterium phage PP47- complete genome Pektosvirus Studiervirinae
KY124276.3 Pectobacterium phage PP81- complete genome Pektosvirus Studiervirinae
KT321314.1 Enterobacter phage phiEap 1- complete genome Eapunavirus Studiervirinae
MH370477.2 Escherichia phage SRT7- complete genome Foetvirus Studiervirinae
GQ468526.1 Enterobacteria phage 285P- complete genome Berlinvirus Studiervirinae
EU734171.1 Enterobacteria phage BA14- complete genome Berlinvirus Studiervirinae
KT852574.1 Yersinia phage vB YenP AP10- complete genome Apdecimavirus Studiervirinae
HM480846.1 Enterobacteria phage K30- complete genome Przondovirus Studiervirinae
KR149291.1 Klebsiella phage K5- complete genome Przondovirus Studiervirinae
EU734173.1 Klebsiella phage K11- complete genome Przondovirus Studiervirinae
KY250034.1 Pectobacterium phage PP99- complete genome Gajwadongvirus
KJ749827.1 Escherichia phage ECBP5- complete genome Gajwadongvirus
JQ837901.1 Pectobacterium phage PP1- complete genome Axomammavirus Molineuxvirinae
KM819695.1 Proteus phage PM 85- complete genome Acadevirus Molineuxvirinae
KP890822.1 Proteus phage vB PmiP Pm5460- complete genome Acadevirus Molineuxvirinae
KM819696.1 Proteus phage PM 93- complete genome Acadevirus Molineuxvirinae
AM084415.1 Enterobacteria phage K1E- complete genome Vectrevirus Molineuxvirinae
JN986844.1 Enterobacteria phage vB EcoP ACG C91- complete genome Vectrevirus Molineuxvirinae
AY370674.1 Escherichia phage K1 5- complete genome Vectrevirus Molineuxvirinae
KM370384.1 Lelliottia phage phD2B- complete genome Tuodvirus Molineuxvirinae
AY288927.2 Salmonella phage SP6- complete genome Zindervirus Molineuxvirinae
KM366097.1 Salmonella phage BP12B- complete genome Zindervirus Molineuxvirinae
GU595417.2 Escherichia phage UAB Phi78- complete genome Zindervirus Molineuxvirinae
MG736918.1 Erwinia phage vB EamP S2- complete genome Eracentumvirus Molineuxvirinae
EF160123.1 Erwinia amylovora phage Era103- complete genome Eracentumvirus Molineuxvirinae
MH113813.1 Pseudomonas phage Nerthus- complete genome Uliginvirus Colwellvirinae
MH113815.1 Pseudomonas phage Alpheus- complete genome Uliginvirus Colwellvirinae
MH113814.1 Pseudomonas phage Achelous- complete genome Uliginvirus Colwellvirinae
MF481197.1 Marinomonas phage CB5A- complete genome Murciavirus Colwellvirinae
KY626176.1 Marinomonas phage CPP1m- complete genome Murciavirus Colwellvirinae
MG545917.1 Vibrio phage VEN- complete genome Trungvirus Colwellvirinae
KJ502657.1 Vibrio phage Vc1- complete genome Gutovirus Colwellvirinae
KY290756.1 Vibrio phage Vp670- complete genome Kaohsiungvirus Colwellvirinae
KF800937.1 Vibrio phage AS51- complete genome Kaohsiungvirus Colwellvirinae
KF322026.1 Vibrio phage phi A318- complete genome Kaohsiungvirus Colwellvirinae

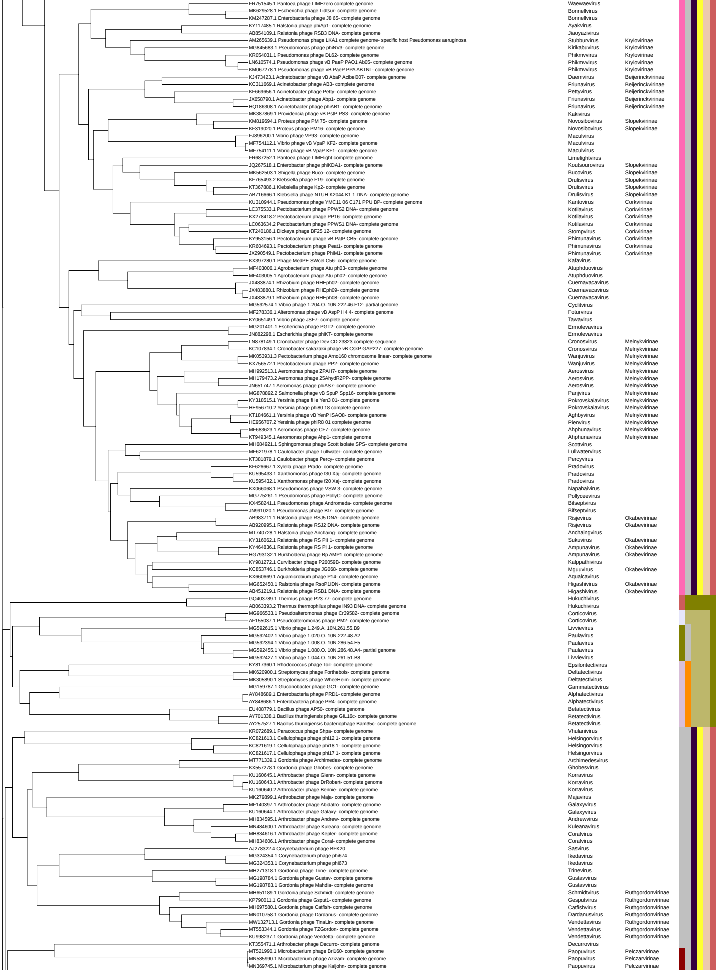

FR751545.1 Pantoea phage LIMEzero complete genome Waewaevirus
MK629528.1 Escherichia phage Lidtsur- complete genome Bonnellvirus
KM247287.1 Enterobacteria phage J8 65- complete genome Bonnellvirus
KY117485.1 Ralstonia phage phiAp1- complete genome Ayakvirus
AB854109.1 Ralstonia phage RSB3 DNA- complete genome Jiaoyazivirus
AM265639.1 Pseudomonas phage LKA1 complete genome- specific host Pseudomonas aeruginosa Stubburvirus Krylovirinae
MG845683.1 Pseudomonas phage phiNV3- complete genome Kirikabuvirus Krylovirinae
KR054031.1 Pseudomonas phage DL62- complete genome Phikmvvirus Krylovirinae
LN610574.1 Pseudomonas phage vB PaeP PAO1 Ab05- complete genome Phikmvvirus Krylovirinae
KM067278.1 Pseudomonas phage vB PaeP PPA ABTNL- complete genome Phikmvvirus Krylovirinae
KJ473423.1 Acinetobacter phage vB AbaP Acibel007- complete genome Daemvirus Beijerinckvirinae
KC311669.1 Acinetobacter phage AB3- complete genome Friunavirus Beijerinckvirinae
KF669656.1 Acinetobacter phage Petty- complete genome Pettyvirus Beijerinckvirinae
JX658790.1 Acinetobacter phage Abp1- complete genome Friunavirus Beijerinckvirinae
HQ186308.1 Acinetobacter phage phiAB1- complete genome Friunavirus Beijerinckvirinae
MK387869.1 Providencia phage vB PstP PS3- complete genome Kakivirus
KM819694.1 Proteus phage PM 75- complete genome Novosibovirus Slopekvirinae
KF319020.1 Proteus phage PM16- complete genome Novosibovirus Slopekvirinae
FJ896200.1 Vibrio phage VP93- complete genome Maculvirus
MF754112.1 Vibrio phage vB VpaP KF2- complete genome Maculvirus
MF754111.1 Vibrio phage vB VpaP KF1- complete genome Maculvirus
FR687252.1 Pantoea phage LIMElight complete genome Limelightvirus
JQ267518.1 Enterobacter phage phiKDA1- complete genome Koutsourovirus Slopekvirinae
MK562503.1 Shigella phage Buco- complete genome Bucovirus Slopekvirinae
KF765493.2 Klebsiella phage F19- complete genome Drulisvirus Slopekvirinae
KT367886.1 Klebsiella phage Kp2- complete genome Drulisvirus Slopekvirinae
AB716666.1 Klebsiella phage NTUH K2044 K1 1 DNA- complete genome Drulisvirus Slopekvirinae
KU310944.1 Pseudomonas phage YMC11 06 C171 PPU BP- complete genome Kantovirus Corkvirinae
LC375533.1 Pectobacterium phage PPWS2 DNA- complete genome Kotilavirus Corkvirinae
KX278418.2 Pectobacterium phage PP16- complete genome Kotilavirus Corkvirinae
LC063634.2 Pectobacterium phage PPWS1 DNA- complete genome Kotilavirus Corkvirinae
KT240186.1 Dickeya phage BF25 12- complete genome Stompvirus Corkvirinae
KY953156.1 Pectobacterium phage vB PatP CB5- complete genome Phimunavirus Corkvirinae
KR604693.1 Pectobacterium phage Peat1- complete genome Phimunavirus Corkvirinae
JX290549.1 Pectobacterium phage PhiM1- complete genome Phimunavirus Corkvirinae
KX397280.1 Phage MedPE SWcel C56- complete genome Kafavirus
MF403006.1 Agrobacterium phage Atu ph03- complete genome Atuphduovirus
MF403005.1 Agrobacterium phage Atu ph02- complete genome Atuphduovirus
JX483874.1 Rhizobium phage RHEph02- complete genome Cuernavacavirus
JX483880.1 Rhizobium phage RHEph09- complete genome Cuernavacavirus
JX483879.1 Rhizobium phage RHEph08- complete genome Cuernavacavirus
MG592574.1 Vibrio phage 1.204.O. 10N.222.46.F12- partial genome Cyclitvirus
MF278336.1 Alteromonas phage vB AspP H4 4- complete genome Foturvirus
KY065149.1 Vibrio phage JSF7- complete genome Tawavirus
MG201401.1 Escherichia phage PGT2- complete genome Ermolevavirus
JN882298.1 Escherichia phage phiKT- complete genome Ermolevavirus
LN878149.1 Cronobacter phage Dev CD 23823 complete sequence Cronosvirus Melnykvirinae
KC107834.1 Cronobacter sakazakii phage vB CskP GAP227- complete genome Cronosvirus Melnykvirinae
MK053931.3 Pectobacterium phage Arno160 chromosome linear- complete genome Wanjuvirus Melnykvirinae
KX756572.1 Pectobacterium phage PP2- complete genome Wanjuvirus Melnykvirinae
MH992513.1 Aeromonas phage ZPAH7- complete genome Aerosvirus Melnykvirinae
MH179473.2 Aeromonas phage 25AhydR2PP- complete genome Aerosvirus Melnykvirinae
JN651747.1 Aeromonas phage phiAS7- complete genome Aerosvirus Melnykvirinae
MG878892.2 Salmonella phage vB SpuP Spp16- complete genome Panjvirus Melnykvirinae
KY318515.1 Yersinia phage fHe Yen3 01- complete genome Pokrovskaiavirus Melnykvirinae
HE956710.2 Yersinia phage phi80 18 complete genome Pokrovskaiavirus Melnykvirinae
KT184661.1 Yersinia phage vB YenP ISAO8- complete genome Aghbyvirus Melnykvirinae
HE956707.2 Yersinia phage phiR8 01 complete genome Pienvirus Melnykvirinae
MF683623.1 Aeromonas phage CF7- complete genome Ahphunavirus Melnykvirinae
KT949345.1 Aeromonas phage Ahp1- complete genome Ahphunavirus Melnykvirinae
MH684921.1 Sphingomonas phage Scott isolate SPS- complete genome Scottvirus
MF621978.1 Caulobacter phage Lullwater- complete genome Lullwatervirus
KT381879.1 Caulobacter phage Percy- complete genome Percyvirus
KF626667.1 Xylella phage Prado- complete genome Pradovirus
KU595433.1 Xanthomonas phage f30 Xaj- complete genome Pradovirus
KU595432.1 Xanthomonas phage f20 Xaj- complete genome Pradovirus
KX066068.1 Pseudomonas phage VSW 3- complete genome Napahaivirus
MG775261.1 Pseudomonas phage PollyC- complete genome Pollyceevirus
KX458241.1 Pseudomonas phage Andromeda- complete genome Bifseptvirus
JN991020.1 Pseudomonas phage Bf7- complete genome Bifseptvirus
AB983711.1 Ralstonia phage RSJ5 DNA- complete genome Risjevirus Okabevirinae
AB920995.1 Ralstonia phage RSJ2 DNA- complete genome Risjevirus Okabevirinae
MT740728.1 Ralstonia phage Anchaing- complete genome Anchaingvirus
KY316062.1 Ralstonia phage RS PII 1- complete genome Sukuvirus Okabevirinae
KY464836.1 Ralstonia phage RS PI 1- complete genome Ampunavirus Okabevirinae
HG793132.1 Burkholderia phage Bp AMP1 complete genome Ampunavirus Okabevirinae
KY981272.1 Curvibacter phage P26059B- complete genome Kalppathivirus
KC853746.1 Burkholderia phage JG068- complete genome Mguuvirus Okabevirinae
KX660669.1 Aquamicrobium phage P14- complete genome Aqualcavirus
MG652450.1 Ralstonia phage RsoP1IDN- complete genome Higashivirus Okabevirinae
AB451219.1 Ralstonia phage RSB1 DNA- complete genome Higashivirus Okabevirinae
GQ403789.1 Thermus phage P23 77- complete genome Hukuchivirus
AB063393.2 Thermus thermophilus phage IN93 DNA- complete genome Hukuchivirus
MG966533.1 Pseudoalteromonas phage Cr39582- complete genome Corticovirus
AF155037.1 Pseudoalteromonas phage PM2- complete genome Corticovirus
MG592615.1 Vibrio phage 1.249.A. 10N.261.55.B9 Livvievirus
MG592402.1 Vibrio phage 1.020.O. 10N.222.48.A2 Paulavirus
MG592394.1 Vibrio phage 1.008.O. 10N.286.54.E5 Paulavirus
MG592455.1 Vibrio phage 1.080.O. 10N.286.48.A4- partial genome Paulavirus
MG592427.1 Vibrio phage 1.044.O. 10N.261.51.B8 Livvievirus
KY817360.1 Rhodococcus phage Toil- complete genome Epsilontectivirus
MK620900.1 Streptomyces phage Forthebois- complete genome Deltatectivirus
MK305890.1 Streptomyces phage WheeHeim- complete genome Deltatectivirus
MG159787.1 Gluconobacter phage GC1- complete genome Gammatectivirus
AY848689.1 Enterobacteria phage PRD1- complete genome Alphatectivirus
AY848686.1 Enterobacteria phage PR4- complete genome Alphatectivirus
EU408779.1 Bacillus phage AP50- complete genome Betatectivirus
AY701338.1 Bacillus thuringiensis phage GIL16c- complete genome Betatectivirus
AY257527.1 Bacillus thuringiensis bacteriophage Bam35c- complete genome Betatectivirus
KR072689.1 Paracoccus phage Shpa- complete genome Vhulanivirus
KC821613.1 Cellulophaga phage phi12 1- complete genome Helsingorvirus
KC821619.1 Cellulophaga phage phi18 1- complete genome Helsingorvirus
KC821617.1 Cellulophaga phage phi17 1- complete genome Helsingorvirus
MT771339.1 Gordonia phage Archimedes- complete genome Archimedesvirus
KX557278.1 Gordonia phage Ghobes- complete genome Ghobesvirus
KU160645.1 Arthrobacter phage Glenn- complete genome Korravirus
KU160643.1 Arthrobacter phage DrRobert- complete genome Korravirus
KU160640.2 Arthrobacter phage Bennie- complete genome Korravirus
MK279899.1 Arthrobacter phage Maja- complete genome Majavirus
MF140397.1 Arthrobacter phage Abidatro- complete genome Galaxyvirus
KU160644.1 Arthrobacter phage Galaxy- complete genome Galaxyvirus
MH834595.1 Arthrobacter phage Andrew- complete genome Andrewvirus
MN484600.1 Arthrobacter phage Kuleana- complete genome Kuleanavirus
MH834616.1 Arthrobacter phage Kepler- complete genome Coralvirus
MH834606.1 Arthrobacter phage Coral- complete genome Coralvirus
AJ278322.4 Corynebacterium phage BFK20 Sasvirus
MG324354.1 Corynebacterium phage phi674 Ikedavirus
MG324353.1 Corynebacterium phage phi673 Ikedavirus
MH271318.1 Gordonia phage Trine- complete genome Trinevirus
MG198784.1 Gordonia phage Gustav- complete genome Gustavvirus
MG198783.1 Gordonia phage Mahdia- complete genome Gustavvirus
MH651189.1 Gordonia phage Schmidt- complete genome Schmidtvirus Ruthgordonvirinae
KP790011.1 Gordonia phage Gsput1- complete genome Gesputvirus Ruthgordonvirinae
MH697580.1 Gordonia phage Catfish- complete genome Catfishvirus Ruthgordonvirinae
MN010758.1 Gordonia phage Dardanus- complete genome Dardanusvirus Ruthgordonvirinae
MW132713.1 Gordonia phage TinaLin- complete genome Vendettavirus Ruthgordonvirinae
MT553344.1 Gordonia phage TZGordon- complete genome Vendettavirus Ruthgordonvirinae
KU998237.1 Gordonia phage Vendetta- complete genome Vendettavirus Ruthgordonvirinae
KT355471.1 Arthrobacter phage Decurro- complete genome Decurrovirus
MT521990.1 Microbacterium phage Bri160- complete genome Paopuvirus Pelczarvirinae
MN585990.1 Microbacterium phage Azizam- complete genome Paopuvirus Pelczarvirinae
MN369745.1 Microbacterium phage Kaijohn- complete genome Paopuvirus Pelczarvirinae

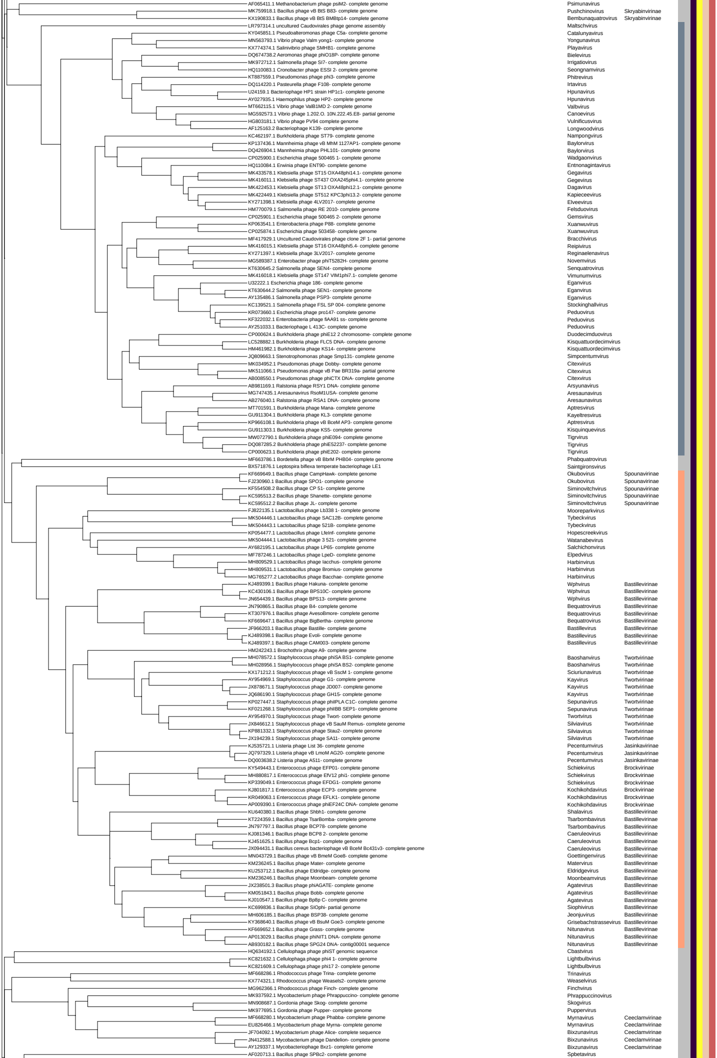
AF065411.1 Methanobacterium phage psiM2- complete genome Psimunavirus
MK759918.1 Bacillus phage vB BtS B83- complete genome Pushchinovirus Skryabinvirinae
KX190833.1 Bacillus phage vB BtS BMBtp14- complete genome Bembunaquatrovirus Skryabinvirinae
LR797314.1 uncultured Caudovirales phage genome assembly Maltschvirus
KY045851.1 Pseudoalteromonas phage C5a- complete genome Catalunyavirus
MN563793.1 Vibrio phage Valm yong1- complete genome Yongunavirus
KX774374.1 Salinivibrio phage SMHB1- complete genome Playavirus
DQ674738.2 Aeromonas phage phiO18P- complete genome Bielevirus
MK972712.1 Salmonella phage SI7- complete genome Irrigatiovirus
HQ110083.1 Cronobacter phage ESSI 2- complete genome Seongnamvirus
KT887559.1 Pseudomonas phage phi3- complete genome Phitrevirus
DQ114220.1 Pasteurella phage F108- complete genome Irtavirus
U24159.1 Bacteriophage HP1 strain HP1c1- complete genome Hpunavirus
AY027935.1 Haemophilus phage HP2- complete genome Hpunavirus
MT662115.1 Vibrio phage ValB1MD 2- complete genome Valbvirus
MG592573.1 Vibrio phage 1.202.O. 10N.222.45.E8- partial genome Canoevirus
HG803181.1 Vibrio phage PV94 complete genome Vulnificusvirus
AF125163.2 Bacteriophage K139- complete genome Longwoodvirus
KC462197.1 Burkholderia phage ST79- complete genome Nampongvirus
KP137436.1 Mannheimia phage vB MhM 1127AP1- complete genome Baylorvirus
DQ426904.1 Mannheimia phage PHL101- complete genome Baylorvirus
CP025900.1 Escherichia phage 500465 1- complete genome Wadgaonvirus
HQ110084.1 Erwinia phage ENT90- complete genome Entnonagintavirus
MK433578.1 Klebsiella phage ST15 OXA48phi14.1- complete genome Gegavirus
MK416011.1 Klebsiella phage ST437 OXA245phi4.1- complete genome Gegevirus
MK422453.1 Klebsiella phage ST13 OXA48phi12.1- complete genome Dagavirus
MK422449.1 Klebsiella phage ST512 KPC3phi13.2- complete genome Kapieceevirus
KY271398.1 Klebsiella phage 4LV2017- complete genome Elveevirus
HM770079.1 Salmonella phage RE 2010- complete genome Felsduovirus
CP025901.1 Escherichia phage 500465 2- complete genome Gemsvirus
KP063541.1 Enterobacteria phage P88- complete genome Xuanwuvirus
CP025874.1 Escherichia phage 503458- complete genome Xuanwuvirus
MF417929.1 Uncultured Caudovirales phage clone 2F 1- partial genome Bracchivirus
MK416015.1 Klebsiella phage ST16 OXA48phi5.4- complete genome Reipivirus
KY271397.1 Klebsiella phage 3LV2017- complete genome Reginaelenavirus
MG589387.1 Enterobacter phage phiT5282H- complete genome Novemvirus
KT630645.2 Salmonella phage SEN4- complete genome Senquatrovirus
MK416018.1 Klebsiella phage ST147 VIM1phi7.1- complete genome Vimunumvirus
U32222.1 Escherichia phage 186- complete genome Eganvirus
KT630644.2 Salmonella phage SEN1- complete genome Eganvirus
AY135486.1 Salmonella phage PSP3- complete genome Eganvirus
KC139521.1 Salmonella phage FSL SP 004- complete genome Stockinghallvirus
KR073660.1 Escherichia phage pro147- complete genome Peduovirus
KF322032.1 Enterobacteria phage fiAA91 ss- complete genome Peduovirus
AY251033.1 Bacteriophage L 413C- complete genome Peduovirus
CP000624.1 Burkholderia phage phiE12 2 chromosome- complete genome Duodecimduovirus
LC528882.1 Burkholderia phage FLC5 DNA- complete genome Kisquattuordecimvirus
HM461982.1 Burkholderia phage KS14- complete genome Kisquattuordecimvirus
JQ809663.1 Stenotrophomonas phage Smp131- complete genome Simpcentumvirus
MK034952.1 Pseudomonas phage Dobby- complete genome Citexvirus
MK511066.1 Pseudomonas phage vB Pae BR319a- partial genome Citexvirus
AB008550.1 Pseudomonas phage phiCTX DNA- complete genome Citexvirus
AB981169.1 Ralstonia phage RSY1 DNA- complete genome Arsyunavirus
MG747435.1 Aresaunavirus RsoM1USA- complete genome Aresaunavirus
AB276040.1 Ralstonia phage RSA1 DNA- complete genome Aresaunavirus
MT701591.1 Burkholderia phage Mana- complete genome Aptresvirus
GU911304.1 Burkholderia phage KL3- complete genome Kayeltresvirus
KP966108.1 Burkholderia phage vB BceM AP3- complete genome Aptresvirus
GU911303.1 Burkholderia phage KS5- complete genome Kisquinquevirus
MW072790.1 Burkholderia phage phiE094- complete genome Tigrvirus
DQ087285.2 Burkholderia phage phiE52237- complete genome Tigrvirus
CP000623.1 Burkholderia phage phiE202- complete genome Tigrvirus
MF663786.1 Bordetella phage vB BbrM PHB04- complete genome Phabquatrovirus
BX571876.1 Leptospira biflexa temperate bacteriophage LE1 Saintgironsvirus
KF669649.1 Bacillus phage CampHawk- complete genome Okubovirus Spounavirinae
FJ230960.1 Bacillus phage SPO1- complete genome Okubovirus Spounavirinae
KF554508.2 Bacillus phage CP 51- complete genome Siminovitchvirus Spounavirinae
KC595513.2 Bacillus phage Shanette- complete genome Siminovitchvirus Spounavirinae
KC595512.2 Bacillus phage JL- complete genome Siminovitchvirus Spounavirinae
FJ822135.1 Lactobacillus phage Lb338 1- complete genome Mooreparkvirus
MK504446.1 Lactobacillus phage SAC12B- complete genome Tybeckvirus
MK504443.1 Lactobacillus phage 521B- complete genome Tybeckvirus
KP054477.1 Lactobacillus phage LfeInf- complete genome Hopescreekvirus
MK504444.1 Lactobacillus phage 3 521- complete genome Watanabevirus
AY682195.1 Lactobacillus phage LP65- complete genome Salchichonvirus
MF787246.1 Lactobacillus phage LpeD- complete genome Elpedvirus
MH809529.1 Lactobacillus phage Iacchus- complete genome Harbinvirus
MH809531.1 Lactobacillus phage Bromius- complete genome Harbinvirus
MG765277.2 Lactobacillus phage Bacchae- complete genome Harbinvirus
KJ489399.1 Bacillus phage Hakuna- complete genome Wphvirus Bastillevirinae
KC430106.1 Bacillus phage BPS10C- complete genome Wphvirus Bastillevirinae
JN654439.1 Bacillus phage BPS13- complete genome Wphvirus Bastillevirinae
JN790865.1 Bacillus phage B4- complete genome Bequatrovirus Bastillevirinae
KT307976.1 Bacillus phage AvesoBmore- complete genome Bequatrovirus Bastillevirinae
KF669647.1 Bacillus phage BigBertha- complete genome Bequatrovirus Bastillevirinae
JF966203.1 Bacillus phage Bastille- complete genome Bastillevirus Bastillevirinae
KJ489398.1 Bacillus phage Evoli- complete genome Bastillevirus Bastillevirinae
KJ489397.1 Bacillus phage CAM003- complete genome Bastillevirus Bastillevirinae
HM242243.1 Brochothrix phage A9- complete genome
MH078572.1 Staphylococcus phage phiSA BS1- complete genome Baoshanvirus Twortvirinae
MH028956.1 Staphylococcus phage phiSA BS2- complete genome Baoshanvirus Twortvirinae
KX171212.1 Staphylococcus phage vB SscM 1- complete genome Sciuriunavirus Twortvirinae
AY954969.1 Staphylococcus phage G1- complete genome Kayvirus Twortvirinae
JX878671.1 Staphylococcus phage JD007- complete genome Kayvirus Twortvirinae
JQ686190.1 Staphylococcus phage GH15- complete genome Kayvirus Twortvirinae
KP027447.1 Staphylococcus phage phiIPLA C1C- complete genome Sepunavirus Twortvirinae
KF021268.1 Staphylococcus phage phiIBB SEP1- complete genome Sepunavirus Twortvirinae
AY954970.1 Staphylococcus phage Twort- complete genome Twortvirus Twortvirinae
JX846612.1 Staphylococcus phage vB SauM Remus- complete genome Silviavirus Twortvirinae
KP881332.1 Staphylococcus phage Stau2- complete genome Silviavirus Twortvirinae
JX194239.1 Staphylococcus phage SA11- complete genome Silviavirus Twortvirinae
KJ535721.1 Listeria phage List 36- complete genome Pecentumvirus Jasinkavirinae
JQ797329.1 Listeria phage vB LmoM AG20- complete genome Pecentumvirus Jasinkavirinae
DQ003638.2 Listeria phage A511- complete genome Pecentumvirus Jasinkavirinae
KY549443.1 Enterococcus phage EFP01- complete genome Schiekvirus Brockvirinae
MH880817.1 Enterococcus phage EfV12 phi1- complete genome Schiekvirus Brockvirinae
KP339049.1 Enterococcus phage EFDG1- complete genome Schiekvirus Brockvirinae
KJ801817.1 Enterococcus phage ECP3- complete genome Kochikohdavirus Brockvirinae
KR049063.1 Enterococcus phage EFLK1- complete genome Kochikohdavirus Brockvirinae
AP009390.1 Enterococcus phage phiEF24C DNA- complete genome Kochikohdavirus Brockvirinae
KU640380.1 Bacillus phage Shbh1- complete genome Shalavirus Bastillevirinae
KT224359.1 Bacillus phage TsarBomba- complete genome Tsarbombavirus Bastillevirinae
JN797797.1 Bacillus phage BCP78- complete genome Tsarbombavirus Bastillevirinae
KJ081346.1 Bacillus phage BCP8 2- complete genome Caeruleovirus Bastillevirinae
KJ451625.1 Bacillus phage Bcp1- complete genome Caeruleovirus Bastillevirinae
JX094431.1 Bacillus cereus bacteriophage vB BceM Bc431v3- complete genome Caeruleovirus Bastillevirinae
MN043729.1 Bacillus phage vB BmeM Goe8- complete genome Goettingenvirus Bastillevirinae
KM236245.1 Bacillus phage Mater- complete genome Matervirus Bastillevirinae
KU253712.1 Bacillus phage Eldridge- complete genome Eldridgevirus Bastillevirinae
KM236246.1 Bacillus phage Moonbeam- complete genome Moonbeamvirus Bastillevirinae
JX238501.3 Bacillus phage phiAGATE- complete genome Agatevirus Bastillevirinae
KM051843.1 Bacillus phage Bobb- complete genome Agatevirus Bastillevirinae
KJ010547.1 Bacillus phage Bp8p C- complete genome Agatevirus Bastillevirinae
KC699836.1 Bacillus phage SIOphi- partial genome Siophivirus Bastillevirinae
MH606185.1 Bacillus phage BSP38- complete genome Jeonjuvirus Bastillevirinae
KY368640.1 Bacillus phage vB BsuM Goe3- complete genome Grisebachstrassevirus Bastillevirinae
KF669652.1 Bacillus phage Grass- complete genome Nitunavirus Bastillevirinae
AP013029.1 Bacillus phage phiNIT1 DNA- complete genome Nitunavirus Bastillevirinae
AB930182.1 Bacillus phage SPG24 DNA- contig00001 sequence Nitunavirus Bastillevirinae
HQ634192.1 Cellulophaga phage phiST genomic sequence Cbastvirus
KC821632.1 Cellulophaga phage phi4 1- complete genome Lightbulbvirus
KC821609.1 Cellulophaga phage phi17 2- complete genome Lightbulbvirus
MF668286.1 Rhodococcus phage Trina- complete genome Trinavirus
KX774321.1 Rhodococcus phage Weasels2- complete genome Weaselvirus
MG962366.1 Rhodococcus phage Finch- complete genome Finchvirus
MK937592.1 Mycobacterium phage Phrappuccino- complete genome Phrappuccinovirus
MN908687.1 Gordonia phage Skog- complete genome Skogvirus
MK977695.1 Gordonia phage Pupper- complete genome Puppervirus
MF668280.1 Mycobacterium phage Phabba- complete genome Myrnavirus Ceeclamvirinae
EU826466.1 Mycobacterium phage Myrna- complete genome Myrnavirus Ceeclamvirinae
JF704092.1 Mycobacterium phage Alice- complete sequence Bixzunavirus Ceeclamvirinae
JN412588.1 Mycobacterium phage Dandelion- complete genome Bixzunavirus Ceeclamvirinae
AY129337.1 Mycobacteriophage Bxz1- complete genome Bixzunavirus Ceeclamvirinae
AF020713.1 Bacillus phage SPBc2- complete genome Spbetavirus

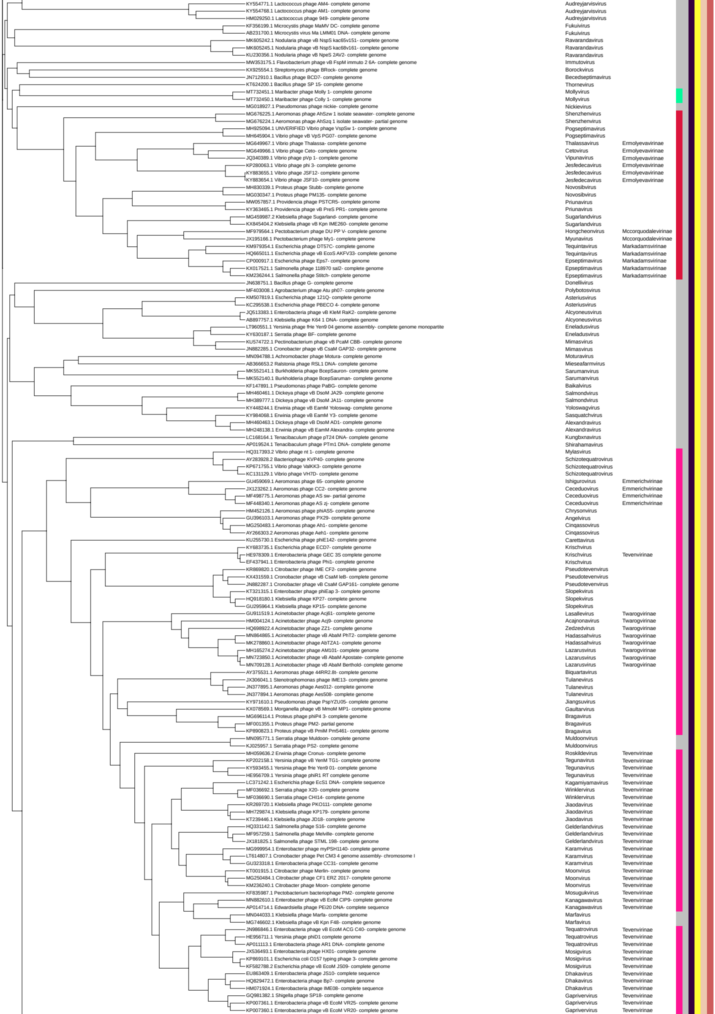

KY554771.1 Lactococcus phage AM4- complete genome Audreyjarvisvirus
KY554768.1 Lactococcus phage AM1- complete genome Audreyjarvisvirus
HM029250.1 Lactococcus phage 949- complete genome Audreyjarvisvirus
KF356199.1 Microcystis phage MaMV DC- complete genome Fukuivirus
AB231700.1 Microcystis virus Ma LMM01 DNA- complete genome Fukuivirus
MK605242.1 Nodularia phage vB NspS kac65v151- complete genome Ravarandavirus
MK605245.1 Nodularia phage vB NspS kac68v161- complete genome Ravarandavirus
KU230356.1 Nodularia phage vB NpeS 2AV2- complete genome Ravarandavirus
MW353175.1 Flavobacterium phage vB FspM immuto 2 6A- complete genome Immutovirus
KX925554.1 Streptomyces phage BRock- complete genome Borockvirus
JN712910.1 Bacillus phage BCD7- complete genome Becedseptimavirus
KT624200.1 Bacillus phage SP 15- complete genome Thornevirus
MT732451.1 Maribacter phage Molly 1- complete genome Mollyvirus
MT732450.1 Maribacter phage Colly 1- complete genome Mollyvirus
MG018927.1 Pseudomonas phage nickie- complete genome Nickievirus
MG676225.1 Aeromonas phage AhSzw 1 isolate seawater- complete genome Shenzhenvirus
MG676224.1 Aeromonas phage AhSzq 1 isolate seawater- partial genome Shenzhenvirus
MH925094.1 UNVERIFIED Vibrio phage VspSw 1- complete genome Pogseptimavirus
MH645904.1 Vibrio phage vB VpS PG07- complete genome Pogseptimavirus
MG649967.1 Vibrio phage Thalassa- complete genome Thalassavirus Ermolyevavirinae
MG649966.1 Vibrio phage Ceto- complete genome Cetovirus Ermolyevavirinae
JQ340389.1 Vibrio phage pVp 1- complete genome Vipunavirus Ermolyevavirinae
KP280063.1 Vibrio phage phi 3- complete genome Jesfedecavirus Ermolyevavirinae
KY883655.1 Vibrio phage JSF12- complete genome Jesfedecavirus Ermolyevavirinae
KY883654.1 Vibrio phage JSF10- complete genome Jesfedecavirus Ermolyevavirinae
MH830339.1 Proteus phage Stubb- complete genome Novosibvirus
MG030347.1 Proteus phage PM135- complete genome Novosibvirus
MW057857.1 Providencia phage PSTCR5- complete genome Priunavirus
KY363465.1 Providencia phage vB PreS PR1- complete genome Priunavirus
MG459987.2 Klebsiella phage Sugarland- complete genome Sugarlandvirus
KX845404.2 Klebsiella phage vB Kpn IME260- complete genome Sugarlandvirus
MF979564.1 Pectobacterium phage DU PP V- complete genome Hongcheonvirus Mccorquodalevirinae
JX195166.1 Pectobacterium phage My1- complete genome Myunavirus Mccorquodalevirinae
KM979354.1 Escherichia phage DT57C- complete genome Tequintavirus Markadamsvirinae
HQ665011.1 Escherichia phage vB EcoS AKFV33- complete genome Tequintavirus Markadamsvirinae
CP000917.1 Escherichia phage Eps7- complete genome Epseptimavirus Markadamsvirinae
KX017521.1 Salmonella phage 118970 sal2- complete genome Epseptimavirus Markadamsvirinae
KM236244.1 Salmonella phage Stitch- complete genome Epseptimavirus Markadamsvirinae
JN638751.1 Bacillus phage G- complete genome Donellivirus
MF403008.1 Agrobacterium phage Atu ph07- complete genome Polybotosvirus
KM507819.1 Escherichia phage 121Q- complete genome Asteriusvirus
KC295538.1 Escherichia phage PBECO 4- complete genome Asteriusvirus
JQ513383.1 Enterobacteria phage vB KleM RaK2- complete genome Alcyoneusvirus
AB897757.1 Klebsiella phage K64 1 DNA- complete genome Alcyoneusvirus
LT960551.1 Yersinia phage fHe Yen9 04 genome assembly- complete genome monopartite Eneladusvirus
KY630187.1 Serratia phage BF- complete genome Eneladusvirus
KU574722.1 Pectinobacterium phage vB PcaM CBB- complete genome Mimasvirus
JN882285.1 Cronobacter phage vB CsaM GAP32- complete genome Mimasvirus
MN094788.1 Achromobacter phage Motura- complete genome Moturavirus
AB366653.2 Ralstonia phage RSL1 DNA- complete genome Mieseafarmvirus
MK552141.1 Burkholderia phage BcepSauron- complete genome Sarumanvirus
MK552140.1 Burkholderia phage BcepSaruman- complete genome Sarumanvirus
KF147891.1 Pseudomonas phage PaBG- complete genome Baikalvirus
MH460461.1 Dickeya phage vB DsoM JA29- complete genome Salmondvirus
MH389777.1 Dickeya phage vB DsoM JA11- complete genome Salmondvirus
KY448244.1 Erwinia phage vB EamM Yoloswag- complete genome Yoloswagvirus
KY984068.1 Erwinia phage vB EamM Y3- complete genome Sasquatchvirus
MH460463.1 Dickeya phage vB DsoM AD1- complete genome Alexandravirus
MH248138.1 Erwinia phage vB EamM Alexandra- complete genome Alexandravirus
LC168164.1 Tenacibaculum phage pT24 DNA- complete genome Kungbxnavirus
AP019524.1 Tenacibaculum phage PTm1 DNA- complete genome Shirahamavirus
HQ317393.2 Vibrio phage nt 1- complete genome Mylasvirus
AY283928.2 Bacteriophage KVP40- complete genome Schizotequatrovirus
KP671755.1 Vibrio phage ValKK3- complete genome Schizotequatrovirus
KC131129.1 Vibrio phage VH7D- complete genome Schizotequatrovirus
GU459069.1 Aeromonas phage 65- complete genome Ishigurovirus Emmerichvirinae
JX123262.1 Aeromonas phage CC2- complete genome Ceceduovirus Emmerichvirinae
MF498775.1 Aeromonas phage AS sw- partial genome Ceceduovirus Emmerichvirinae
MF448340.1 Aeromonas phage AS zj- complete genome Ceceduovirus Emmerichvirinae
HM452126.1 Aeromonas phage phiAS5- complete genome Chrysonvirus
GU396103.1 Aeromonas phage PX29- complete genome Angelvirus
MG250483.1 Aeromonas phage Ah1- complete genome Cinqassovirus
AY266303.2 Aeromonas phage Aeh1- complete genome Cinqassovirus
KU255730.1 Escherichia phage phiE142- complete genome Carettavirus
KY683735.1 Escherichia phage ECD7- complete genome Krischvirus
HE978309.1 Enterobacteria phage GEC 3S complete genome Krischvirus Tevenvirinae
EF437941.1 Enterobacteria phage Phi1- complete genome Krischvirus
KR869820.1 Citrobacter phage IME CF2- complete genome Pseudotevenvirus
KX431559.1 Cronobacter phage vB CsaM leB- complete genome Pseudotevenvirus
JN882287.1 Cronobacter phage vB CsaM GAP161- complete genome Pseudotevenvirus
KT321315.1 Enterobacter phage phiEap 3- complete genome Slopekvirus
HQ918180.1 Klebsiella phage KP27- complete genome Slopekvirus
GU295964.1 Klebsiella phage KP15- complete genome Slopekvirus
GU911519.1 Acinetobacter phage Acj61- complete genome Lasallevirus Twarogvirinae
HM004124.1 Acinetobacter phage Acj9- complete genome Acajnonavirus Twarogvirinae
HQ698922.4 Acinetobacter phage ZZ1- complete genome Zedzedvirus Twarogvirinae
MN864865.1 Acinetobacter phage vB AbaM PhT2- complete genome Hadassahvirus Twarogvirinae
MK278860.1 Acinetobacter phage AbTZA1- complete genome Hadassahvirus Twarogvirinae
MH165274.2 Acinetobacter phage AM101- complete genome Lazarusvirus Twarogvirinae
MN723850.1 Acinetobacter phage vB AbaM Apostate- complete genome Lazarusvirus Twarogvirinae
MN709128.1 Acinetobacter phage vB AbaM Berthold- complete genome Lazarusvirus Twarogvirinae
AY375531.1 Aeromonas phage 44RR2.8t- complete genome Biquartavirus
JX306041.1 Stenotrophomonas phage IME13- complete genome Tulanevirus
JN377895.1 Aeromonas phage Aes012- complete genome Tulanevirus
JN377894.1 Aeromonas phage Aes508- complete genome Tulanevirus
KY971610.1 Pseudomonas phage PspYZU05- complete genome Jiangsuvirus
KX078569.1 Morganella phage vB MmoM MP1- complete genome Gaultarvirus
MG696114.1 Proteus phage phiP4 3- complete genome Bragavirus
MF001355.1 Proteus phage PM2- partial genome Bragavirus
KP890823.1 Proteus phage vB PmiM Pm5461- complete genome Bragavirus
MN095771.1 Serratia phage Muldoon- complete genome Muldoonvirus
KJ025957.1 Serratia phage PS2- complete genome Muldoonvirus
MH059636.2 Erwinia phage Cronus- complete genome Roskildevirus Tevenvirinae
KP202158.1 Yersinia phage vB YenM TG1- complete genome Tegunavirus Tevenvirinae
KY593455.1 Yersinia phage fHe Yen9 01- complete genome Tegunavirus Tevenvirinae
HE956709.1 Yersinia phage phiR1 RT complete genome Tegunavirus Tevenvirinae
LC371242.1 Escherichia phage EcS1 DNA- complete sequence Kagamiyamavirus Tevenvirinae
MF036692.1 Serratia phage X20- complete genome Winklervirus Tevenvirinae
MF036690.1 Serratia phage CHI14- complete genome Winklervirus Tevenvirinae
KR269720.1 Klebsiella phage PKO111- complete genome Jiaodavirus Tevenvirinae
MH729874.1 Klebsiella phage KP179- complete genome Jiaodavirus Tevenvirinae
KT239446.1 Klebsiella phage JD18- complete genome Jiaodavirus Tevenvirinae
HQ331142.1 Salmonella phage S16- complete genome Gelderlandvirus Tevenvirinae
MF957259.1 Salmonella phage Melville- complete genome Gelderlandvirus Tevenvirinae
JX181825.1 Salmonella phage STML 198- complete genome Gelderlandvirus Tevenvirinae
MG999954.1 Enterobacter phage myPSH1140- complete genome Karamvirus Tevenvirinae
LT614807.1 Cronobacter phage Pet CM3 4 genome assembly- chromosome I Karamvirus Tevenvirinae
GU323318.1 Enterobacteria phage CC31- complete genome Karamvirus Tevenvirinae
KT001915.1 Citrobacter phage Merlin- complete genome Moonvirus Tevenvirinae
MG250484.1 Citrobacter phage CF1 ERZ 2017- complete genome Moonvirus Tevenvirinae
KM236240.1 Citrobacter phage Moon- complete genome Moonvirus Tevenvirinae
KF835987.1 Pectobacterium bacteriophage PM2- complete genome Mosugukvirus Tevenvirinae
MN882610.1 Enterobacter phage vB EclM CIP9- complete genome Kanagawavirus Tevenvirinae
AP014714.1 Edwardsiella phage PEi20 DNA- complete sequence Kanagawavirus Tevenvirinae
MN044033.1 Klebsiella phage Marfa- complete genome Marfavirus
MG746602.1 Klebsiella phage vB Kpn F48- complete genome Marfavirus
JN986846.1 Enterobacteria phage vB EcoM ACG C40- complete genome Tequatrovirus Tevenvirinae
HE956711.1 Yersinia phage phiD1 complete genome Tequatrovirus Tevenvirinae
AP011113.1 Enterobacteria phage AR1 DNA- complete genome Tequatrovirus Tevenvirinae
JX536493.1 Enterobacteria phage HX01- complete genome Mosigvirus Tevenvirinae
KP869101.1 Escherichia coli O157 typing phage 3- complete genome Mosigvirus Tevenvirinae
KF582788.2 Escherichia phage vB EcoM JS09- complete genome Mosigvirus Tevenvirinae
EU863409.1 Enterobacteria phage JS10- complete sequence Dhakavirus Tevenvirinae
HQ829472.1 Enterobacteria phage Bp7- complete genome Dhakavirus Tevenvirinae
HM071924.1 Enterobacteria phage IME08- complete sequence Dhakavirus Tevenvirinae
GQ981382.1 Shigella phage SP18- complete genome Gaprivervirus Tevenvirinae
KP007361.1 Enterobacteria phage vB EcoM VR25- complete genome Gaprivervirus Tevenvirinae
KP007360.1 Enterobacteria phage vB EcoM VR20- complete genome Gaprivervirus Tevenvirinae

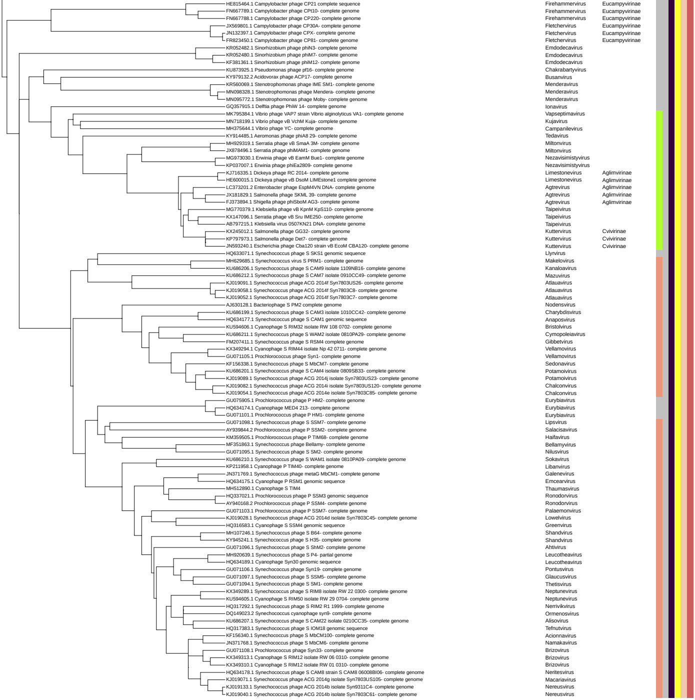

HE815464.1 Campylobacter phage CP21 complete sequence Firehammervirus Eucampyvirinae
FN667789.1 Campylobacter phage CPt10- complete genome Firehammervirus Eucampyvirinae
FN667788.1 Campylobacter phage CP220- complete genome Firehammervirus Eucampyvirinae
JX569801.1 Campylobacter phage CP30A- complete genome Fletchervirus Eucampyvirinae
JN132397.1 Campylobacter phage CPX- complete genome Fletchervirus Eucampyvirinae
FR823450.1 Campylobacter phage CP81- complete genome Fletchervirus Eucampyvirinae
KR052482.1 Sinorhizobium phage phiN3- complete genome Emdodecavirus
KR052480.1 Sinorhizobium phage phiM7- complete genome Emdodecavirus
KF381361.1 Sinorhizobium phage phiM12- complete genome Emdodecavirus
KU873925.1 Pseudomonas phage pf16- complete genome Chakrabartyvirus
KY979132.2 Acidovorax phage ACP17- complete genome Busanvirus
KR560069.1 Stenotrophomonas phage IME SM1- complete genome Menderavirus
MN098328.1 Stenotrophomonas phage Mendera- complete genome Menderavirus
MN095772.1 Stenotrophomonas phage Moby- complete genome Menderavirus
GQ357915.1 Delftia phage PhiW 14- complete genome Ionavirus
MK795384.1 Vibrio phage VAP7 strain Vibrio alginolyticus VA1- complete genome Vapseptimavirus
MN718199.1 Vibrio phage vB VchM Kuja- complete genome Kujavirus
MH375644.1 Vibrio phage YC- complete genome Campanilevirus
KY914485.1 Aeromonas phage phiA8 29- complete genome Tedavirus
MH929319.1 Serratia phage vB SmaA 3M- complete genome Miltonvirus
JX878496.1 Serratia phage phiMAM1- complete genome Miltonvirus
MG973030.1 Erwinia phage vB EamM Bue1- complete genome Nezavisimistyvirus
KP037007.1 Erwinia phage phiEa2809- complete genome Nezavisimistyvirus
KJ716335.1 Dickeya phage RC 2014- complete genome Limestonevirus Aglimvirinae
HE600015.1 Dickeya phage vB DsoM LIMEstone1 complete genome Limestonevirus Aglimvirinae
LC373201.2 Enterobacter phage EspM4VN DNA- complete genome Agtrevirus Aglimvirinae
JX181829.1 Salmonella phage SKML 39- complete genome Agtrevirus Aglimvirinae
FJ373894.1 Shigella phage phiSboM AG3- complete genome Agtrevirus Aglimvirinae
MG770379.1 Klebsiella phage vB KpnM KpS110- complete genome Taipeivirus
KX147096.1 Serratia phage vB Sru IME250- complete genome Taipeivirus
AB797215.1 Klebsiella virus 0507KN21 DNA- complete genome Taipeivirus
KX245012.1 Salmonella phage GG32- complete genome Kuttervirus Cvivirinae
KP797973.1 Salmonella phage Det7- complete genome Kuttervirus Cvivirinae
JN593240.1 Escherichia phage Cba120 strain vB EcoM CBA120- complete genome Kuttervirus Cvivirinae
HQ633071.1 Synechococcus phage S SKS1 genomic sequence Llyrvirus
MH629685.1 Synechococcus virus S PRM1- complete genome Makelovirus
KU686206.1 Synechococcus phage S CAM9 isolate 1109NB16- complete genome Kanaloavirus
KU686212.1 Synechococcus phage S CAM7 isolate 0910CC49- complete genome Mazuvirus
KJ019091.1 Synechococcus phage ACG 2014f Syn7803US26- complete genome Atlauavirus
KJ019058.1 Synechococcus phage ACG 2014f Syn7803C8- complete genome Atlauavirus
KJ019052.1 Synechococcus phage ACG 2014f Syn7803C7- complete genome Atlauavirus
AJ630128.1 Bacteriophage S PM2 complete genome Nodensvirus
KU686199.1 Synechococcus phage S CAM3 isolate 1010CC42- complete genome Charybdisvirus
HQ634177.1 Synechococcus phage S CAM1 genomic sequence Anaposvirus
KU594606.1 Cyanophage S RIM32 isolate RW 108 0702- complete genome Bristolvirus
KU686211.1 Synechococcus phage S WAM2 isolate 0810PA29- complete genome Cymopoleiavirus
FM207411.1 Synechococcus phage S RSM4 complete genome Gibbetvirus
KX349294.1 Cyanophage S RIM44 isolate Np 42 0711- complete genome Vellamovirus
GU071105.1 Prochlorococcus phage Syn1- complete genome Vellamovirus
KF156338.1 Synechococcus phage S MbCM7- complete genome Sedonavirus
KU686201.1 Synechococcus phage S CAM4 isolate 0809SB33- complete genome Potamoivirus
KJ019089.1 Synechococcus phage ACG 2014j isolate Syn7803US23- complete genome Potamoivirus
KJ019082.1 Synechococcus phage ACG 2014i isolate Syn7803US120- complete genome Chalconvirus
KJ019054.1 Synechococcus phage ACG 2014e isolate Syn7803C85- complete genome Chalconvirus
GU075905.1 Prochlorococcus phage P HM2- complete genome Eurybiavirus
HQ634174.1 Cyanophage MED4 213- complete genome Eurybiavirus
GU071101.1 Prochlorococcus phage P HM1- complete genome Eurybiavirus
GU071098.1 Synechococcus phage S SSM7- complete genome Lipsvirus
AY939844.2 Prochlorococcus phage P SSM2- complete genome Salacisavirus
KM359505.1 Prochlorococcus phage P TIM68- complete genome Haifavirus
MF351863.1 Synechococcus phage Bellamy- complete genome Bellamyvirus
GU071095.1 Synechococcus phage S SM2- complete genome Nilusvirus
KU686210.1 Synechococcus phage S WAM1 isolate 0810PA09- complete genome Sokavirus
KP211958.1 Cyanophage P TIM40- complete genome Libanvirus
JN371769.1 Synechococcus phage metaG MbCM1- complete genome Galenevirus
HQ634175.1 Cyanophage P RSM1 genomic sequence Emcearvirus
MH512890.1 Cyanophage S TIM4 Thaumasvirus
HQ337021.1 Prochlorococcus phage P SSM3 genomic sequence Ronodorvirus
AY940168.2 Prochlorococcus phage P SSM4- complete genome Ronodorvirus
GU071103.1 Prochlorococcus phage P SSM7- complete genome Palaemonvirus
KJ019028.1 Synechococcus phage ACG 2014d isolate Syn7803C45- complete genome Lowelvirus
HQ316583.1 Cyanophage S SSM4 genomic sequence Greenvirus
MH107246.1 Synechococcus phage S B64- complete genome Shandvirus
KY945241.1 Synechococcus phage S H35- complete genome Shandvirus
GU071096.1 Synechococcus phage S ShM2- complete genome Ahtivirus
MH920639.1 Synechococcus phage S P4- partial genome Leucotheavirus
HQ634189.1 Cyanophage Syn30 genomic sequence Leucotheavirus
GU071106.1 Synechococcus phage Syn19- complete genome Pontusvirus
GU071097.1 Synechococcus phage S SSM5- complete genome Glaucusvirus
GU071094.1 Synechococcus phage S SM1- complete genome Thetisvirus
KX349289.1 Synechococcus phage S RIM8 isolate RW 22 0300- complete genome Neptunevirus
KU594605.1 Cyanophage S RIM50 isolate RW 29 0704- complete genome Neptunevirus
HQ317292.1 Synechococcus phage S RIM2 R1 1999- complete genome Nerrivikvirus
DQ149023.2 Synechococcus cyanophage syn9- complete genome Ormenosvirus
KU686207.1 Synechococcus phage S CAM22 isolate 0210CC35- complete genome Alisovirus
HQ317383.1 Synechococcus phage S IOM18 genomic sequence Tefnutvirus
KF156340.1 Synechococcus phage S MbCM100- complete genome Acionnavirus
JN371768.1 Synechococcus phage S MbCM6- complete genome Namakavirus
GU071108.1 Prochlorococcus phage Syn33- complete genome Brizovirus
KX349313.1 Cyanophage S RIM12 isolate RW 06 0310- complete genome Brizovirus
KX349310.1 Cyanophage S RIM12 isolate RW 01 0310- complete genome Brizovirus
HQ634178.1 Synechococcus phage S CAM8 strain S CAM8 06008BI06- complete genome Neritesvirus
KJ019071.1 Synechococcus phage ACG 2014g isolate Syn7803US105- complete genome Macariavirus
KJ019133.1 Synechococcus phage ACG 2014b isolate Syn9311C4- complete genome Nereusvirus
KJ019040.1 Synechococcus phage ACG 2014b isolate Syn7803C61- complete genome Nereusvirus
