# Supplementary material for: VirClust—A Tool for Hierarchical Clustering, Core Protein Detection and Annotation of (Prokaryotic) Viruses
Source: Viruses. 2023 Apr 19;15(4):1007. doi: 10.3390/v15041007 (PMC10143988; doi:10.3390/v15041007)
Supplement: Supplementary file 1 [file SI_File8_Fam_DB_PSC_trees_extended.pdf › SI_File8_Fam_DB_PSC_trees_extended.pdf]

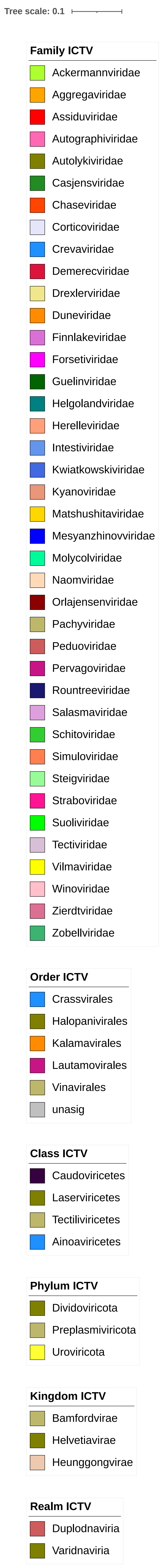

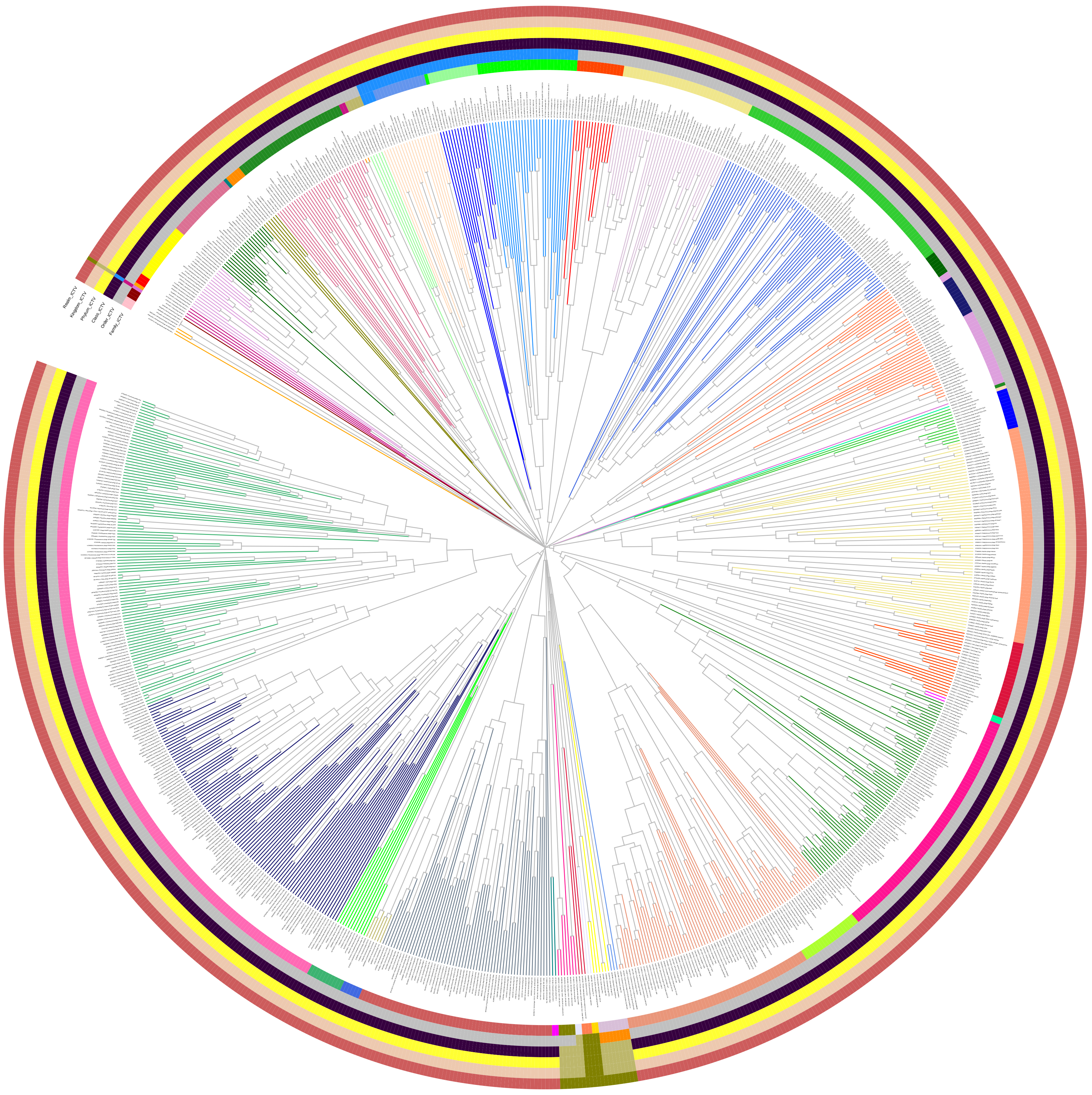

Fig. S1: Circular representation of the PSC-based genome tree for the Fam_DB dataset. The annotation circles encode the ICTV taxonomy for each phage genome, as follows from inner to outer circles: family, order, class, phylum, kingdom, and realm. The tree was split using a distance threshold of 0.9 and the resulting VGCs are encoded by different branch colors

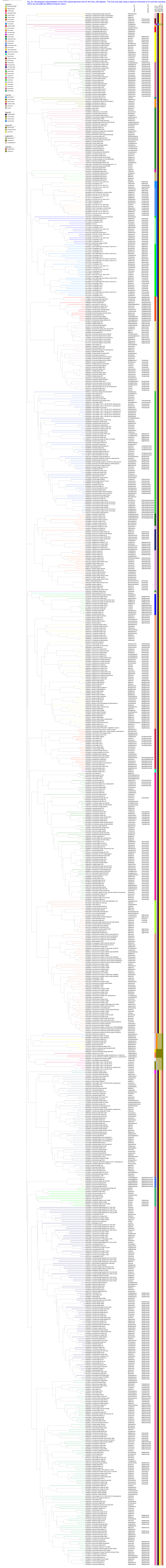

Fig. S1: Rectangular representation of the PSC-based genome tree for the Fam_DB dataset. The tree was split using a distance threshold of 0.9 and the resulting VGCs are encoded by different branch colors
