# Supplementary material for: VirClust—A Tool for Hierarchical Clustering, Core Protein Detection and Annotation of (Prokaryotic) Viruses
Source: Viruses. 2023 Apr 19;15(4):1007. doi: 10.3390/v15041007 (PMC10143988; doi:10.3390/v15041007)
Supplement: Supplementary file 1 [file viruses-15-01007-s001.zip › SI_File2_VirClust-v2_manual_web-server.pdf]

VirClust v2 web-server – user manual

Developer: Cristina Moraru, email liliana.cristina.moraru@uni-oldenburg.de

Web-server: virclust.icbm.de

The interface for the VirClust WEB is organized in two Tab Panels, one for defining the project and the other for running the calculations (see Figure 1).

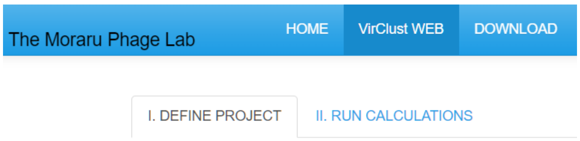

Figure 1: The main Tab Panels in VirClust WEB.

Define project panel

VirClust assigns to each input dataset a project and a project ID. Once a project has been created, it can be re-loaded at a later time, and the calculations continued or repeated with other parameters.

Creating a new project

To create a new project (Figure 2), the user needs to provide:

- A user name and a project name. These will be used by VirClust to compose a unique project ID.
- An input genomic dataset, either as i) a single multi-fasta file, containing all the genomes; or ii) as a set of single fasta file, each containing a single contig (considered further as a genome).

Once the above information has been provided, the “Create project” button becomes active and the user can click it to create a new project. The ID of the project will be displayed in the “Info Board” (Figure 3).

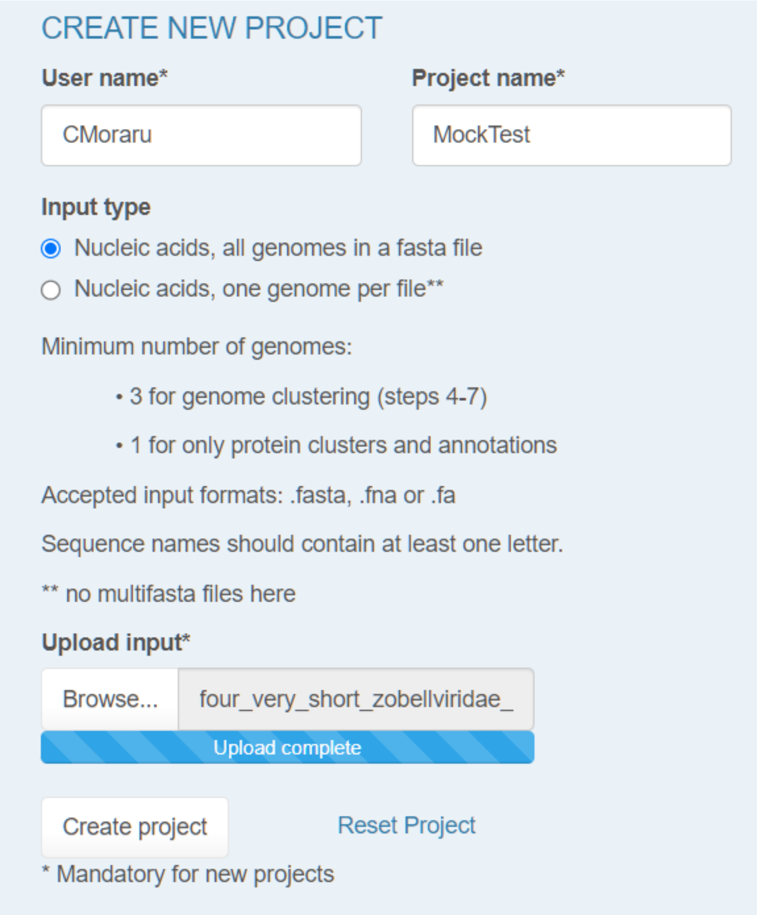

Figure 2: The "Create new project" panel.

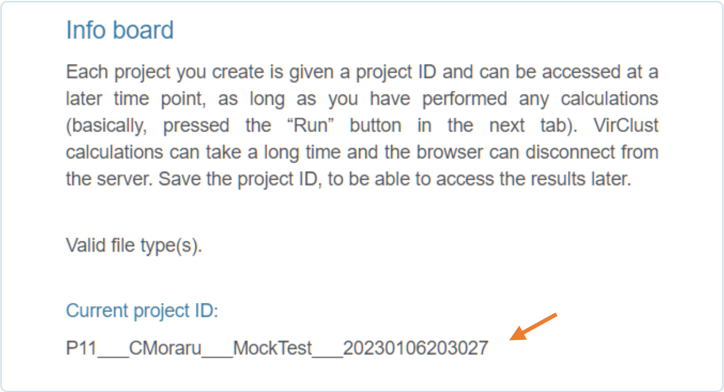

Figure 3: Info Board. Once the new project has been created, its ID is displayed here - see orange arrow.

Loading an existing project

Sometimes, the calculations for a certain step can take long times, especially for large datasets and the user wants to turn off the browser or his computer and access the project the next day. Or, after a time has passed since the last calculations, the user decides to further analyze her/his data, or to re-calculate some steps with other parameters. This is possible by using the project ID to re-load the data, as shown in Figure 4. Once the project has been loaded, a message about its status is shown in the "Info Board" (Figure 5).

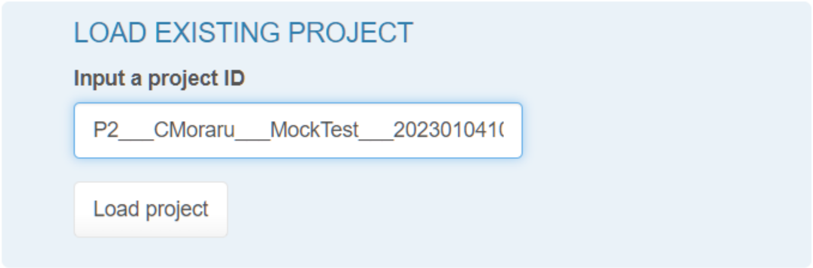

Figure 4: The "Load existing project" Panel. Use the project ID of a previously created project to re-load it in VirClust.

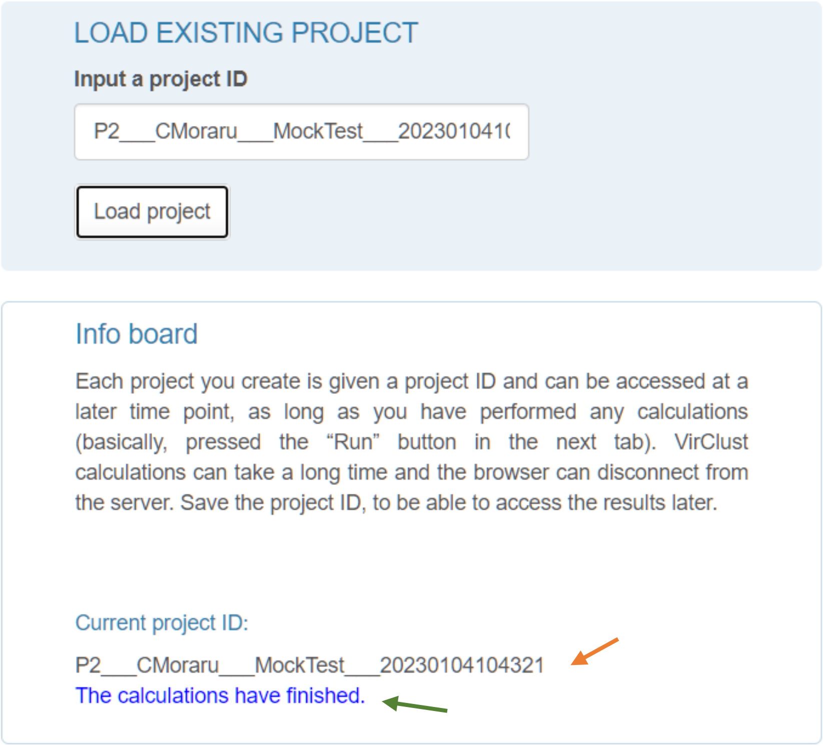

Figure 5: Info Board. Once the existing project has been loaded, its ID (orange arrow), as well as its status (green arrow), are displayed here.

Run calculations Panel

The *Run calculations* Tab Panel is organized into three further Tab Panels, corresponding to each branch of the VirClust workflow: i) Branch A, based on protein clusters, ii) Branch B, based on protein superclusters, and, iii) Branch C, based on protein super-super clusters (see Figure 6).

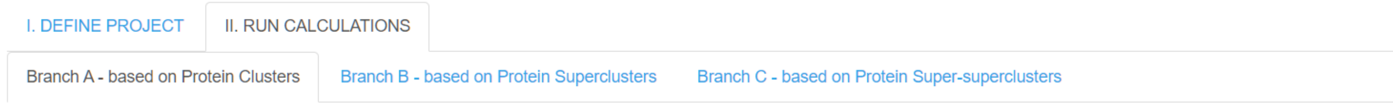

Figure 6: The three branches of the VirClust workflow.

Each Branch is further organized into four modules, each consisting of single or multiple steps (Figure 7). Within a branch, most of the steps can be performed only sequentially, only after the previous step has been performed. Therefore, the corresponding start calculations buttons and parameter selectors for each step only become active if the prerequisite step has been run (see dependencies in Figure 7). Similarly, the download results buttons for each step only become active after the corresponding calculations have been performed.

Individual steps can also be re-calculated, for example with a different set of parameters. In this case, all the results from downstream steps (steps depending on the step to be re-calculated, see dependencies in Figure 7) will be removed from the project.

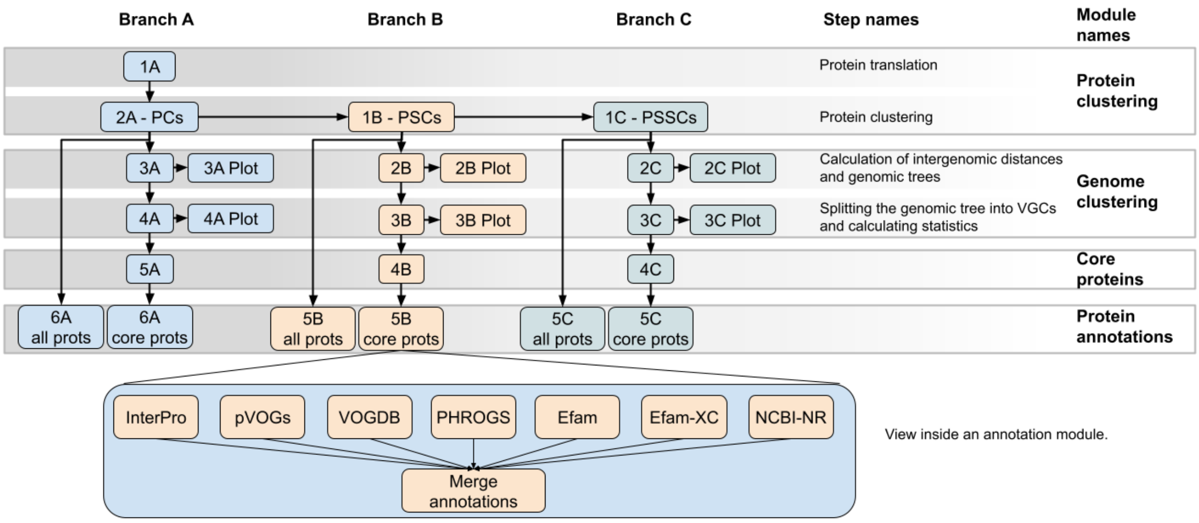

Figure 7: VirClust workflow: dependencies between branches and steps. A step that depends on another step (indicated by the arrow direction) will become active only after the first step has been performed. Re-calculation of one step will remove the results from all dependent steps For example, step 3A_Plot will become active only after steps 1A, 2A and 3A have been performed. Re-calculation of the 3A step will remove 3A_Plot, 4A, 4A_Plot, 5A and 6A_core. Inside one annotation module, all steps for searching in databases are activated at the same time. The "merge annotations" step is activated only when at last one database has been queried.

Branch A – Protein clustering module

Step 1A – from genomes to proteins

The first step, performing gene prediction and protein translation (Figure 8), is the prerequisite for all the other steps in the VirClust workflow. The user can chose the genetic code for translation (Figure 9). The default genetic code is the number 11, for bacteria, archaea, prokaryotic viruses and plant plastids. However, the user can chose other genetic codes for translations, in case the viruses infect other hosts or have non-canonical nucleotides. After the calculations have been successfully performed, the download outputs buttons become active (Figure 10). The user can download: i) a .zip archive with a protein file (.faa) per genome, ii) a .faa file with all proteins from all genomes and iii) a "genome and protein table" in .tsv format, with all genomes and all their predicted genes and proteins. Within a project, each protein recieves a unique identifier (proteinID), which is independent of the genome name. This is necessary to prevent possible problems in the upstream steps due to varying genome name formats and lengths. The correspondence between the proteinID and its corresponding gene and genome can be retrieved from the "genome and protein table".

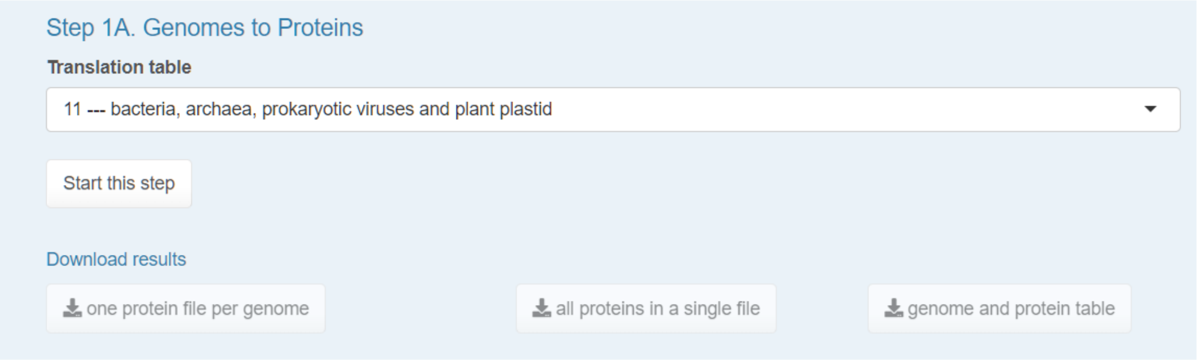

Figure 8: Step 1A – view before calculations, when only the "Translation table" selector menu and the "start this step" button are active.

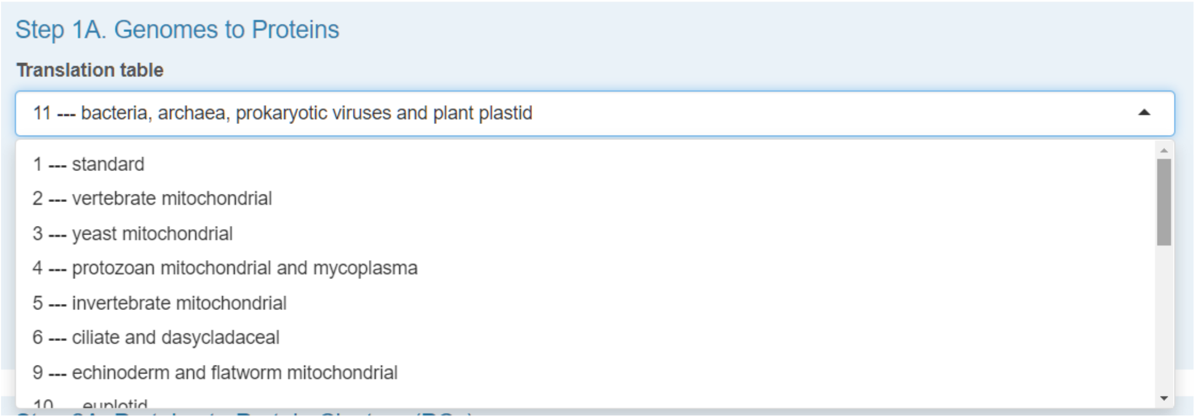

Figure 9: Step 1A – select the desired genetic code for translation by clicking on the corresponding option in the selector menu.

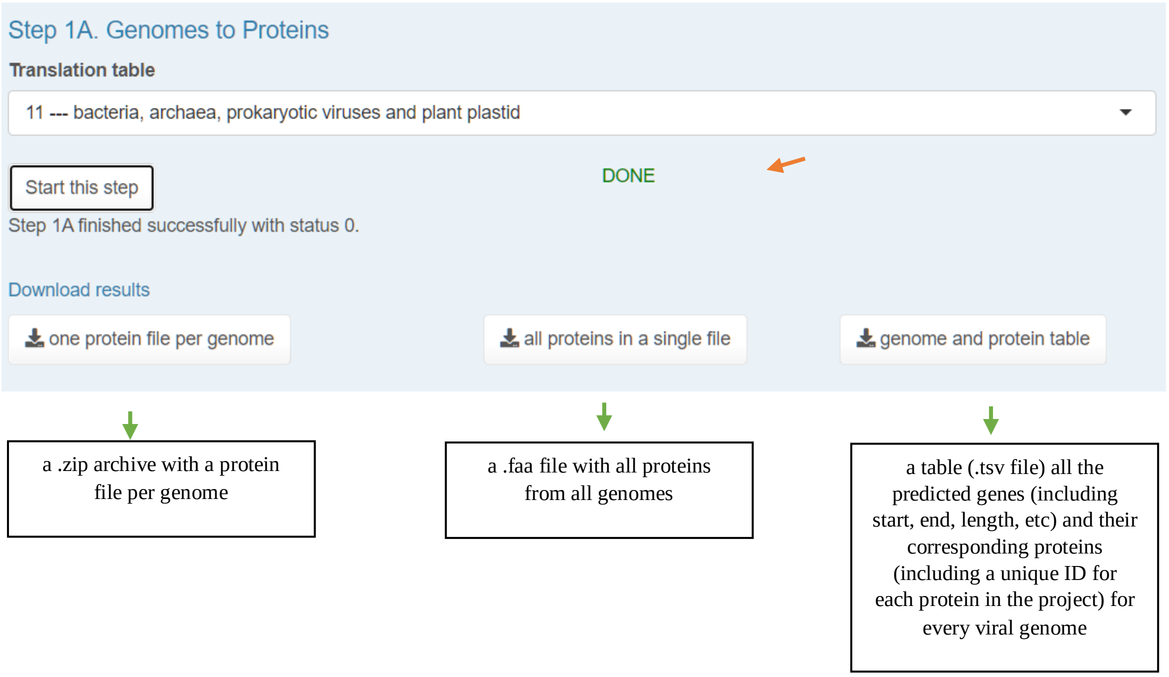

Figure 10: Step 1A – view after calculations, when the "download results" buttons are active as well. The orange arrows indicate the "DONE" message, which is displayed next to each "start this step" button after the calculations have been performed.

Step 2A – from proteins to protein clusters (PCs)

In step2A, the proteins from all viral genomes (produced as step 1A) are grouped into clusters, based on their BLASTP similarity (Figure 11). The grouping into clusters will be affected by: i) the value used as proxy for the similarity of two proteins in a query-subject BLASTP result pair; and ii) the filtering thresholds for the query-subject pairs, which control which of the pairs resulting from a BLASTP are representing truly similar proteins (and will be kept for clustering) and which not (and will be removed before clustering).

There are four values that can be used as proxy for protein similarity for each query-subject pairs (Figure 12): i) the evalue; ii) the log-evalue, representing the log10 transformed evalue, with a cap at 200; iii) the bitscore; and, iv) the normalized bitscore (for a pair of two proteins, the normalized bitscore is calculatd as the maximum from "bitscore for prot1-prot2 hit / bitscore for prot1-prot1 hit" and "bitscore for prot2-prot1 hit / bitscore for prot2-prot2 hit").

The query-subject pairs can be removed before clustering if their: i) e-value is larger than the given value (default is > 0.00001); ii) bitscore is smaller than the given value (default is < 50); iii) coverage is smaller than the given value (default is < 0, meaning that coverage is not taken into account at filtering); and, iv) the % identity is smaller than the given value (default is < 0, meaning that protein identity is not taken into account at filtering).

After calculating this step, the user can download a genome-protein table similar to the one from step 1A, to which a column with the corresponding PCs for each protein has been added.

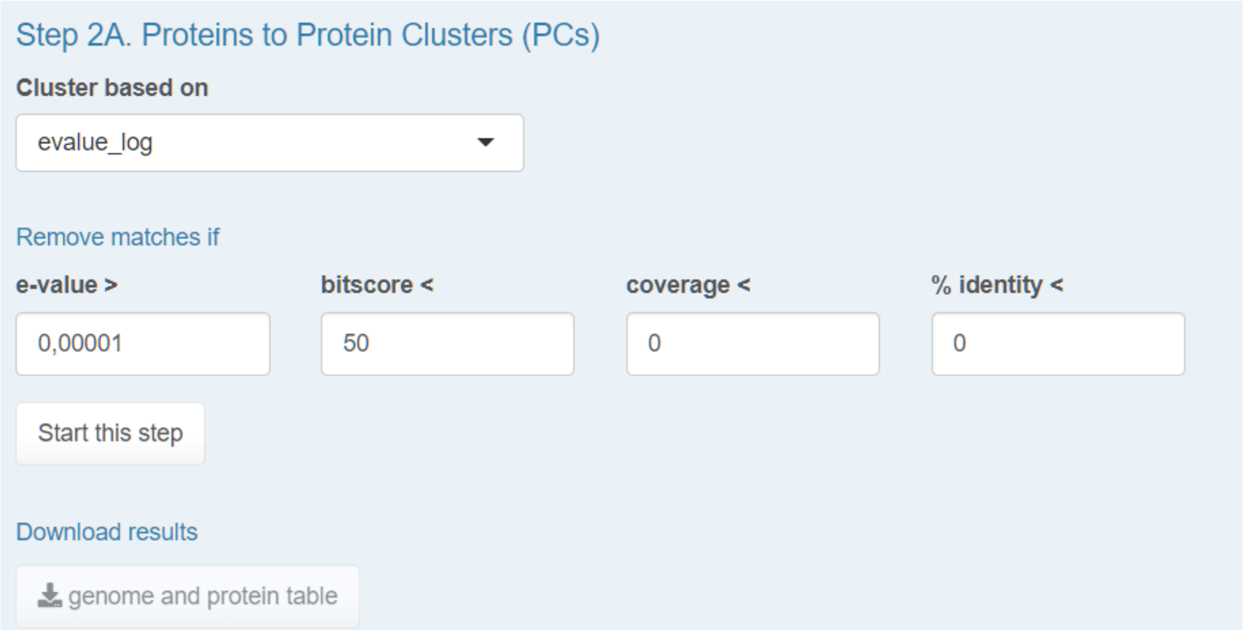

Figure 11: Step 2A - from proteins to PCs. The coverage and the % identity parameters have a maximum of 100 (any value above 100 is automatically transformed to 100).

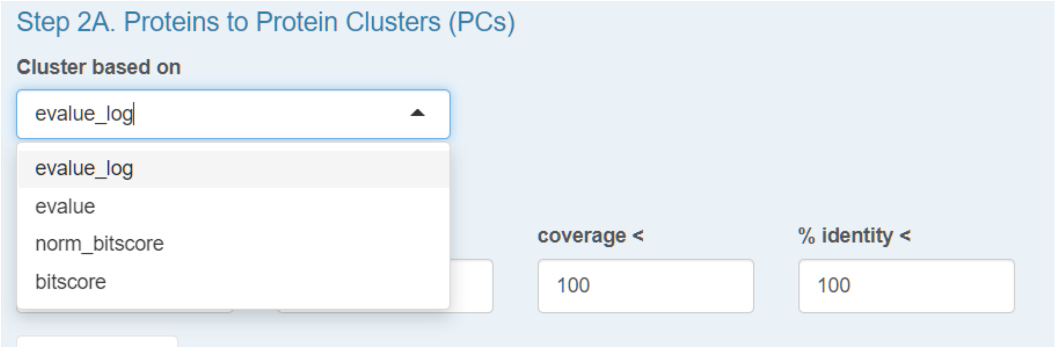

Figure 12: Step 2A - selecting the values used as proxy for protein similarity during protein clustering.

Branch A – genome clustering module

Step 3A – hierarchical clustering of viral genomes

In step 3A (Figure 13), for each viral genome pair, an intergenomic distance is calculated based on their PC content. Further, these distances are used to produce a hierarchical clustering tree of the viral genomes. Two methods are available for tree calculation: “complete” and “average”. As outputs, the user can download: i) a hierarchical clustering tree file, in the .newick format, which can be visualized in software like FigTree (http://tree.bio.ed.ac.uk/software/figtree/) or iTOl (https://itol.embl.de/); and ii) a matrix of the intergenomic distances, in the .tsv format.

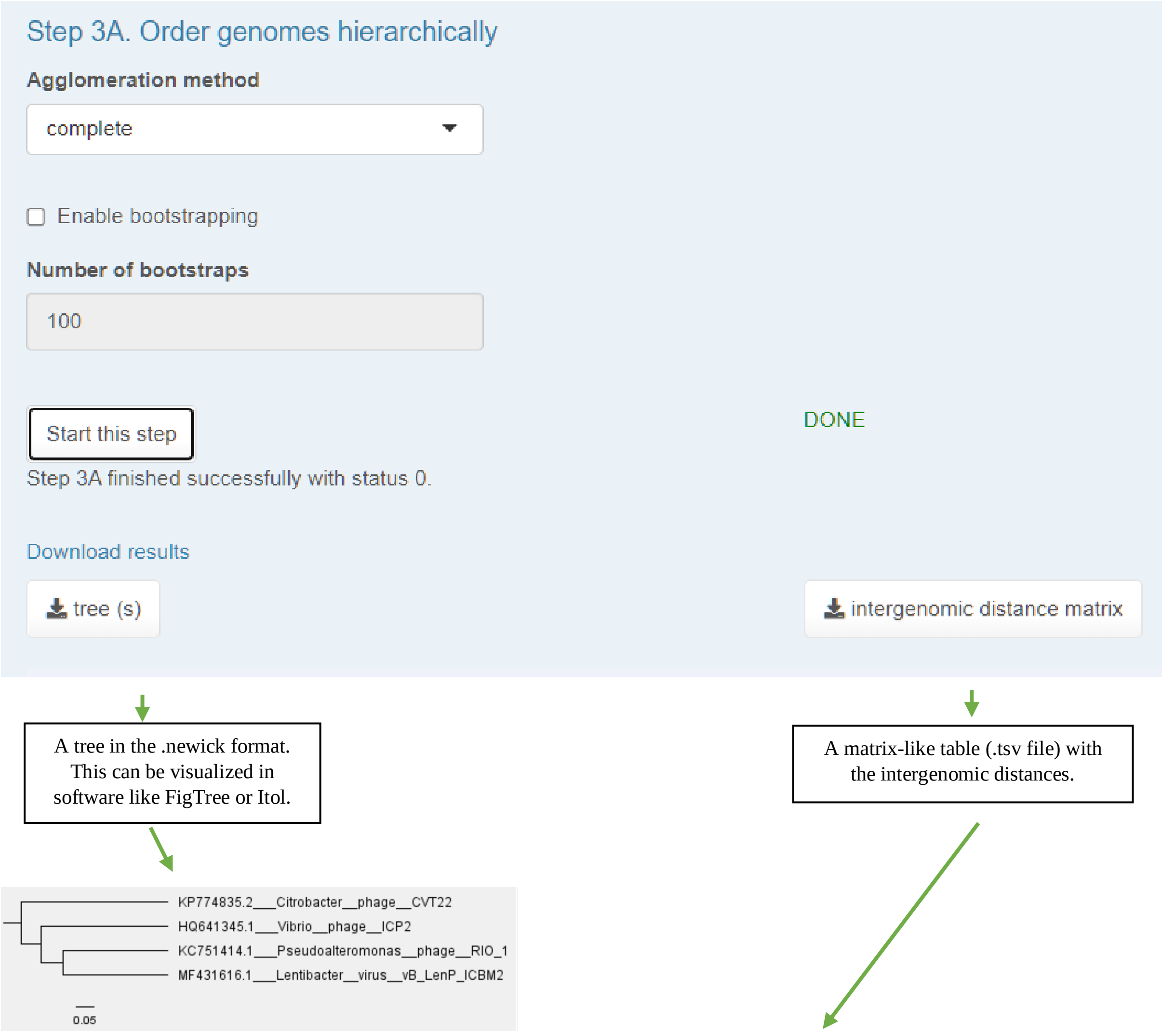

genome_name	KP774835.2___Citrob	HQ641345.1___Vibrio__ph	KC751414.1___Pseuc	MF431616.1___Lentibacter_
KP774835.2___Citrobacter__phage__CVT22	0,00	0,80	0,69	0,64
HQ641345.1___Vibrio__phage__ICP2	0,80	0,00	0,69	0,64
KC751414.1___Pseudoalteromonas__phage__RIO_	0,69	0,69	0,00	0,57
MF431616.1___Lentibacter__virus__vB_LenP_ICBN	0,64	0,64	0,57	0,00

Figure 13: Step 3A – bootstrapping is disabled (default)

If the bootstrapping option is enabled (Figure 14), then the user can choose the number of bootstraps to be performed during intergenomic distance and tree calculations. The following probabilities will be calculated for each internal node in the tree: i) selective inference p-value (SI); ii) approximately unbiased p-value (AU); and iii) bootstrap probability (BP) value. As a result, instead of one tree, the user can download a .zip archive containing three trees, one for each probability (Figure

14). These trees, including their internal node probabilities, can be visualized in software like FigTree (http://tree.bio.ed.ac.uk/software/figtree/) or iTOl (https://itol.embl.de/). The bootstrapping option is not active in the web-server if the number of genomes exceeds 50, due to computational resources limitations.

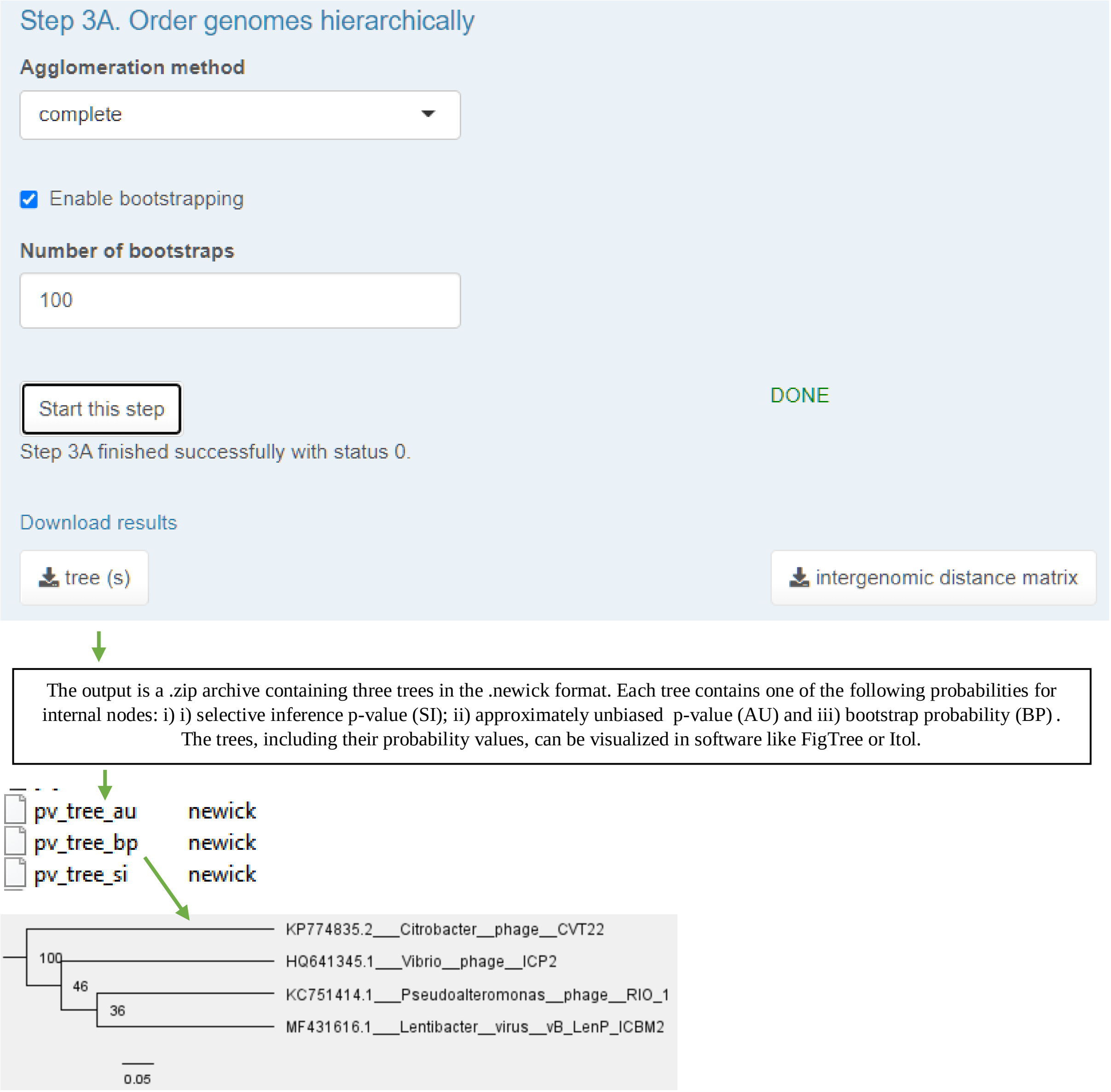

Figure 14: Step 3A - bootstrapping checkbox is enabled.

Data visualization sub-step "Plot intergenomic similarities"

The intergenomic distances can be downloaded as a matrix-like table in .tsv format, which can be opened in software like Excell. In addition, step 3A contains an data-visualization sub-step, named "Plot intergenomic similarities". In this step, the user can plot the intergenomic similarities (calculated as "1 – intergenomic distance") for all input genomes as an ordered, color-coded heatmap (Figure 15). Several options for the formatting of the heatmap are available:

- *cell width* controls the size of the square in which the intergenomic similarity for each genome pair is displayed
- *row font* controls the size of the genome names from the rows of the heatmap
- *column font* controls the size of the genome names from the columns of the heatmap
- *cell font* controls the font size for the intergenomic similarities

- *legend font* and *legend label font* control the font size of the legend title and of the legend labels, respectively
- *legend height* and *legend width* control the height and width of the legend

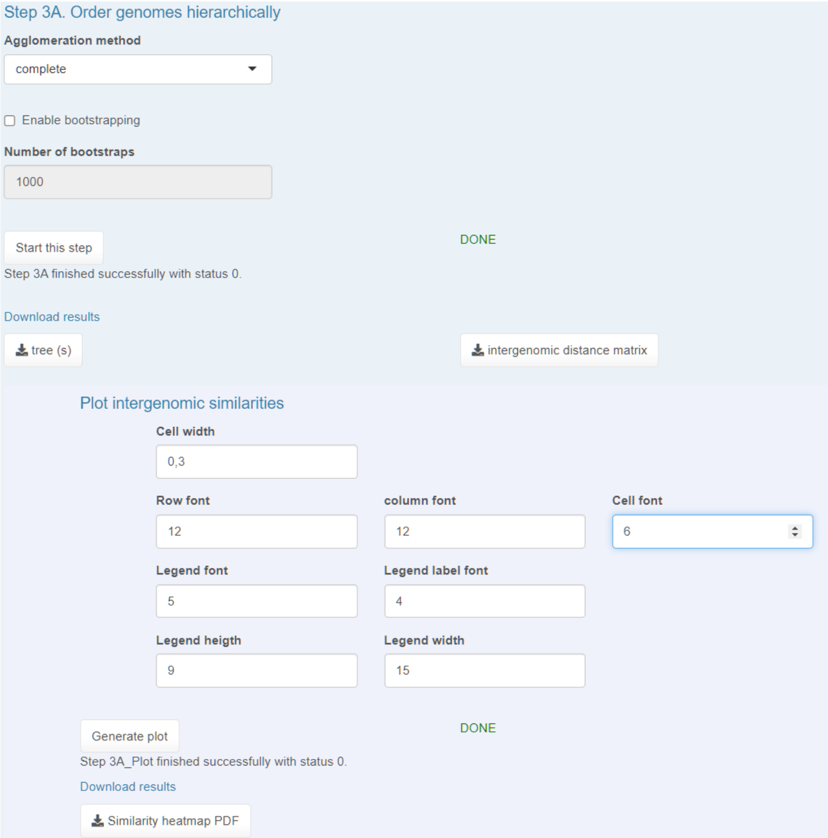

The output is PDF file containign an **<u>ordered, color-coded heatmap</u>** of the intergenomic similarities (the reverse of the intergenomic distances). The intergenomic similarity for each genome pair is displayed as a number in the heatmap. Several options for the formating of the heatmap are available (e.g. "cell width"

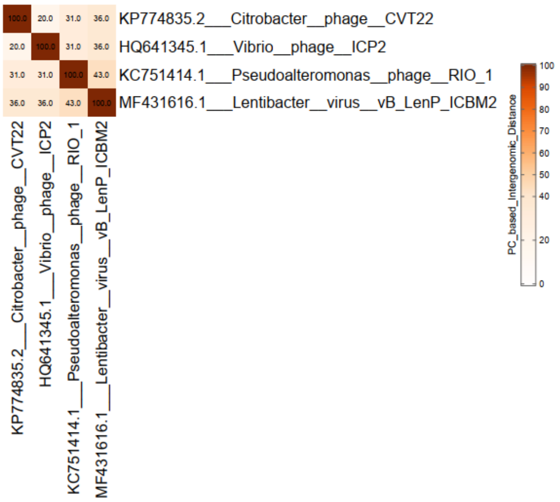

Figure 15: Step 3A - data-visualization sub-step.

Step 4A – splitting the tree into viral genome clusters (VGCs) and calculating statistics

A hierarchical tree that groups viral genomes based on their pairwise intergenomic distance can be further used to produce smaller viral genome clusters (VGCs), corresponding to different clades in the tree. This splitting into VGCs is performed in step 4A (Figure 16) and is controlled by the "clustering distance" parameter, which represents the minimum intergenomic distance two genomes should have to be placed into the same VGC.

An example is provided in Figure 17 to Figure 20**Error! Reference source not found.**. A test dataset (named here TESTA) was inputted in VirClust and a hierarchical tree (Figure 17) based on pairwise intergenomic distances was calculated in step 3A. For visualization purposes, the intergenomic distances have been transformed into intergenomic similarities ("1 – intergenomic distance") and plotted as a heatmap in Figure 18. The tree in Figure 17 can be split into several clusters, depending on the intergenomic distance used. For example, when using an intergenomic distance of 0.9, which is the equivalent to an intergenomic similarity of 0.1, three VGCs result (Figure 19). Checking Figure 18, we can see that the intergenomic similarities of the genome pairs in each of the three clusters are above 0.1. When using an intergenomic distance of 0.71, which is the equivalent of an intergenomic similarity of 0.29, the tree is split into 5 clusters Figure 20.

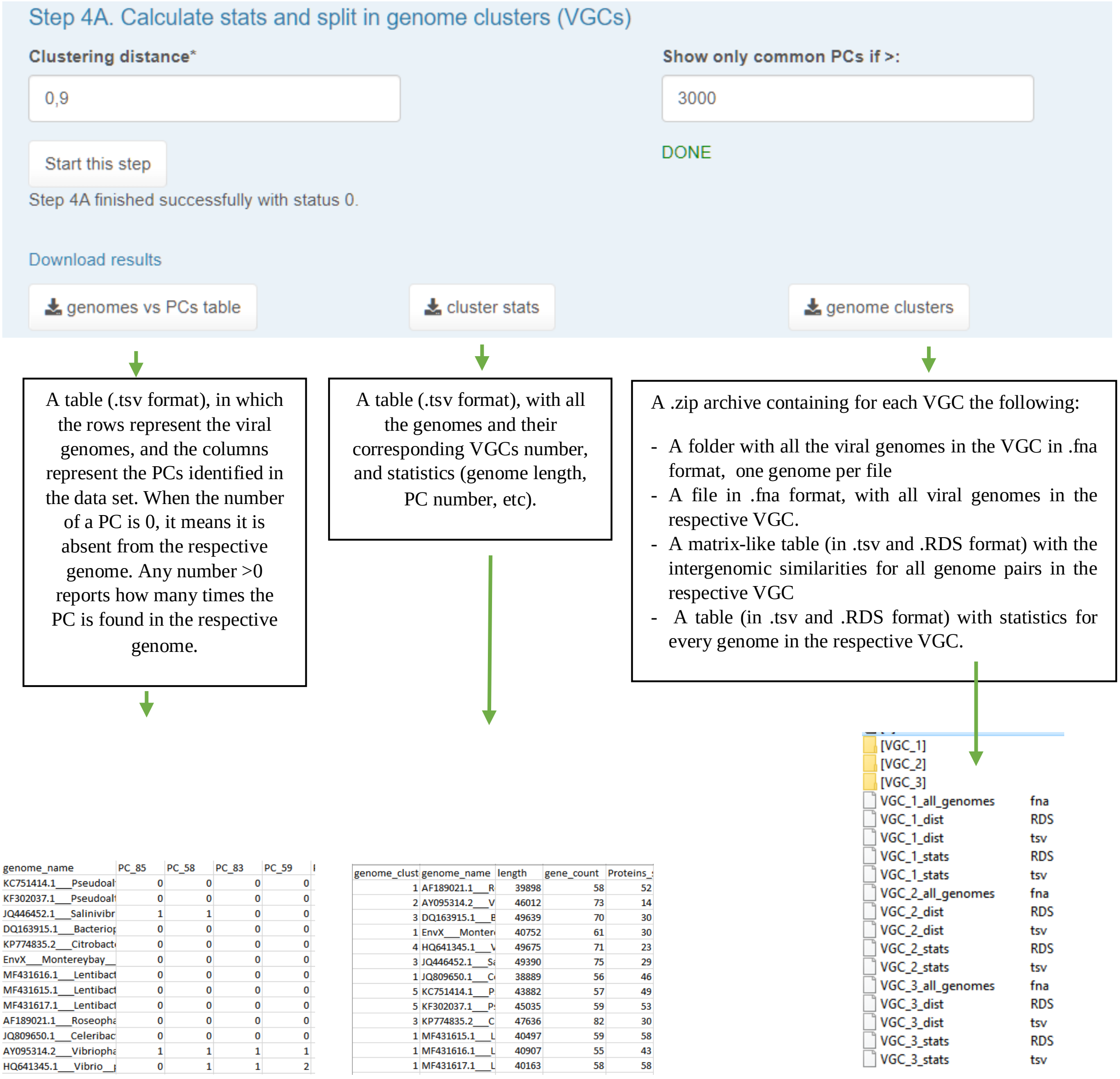

Figure 16: Step 4A – splitting the genome tree into smaller clusters and calculating statistics for each viral genome. The outputs the user can download are depicted in the lower part of the figure.

The outputs the user can download (Figure 16) are the following:

1. A table (.tsv format), in which the rows represent the viral genomes, and the columns represent the PCs identified in the data set.
2. A table (.tsv format), with all the genomes and their corresponding VGCs number, and statistics (genome length, PC number, etc).
3. A .zip archive containing for each VGC the following:
   - A folder with all the viral genomes in the VGC in .fna format, one genome per file
   - A file in .fna format, with all viral genomes in the respective VGC.
   - A matrix-like table (in .tsv and .RDS format) with the intergenomic similarities for all genome pairs in the respective VGC
   - A table (in .tsv and .RDS format) with statistics for every genome in the respective VGC.

   In the above .zip archive, the input genomes are returned to the user into files/folders corresponding to each VGC. This is very useful when working with input datasets consisting of high genome numbers, because the corresponding heatmaps from steps 3A and 4A can be very large, and thus, details about particular genome groups can be difficult to see. In such

cases, the genome files corresponding to each VGC can be used to create new VirClust projects, one per each VGC of interest. This would enable the generation of smaller heatmaps, which are easier to read and are also more suited for publication purposes.

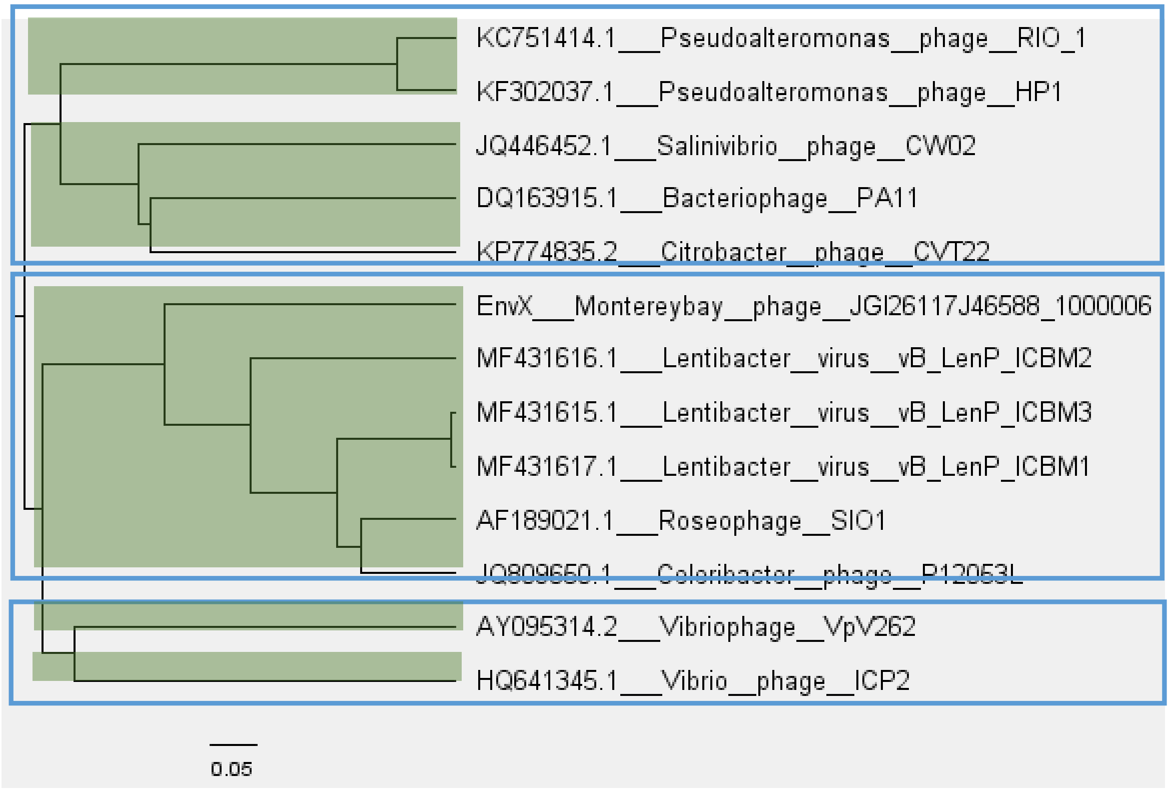

Figure 17: TESTA dataset – PC-based hierarchical tree generated by VirClust in step 4A and visualized with FigTree. When using an intergenomic distance of 0.9, the tree is cut in 3 VGCs (see *Figure 19*), marked in blue rectangles here. When using an intergenomic distance of 0.71, the tree is cut in 5 VGCs (see *Figure 20*), marked in green rectangles here.

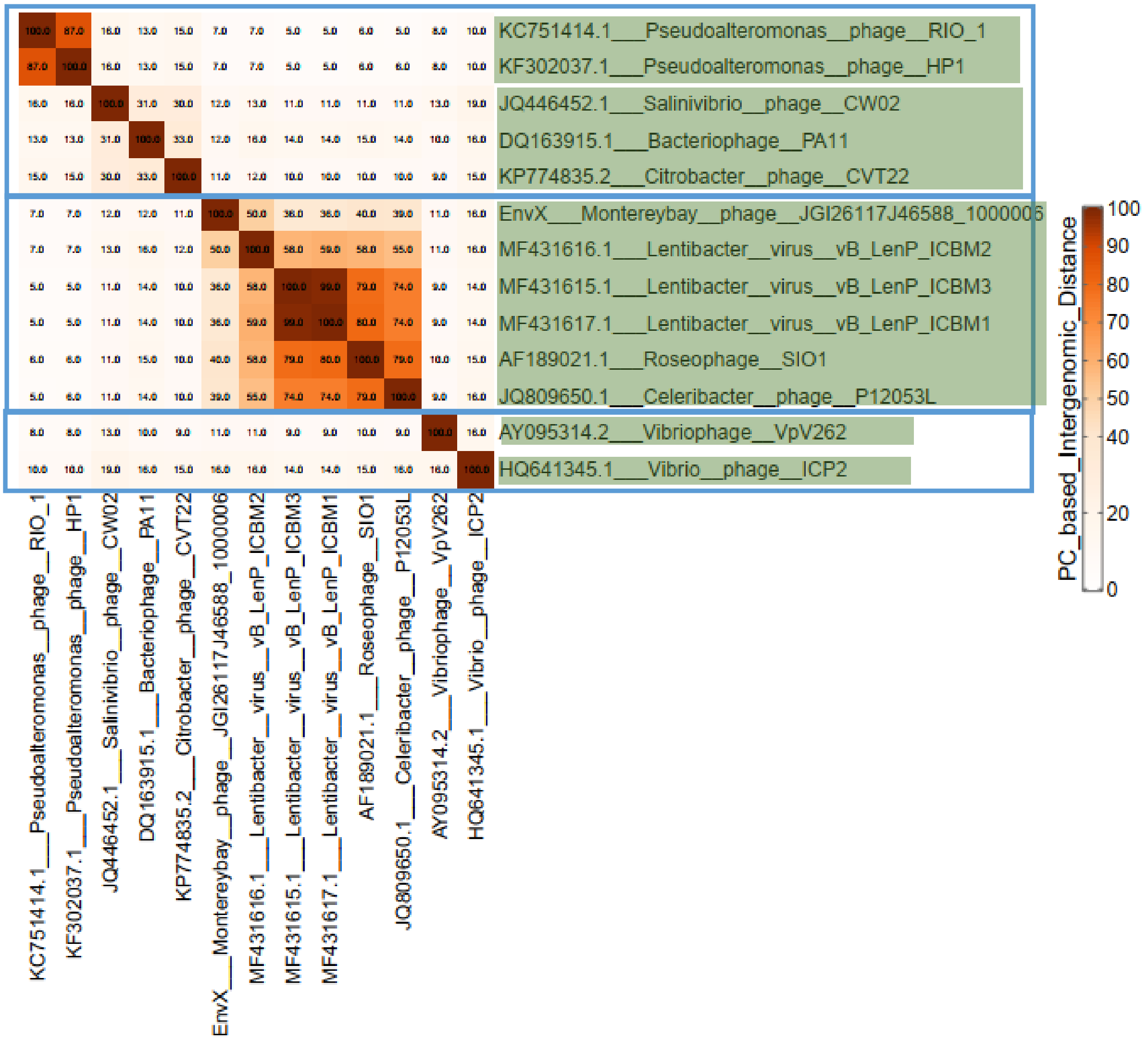

Figure 18: TESTA dataset – heatmap of the intergenomic similarities ("1 - intergenomic distance") on which the tree in Figure 17 is based. When using an intergenomic distance of 0.9, the tree is cut in 3 VGCs (see *Figure 19*), marked in blue rectangles here. When using an intergenomic distance of 0.71, the tree is cut in 5 VGCs (see *Figure 20*), marked in green rectangles here.

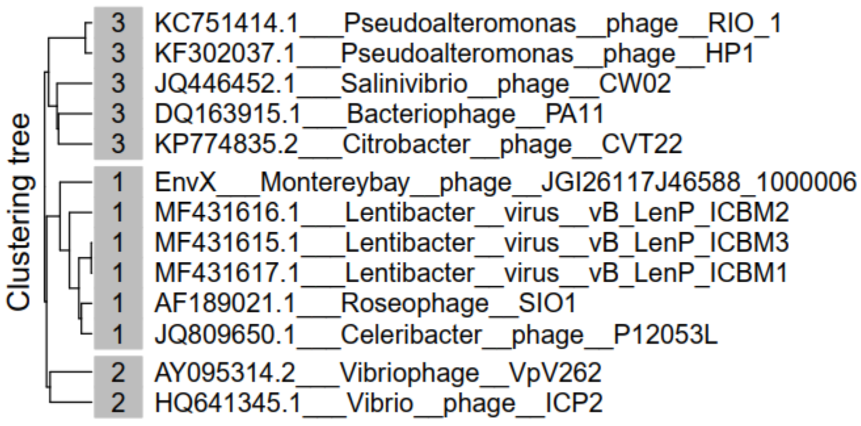

Figure 19: TESTA dataset - Hierarchical tree split at a 0.9 intergenomic distance. There are three VGCs, labeled as 1-3 in the grey column next to the tree.

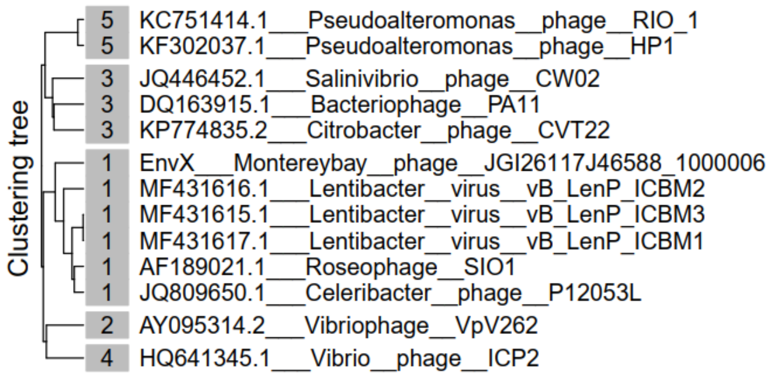

Figure 20: TESTA dataset - Hierarchical tree split at a 0.71 intergenomic distance. There are five VGCs, labeled as 1-5 in the grey column next to the tree.

Data visualization sub-step "Output genome clustering PDF"

Step 4A includes also a data-visualization sub-step, labeled "Output genome clustering PDF". Here, the user can generate and download a complex visualization (.PDF format) of the hierarchical clustering tree, the distribution of PCs in the viral genomes, and various genome statistics.

The components of the PDF are the following (Figure 21 panel B):

1. The clustering tree
2. The silhouette width
3. The VGC designation
4. A heatmap illustrating the PC distribution in the viral genomes. In this heatmap, the rows represent the viral genomes, and the columns the PCs. The absence of a PC in a viral genome is signaled by the white color in the heatmap. Any other color than white signifies that the PC is present in the respective viral genome, and it encodes the number of PC copies per genome.
5. Several genome-based statistics
   - *genome length*
   - *the proportion of PCs shared with any other viral genomes in the data set*
   - *the proportion of PCs shared with other genomes in its own VGC (regardless if those PCs are shared outside its own VGC or not)*
   - *the proportion of PCs shared only with genomes in its own VGC*
   - *the proportion of PCs shared with genomes outside its own VGC (regardless if those PCs are shared within its own VGC or not)*
   - *the proportion of PCs shared only with genomes outside its own VGC.*

From the web interface, the user can select to display in the PDF all five of the above components, or only some of them (Figure 21, panel A). For example, in Figure 20, only the tree, the silhouette width, and the corresponding genome names are shown. In Figure 21 (see panels A and B), all components are shown. Other formatting features that can be customized are:

- *tree width* – the width of the tree, proportional to the heatmap
- *width of the VGC ID column* – controls the width of the rectangle on which the VGC labels are displayed
- *width of the silhouette column*
- *width of the protein stats* – controls the width of all protein stats, proportional to the heatmap
- *font stats name* – controls the font size for the names of each of the statistics

- *font stats axis* – controls the font soze for the labels of each statistics X axis
- *column width (inches)* – gives the size of a PC column in the heatmap
- *font PC names* – controls the font size of the PC names displayed on the X-axis of the heatmap
- *font genomes/VGCs Ids* – controls the font size for the VGC labels and for the genome names
- *legend font* and *legend label font* – control the font size of the legend title and labels
- *legend height* and *legend width* control the height and width of the legend
- *show only common PCs if >* - controls which PCs are displayed in the heatmap. When the number of total PCs (excluding singletons) in the data set is bigger that the given value (default 3000), then only the columns corresponding to the most common P(SS)Cs (found in 75% of the genomes of the genomes from each genome cluster) will be plotted. This option is available from the main step 4A, and has to be given before the calculations performed in 4A.

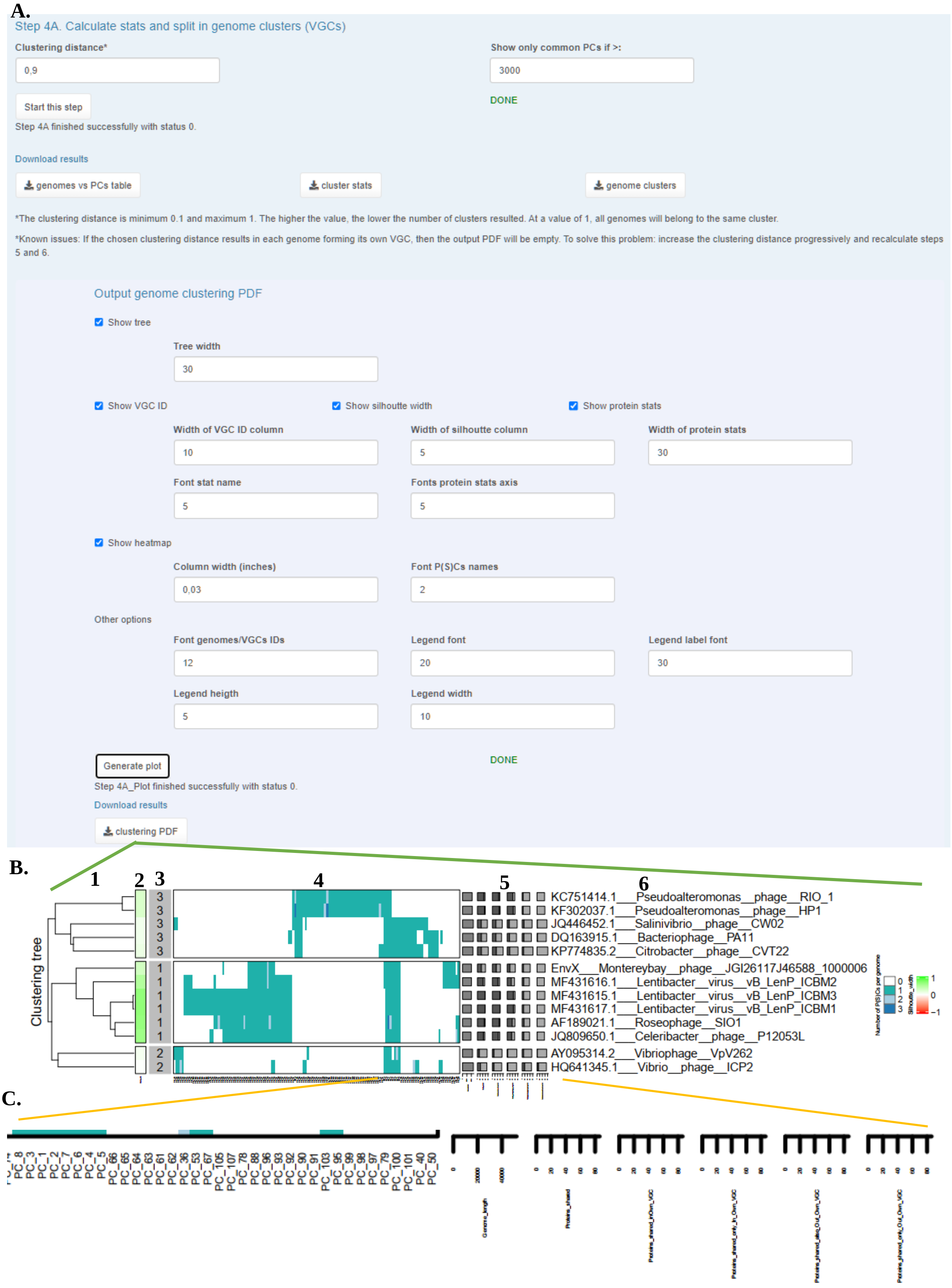

Figure 21: Step 4A - data-visualization sub-step (panel A), and an example of the generated PDF (panel B). To visualize details, for example the name of the PCs in the heatmap, the zoom-in function of the PDF viewer can be used (panel C). Panel B: 1. Clustering tree; 2. Silhoutte width; 3. VGC label; 4. Heatmap of the PC distribution in the viral genomes; 5. Genome statistics; and 6. Genome names.

Branch A – Core proteins module

Step 5A – Calculation of core proteins for each VGC, based on their PC content

The core proteins represent a related group of proteins found in all genomes from a dataset. VirClust considers as related all proteins in a PC (or in a PSC/PSSC, in the Branches B/C). Therefore, to be considered as belonging to the core, a PC needs to be found in all genomes from a dataset. For the calculation of the core proteins, VirClust considers all VGCs generated at step 4A as individual datasets. Therefore, VirClust calculates core proteins separately for each VGC. If the user wants to calculate the core proteins for the complete dataset inputted in VirClust, then she/he should set in step 4A the clustering distance to 1. This will ensure that all genomes in the input dataset will be clustered in a single VGC. Thus, in step 5A, core proteins will be searched for the complete dataset.

There are several outputs the user can download from step 5A, all found in a single .zip archive. For a detailed description, see Figure 22.

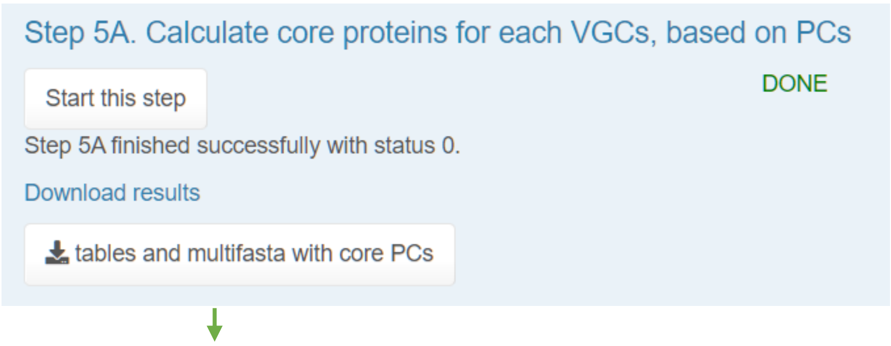

The output is a .zip archive containing for each VGCs the follwing:

- A table (.tsv and .RDS format) with each genome in the respective VGC, and their core genes and proteins.
- A .faa file with all core proteins for the respective VGC, labeled with their VirClust project protein ID
- A .faa file with all core proteins for the respective VGC, with a label composed of their PC number, genome name and gene number

Additionally, all core proteins from all VGC can be found in the for “all_proteins_indiv” or in the multifasta file “core_prots_for_annots_all”.

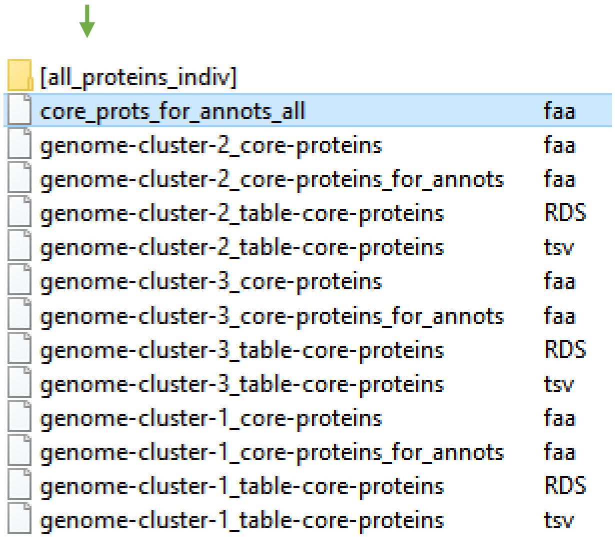

Figure 22: Step 5A – calculation of core proteins

Branches A, B and C – protein annotation modules

Step 6A/5B/5C – protein annotation

VirClust annotates proteins by searching for their homologs in the following databases: InterPro (Finn *et al.* 2017), pVOGs (Grazziotin *et al.* 2017), VOGDB (Kiening *et al.* 2019), Efam (Zayed *et al.* 2021), Efam-XC (Zayed *et al.* 2021) and NCBI-NR. After the homologs have been found, a filtering step keeps only the best matches for each protein. Then, VirClust appends the annotation results to the genome-protein table from step 2A/1B/1C (depending on which branch are the annotations performed). The web interface allows the user to query one database at a time (Figure 23). The user can choose to query all databases or only part of them. The annotation results from each database can be downloaded individually. Moreover, the annotations from the different databases can be merged and downloaded as one table.

In each branch, two different sets of proteins can be annotated: i) all proteins from all genomes; ii) only the core proteins for each VGC. For large genome datasets, the annotation of all proteins can take significant time and computational resources. Therefore, if the user needs only the core proteins, annotating them only can result in significant time savings.

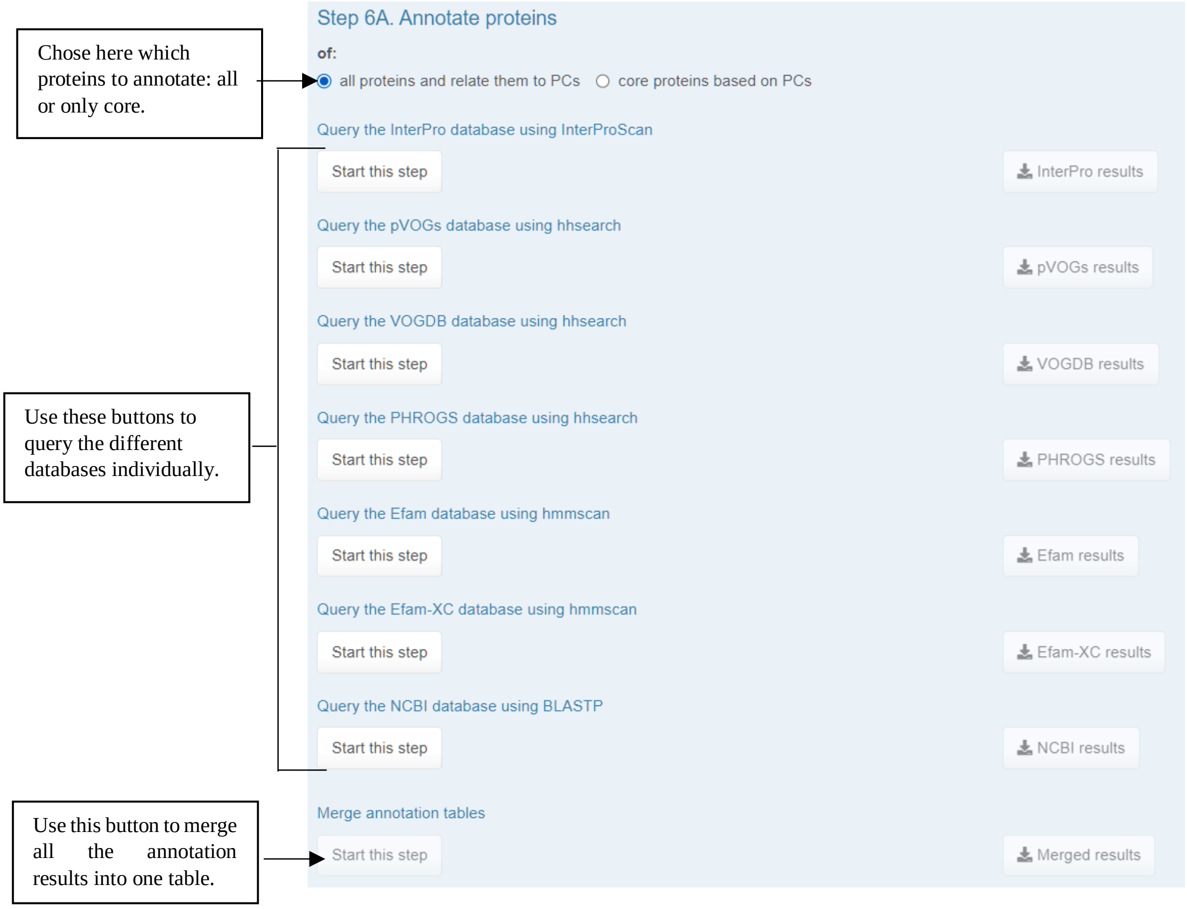

Figure 23: Step 6A/5B/5C – protein annotation.

Activation of the annotation buttons

To annotate all the proteins from a genome dataset, VirClust needs the proteins themselves, and their assignment to clusters (be it PCs, PSCs or PSSCs). Therefore, in any of the three branches, the buttons of the annotation module are activated only after the corresponding steps in the protein clustering module have been performed. For Branch A, the annotation buttons will be active only if

step 2A has been performed successfully. For Branch B, the annotations will be active only if step 1B has been performed. And, for Branch C, the annotations will be active only if step 1C has been performed.

To annotate only the core proteins, VirClust needs to assign first the core proteins. Therefore, the annotation buttons for the "core proteins" option will become active only after step 5A (Branch A), step 4B (Branch B), or step 4C (Branch C) have been calculated.

Annotation of "all proteins"

Behind the scenes, the annotation process against a single database consists of two independent phases: i) searching the database for homologous proteins and finding the best match, and ii) adding the annotation results to the genome-protein table, which contains also the PCs, PSCs and PSSCs columns. When merging all annotations, the results from phase one for each database are taken and merged with the genome-protein table corresponding to the respective branch. For the "all proteins" dataset, the only difference between the genome-protein table between the three branches is which protein clustering columns are present: the PCs, the PSCs, or the PSSCs column. In Branch A, there will be a column assigning each protein to its PC. In Branch B, in addition to the PC column, one more column will be found – the one assigning proteins to their PSCs. And in Branch C, all three columns will be found – the one for PCs, for PSCs, and for PSSCs (the last being specific for Branch C). Obviously, the proteins themselves in the "all proteins" dataset are the same, regardless of the branch.

Therefore, when querying the individual databases with the "all proteins" dataset, the first phase gives the same results in any of the three Branches (A, B or C). It follows that, is sufficient to perform the annotations against the individual databases only in one branch (regardless if its A, B or C) and then perform the merging of all annotations in the branch of interest (e.g Branch B, if one is interested in having the PCs and PSCs in the results table).

Annotation of "core proteins"

The core proteins can be different between the three branches. Therefore, the user needs to calculate them separately for each of the A, B or C branches.

The final assignment of protein annotations

VirClust returns a table summarizing the search results for all queried databases. Because the results between the databases can be somehow different, the user should validate the annotations and decide on a single annotation for each protein.

Considering that the proteins grouped in a PC, PSC or PSSC should have the same function, sorting the rows in the results table based on the protein assignment to PCs, PSCs or PSSCs has several benefits:

- It speeds up the process – many, if not all proteins in a P(SSC) will have the same results in the individual annotations. Once the final annotation for one protein has been decided, it can be easily transferred to the other proteins in the same PSSC.
- It allows the annotation of proteins that returned no homologs in any of the annotation databases. As long as these proteins belong to a P(SS)C, and some other proteins in the P(SS)C have annotations, they can be similarly annotated. Care should be taken in this case with multidomain proteins. If during the protein clustering steps, the thresholds for coverage are too relaxed, then it can lead to the clumping of different protein domains into the same cluster. This is illustrated in Figure 24.

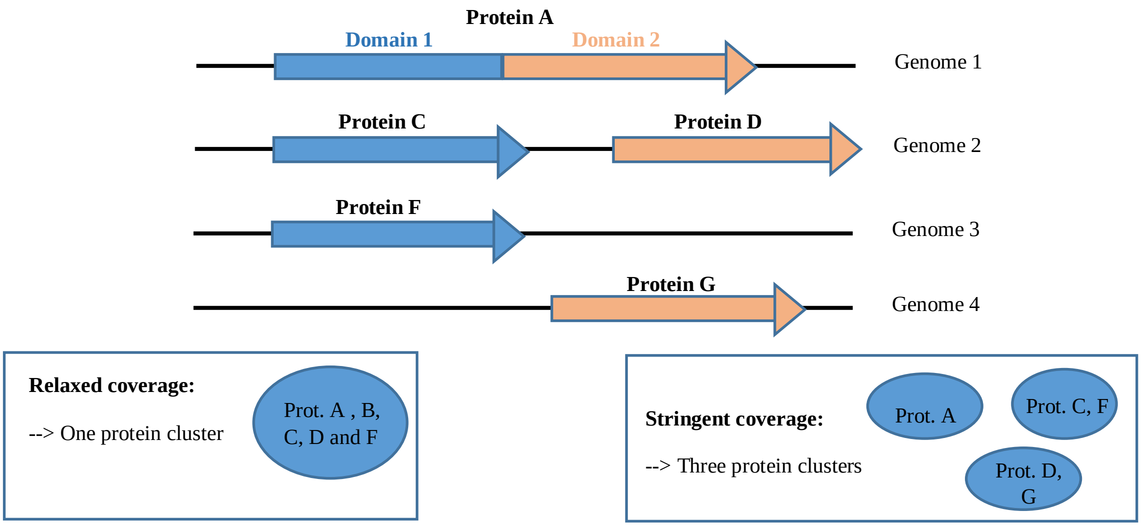

Figure 24: An example of multidomain proteins and assignment to protein clusters under different coverage thresholds. Under relaxed coverage paramters, all proteins end up in the same cluster. Under stringent paramters, the proteins end up in different clusters, depending on the domains they contain.

Branch B – Protein clustering module

Step 1B – from protein clusters to protein superclusters

In step 1B, the PCs from step 2A are grouped further into protein superclusters (PSCs), based on the similarities of their HMM profiles (Figure 25). To achieve that, VirClust is first calculating a multiple alignment for each PC, and then is calculating its HMM profile. Afterwards, it compares the HMM profiles of all PCs and, based on this comparison, it is grouping the PCs into PSCs.

The grouping into PSCs depends on the : i) the value used as proxy for the similarity of two HMM profiles in a query-subject result pair; and ii) the filtering thresholds for the query-subject pairs, which control which of the pairs resulting from a HMM comparisons are representing truly similar PCs (and will be kept for clustering) and which not (and will be removed before clustering).

Similar to the step 2A, four values can be used as proxy for the similarity for each query-subject PC pairs: i) the evalue; ii) the log-evalue; iii) the bitscore and iv) the normalized bitscore.

The query-subject PC pairs are filtered using two conditionals:

1. The first conditional is based on their probability and their coverage: all PC pairs with a probability AND coverage bigger (or equal) than the given values will be kept. The defaults for this step are probability >= 90 and coverage >= 50.
2. The second conditional is based on their probability, coverage and alignment length: all PC pairs with a probability, coverage AND alignment length larger (or equal) than the given values will be removed. The defaults for this step are probability >= 99, coverage >= 20 alignment length >= 100. This step allows the catching of HMM pairs with short regions of similarity, but of high significance.

   The two conditionals work togheter with an "OR" logic. That is, a PC pair will be kept if its matching either the first conditional or the second conditional. For the above examples, it means that all PC pairs having a probability >= 90 and coverage >=50 OR a probability >= 99, coverage >= 20 alignment length >= 100 will be kept.

After calculating this step, the user can download: i) a genome-protein table similar to the one from steps 1A and 2A, to which a column with the corresponding PSCs for each protein has been added; and ii) a .zip archive with the multiple alignments of all PCs calculated at step 2A.

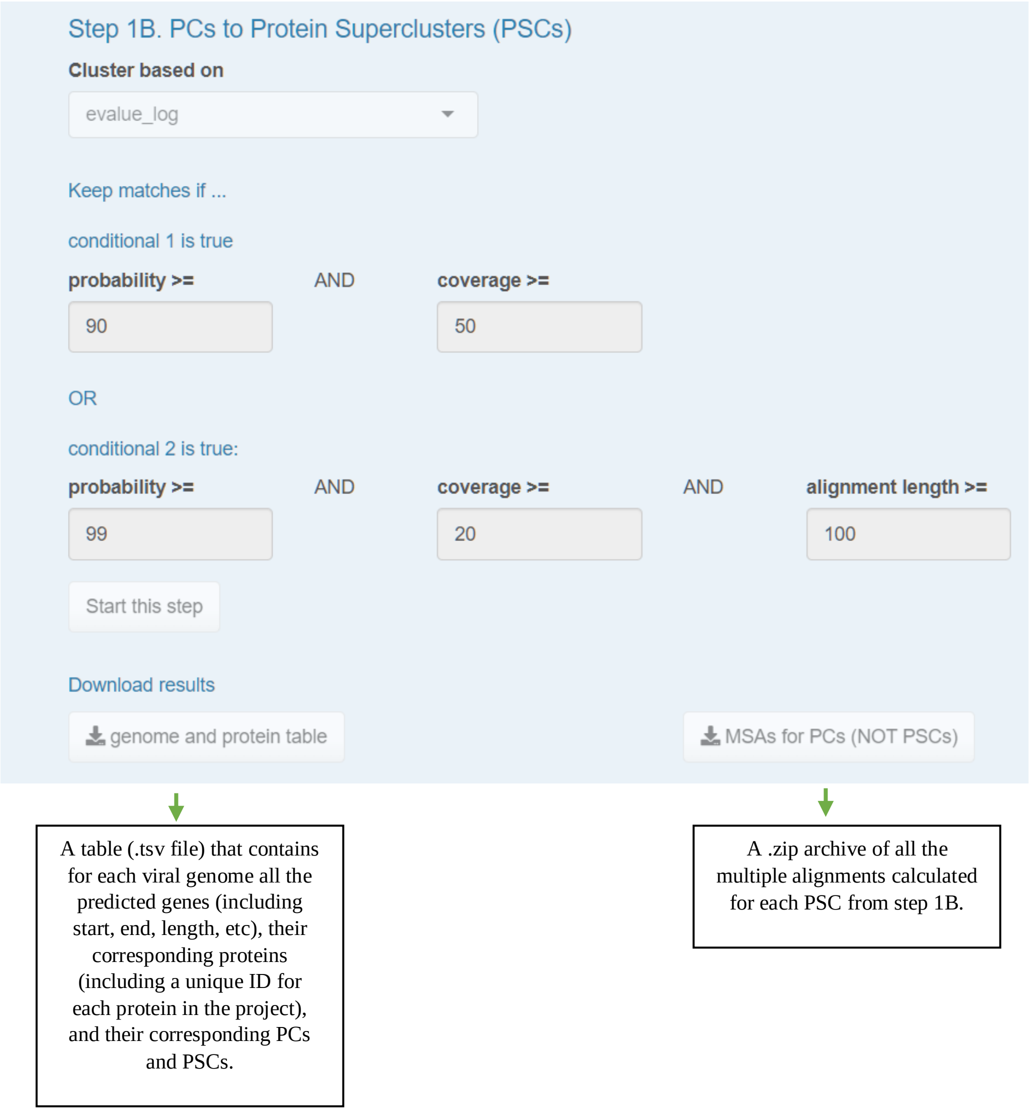

Figure 25: Step 1B - from PCs to PSCs.

Steps 2B, 3B, 4B and 5B

The rest of the steps are similar to the equivalent steps in Branch A (see Figure 7). The only difference is that they receive PSCs as input.

Branch C – Protein clustering module

Step 1C – from protein superclusters to protein super-superclusters

In Branch C, the protein superclusters (PSCs) generated in Branch B are grouped further into protein super-superclusters (PSSCs), based on their HMM profiles. The process is similar to that from step 1B (see Step 1B – from protein clusters to protein superclusters).

Steps 2C, 3C, 4C and 5C

The rest of the steps are similar to the equivalent steps in Branch A (see Figure 7). The only difference is that they receive PSSCs as input.

References

Finn, R.D., Attwood, T.K., Babbitt, P.C., Bateman, A., Bork, P., Bridge, A.J., Chang, H.-Y., Dosztányi, Z., El-Gebali, S., Fraser, M., Gough, J., Haft, D., Holliday, G.L., Huang, H., Huang, X., Letunic, I., Lopez, R., Lu, S., Marchler-Bauer, A., Mi, H., Mistry, J., Natale, D.A., Necci, M., Nuka, G., Orengo, C.A., Park, Y., Pesseat, S., Piovesan, D., Potter, S.C., Rawlings, N.D., Redaschi, N., Richardson, L., Rivoire, C., Sangrador-Vegas, A., Sigrist, C., Sillitoe, I., Smithers, B., Squizzato, S., Sutton, G., Thanki, N., Thomas, P.D., Tosatto, S.C.E., Wu, C.H., Xenarios, I., Yeh, L.-S., Young, S.-Y., and Mitchell, A.L. (2017) InterPro in 2017-beyond protein family and domain annotations. *Nucleic acids research, doi:* 10.1093/nar/gkw1107.

Grazziotin, A.L., Koonin, E.V., and Kristensen, D.M. (2017) Prokaryotic virus orthologous groups (pVOGs). A resource for comparative genomics and protein family annotation. *Nucleic acids research, doi:* 10.1093/nar/gkw975.

Kiening, M., Ochsenreiter, R., Hellinger, H.-J., Rattei, T., Hofacker, I., and Frishman, D. (2019) Conserved Secondary Structures in Viral mRNAs. *Viruses, doi:* 10.3390/v11050401.

Zayed, A.A., Lücking, D., Mohssen, M., Cronin, D., Bolduc, B., Gregory, A.C., Hargreaves, K.R., Piehowski, P.D., White, R.A., Huang, E.L., Adkins, J.N., Roux, S., Moraru, C., and Sullivan, M.B. (2021) efam: an expanded, metaproteome-supported HMM profile database of viral protein families. *Bioinformatics (Oxford, England), doi:* 10.1093/bioinformatics/btab451.
