# Supplementary material for: VirClust—A Tool for Hierarchical Clustering, Core Protein Detection and Annotation of (Prokaryotic) Viruses
Source: Viruses. 2023 Apr 19;15(4):1007. doi: 10.3390/v15041007 (PMC10143988; doi:10.3390/v15041007)
Supplement: Supplementary file 1 [file viruses-15-01007-s001.zip › SI_File3_VirClust-v2_manual_standalone.pdf]

VirClust v2 stand-alone – user manual

08.02.2023

Developer: Cristina Moraru

email liliana.cristina.moraru@uni-oldenburg.de

There are two versions of this manual: a PDF and a txt version (available from the command line). I recommend using preferably the PDF version, found here virclust.icbm.de.

Pre-requisites

- Install on your computer/server the Singularity v. 3.5.2 software (https://sylabs.io/)
- Download the VirClust singularity from virclust.icbm.de
- Unzip the folder (it contains the starting script and the singularity file)
- If you want to perform protein annotations, you need to download and install the databases (see below, section
- Location of databases for protein annotations)

To run VIRIDIC stand-alone

- Go to the folder with the VirClust singularity
- Type "./virclust.bash projdir=FOLDER_name infile= input_multi_genomes.fasta ……." to start VirClust
- Note the syntax above: parameter name followed by "=" and then parameter value. For example, in "infile= input_multi_genomes.fasta": "infile" is the parameter name, "input_multi_genomes.fasta" is the file name. When forming the command, DON'T use the double quotes around the parameters and their values.

To see the help file

- Go to the folder with the VirClust singularity
- Type "./virclust.bash help"

To see the version of VirClust

- Go to the folder with the VirClust singularity
- Type "./virclust.bash version"

Location of databases for protein annotations and parameters to set the databases

VirClust uses the following databases for protein annotations: Efam, Efam-XC, PHROGS, pVOGs, VOGDB, InterPro, or BLAST NR. These databases are not distributed with VirClust standalone. They should be downloaded and installed separately.

InterPro and BLAST NR databases

The InterPro database and InterProScan software should be downloaded and installed from here: https://www.ebi.ac.uk/interpro/download/InterProScan/ (comes together with the InterProScan software). VirClust singularity will connect to your InterProScan installation, as long as you provide the path toward its folder.

The BLAST NR database should be downloaded and installed as instructed here: https://www.ncbi.nlm.nih.gov/books/NBK569850/ .

Parameters to set the path toward the InterPro and BLAST NR databases

interproscan	represents the path toward the folder where InterProScan is installed on your system
blastdb	represents the path toward the folder where the BLAST NR database is found on your system

Efam, Efam-XC, PHROGS, pVOGs and VOGDB databases

The Efam, Efam-XC, PHROGS, pVOGs, and VOGDB databases can be downloaded from the VirClust website (see Download section), in a format compatible with VirClust. After downloading, you should remove them from the .tar.gz archive and place them all in a subfolder named dbs/. VirClust will search for the Efam, Efam-XC, PHROGS, pVOGs, VOGDB, InterPro or BLAST NR databases in the folder with the VirClust singularity, in the dbs/ subfolder.
The folder structure and the names of the subfolders for each database should be as follows:

```
Folder_with_the_singularity/
    virclust_v2_singularity.simg
    virclust.bash
    + dbs/
        + Efam/
        + Efam_XC/
        + PHROGS/
        + pVOGs/
        + VOGDB/
```

Alternatively, the above databases can be installed in a folder of the user's choice. But all need to be found in the same folder, and they should be named as above.

In both cases, the folder containing the databases should be specified separately.

Parameter to set the path toward the folder containing the Efam, Efam_XC, PHROGS, pVOGs, and VOGDB databases

databases — represents the path toward the folder where Efam, Efam_XC, PHROGS, pVOGs, and VOGDB are installed on your system.

Parameters for VirClust stand-alone

Mandatory parameters

projdir — the folder where VirClust will save its output. If continue is "no" and the folder already exists, it will be overwritten.

infile — input a .fasta file containing all viral genomes to be analyzed.

***Individual steps** - which VirClust steps do you want to run?*

The steps in different branches can be run individually. If the chosen step depends on an earlier step, and this one has not been previously performed, it will be activated automatically by VirClust. For example, choosing *step2A=T* for a new VirClust project will automatically activate *step1A=T*. For projects which are continued (they need the *continue=yes* parameter), if step1A has been previously performed, it will not be activated. The only exception to this automatic activation rule is the merging annotations steps *step6AM*, *step5BM,* and *step5CM*. They each need at least one search in a database to have been previously performed (see Protein annotation module), but the searches will not be automatically activated. A visual representation of the VirClust workflow and the dependencies between the workflows can be found in Figure 1 in the PDF version of this manual (available on the virclust.icbm.de website).

By default, all steps are false. You need to activate (set to "T", that is true) at least one step so that VirClust can run. Multiple steps can be activated at the same time. Their dependencies will be automatically activated.

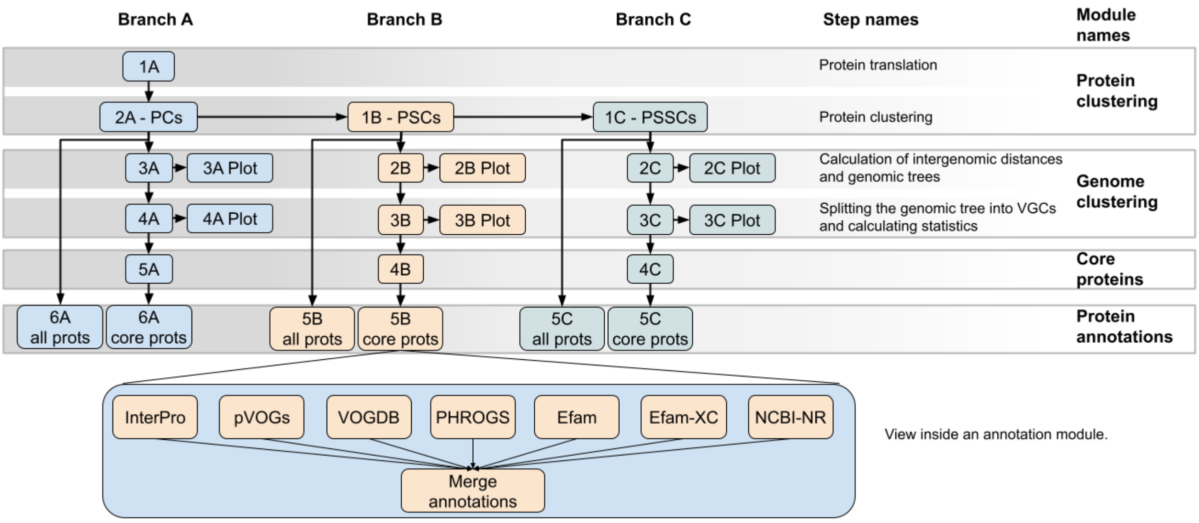

Figure 1: VirClust workflow and depndencies between the steps.

Branch A: Calculations based on protein-clusters.

Protein clustering module

step1A Performs ORF prediction and translation
Value (default): "T" (true), "F" (false)
Default: "F"

step2A Groups proteins into protein clusters (PCs)
Values: "T" (true), "F" (false)
Default: "F"

Genome clustering module

step3A Orders genomes hierarchically (calculates tree) based on their protein cluster content. The distance used for genome clustering takes into account the PC content of the genomes.
Values: "F" (true), "F" (false)
Default: "F"

step3A_Plot Outputs a PDF with an ordered heatmap of the PC-based intergenomic distances calculated at step 3A.

step4A Splits genomes in clusters based on the tree calculated at 3A and on the given clust_dist_a parameter.
Values: "T" (true), "F" (false)
Default: "F"

step4A_Plot Outputs a PDF of the genome clustering based on PCs.
Values: "T" (true), "F" (false)
Default: "F"

Core proteins module

step5A Calculates core proteins for each genome cluster based on PCs.
Values: "T" (true), "F" (false)
Default: "F"

Protein annotation module

step6AI Annotates proteins against the InterPro database (Finn *et al.* 2017) and relates them to their PCs.
Values: "T" (true), "F" (false)

Default: “F”

step6ApV Annotates proteins against the pVOGs database (Grazziotin *et al.* 2017) and relates them to their PCs.
Values: “T” (true), “F” (false)
Default: “F”

step6AVO Annotates proteins against the VOGDB (Virus Orthologous Groups) database (Kiening *et al.* 2019) and relates them to their PCs.
Values: “T” (true), “F” (false)
Default: “F”

step6APH Annotates proteins against the PHROGS database (Terzian *et al.* 2021) and relates them to their PCs.
Values: “T” (true), “F” (false)
Default: “F”

step6AE Annotates proteins against the Efam database (Zayed *et al.* 2021) and relates them to their PCs.
Values: “T” (true), “F” (false)
Default: “F”

step6AXC Annotates proteins against the Efam-XC database (Zayed *et al.* 2021) and relates them to their PCs.
Values: “T” (true), “F” (false)
Default: “F”

step6AN Annotates proteins against the BLAST NR database and relates them to their PCs.
Values: “T” (true), “F” (false)
Default: “F”

step6AM Merges the results from all searched annotation databases and relates them to their PCs.
Values: “T” (true), “F” (false)
Default: “F”

Branch B: Calculations based on protein superclusters.

Protein clustering module

step1B groups PCs into protein super-clusters (PSCs)
Values: “T” (true), “F” (false)
Default: “F”

Genome clustering module

step2B Orders genomes hierarchically (calculate tree) based on their protein super-cluster content.
Values: “T” (true), “F” (false)
Default: “F”

step2B_plot Outputs a PDF with an ordered heatmap of the PC-based intergenomic distances calculated at step 2B.

step3B Splits genomes in clusters based on the tree calculated at 2B and on the given clust_dist_b parameter.
Values: “T” (true), “F” (false)
Default: “F”

step3B_Plot Outputs a PDF of the genome clustering based on PSCs.
Values: “T” (true), “F” (false)
Default: “F”

step4B Calculates core proteins for each genome cluster based on PSCs.
Values: “T” (true), “F” (false)
Default: “F”

Core proteins module

step5B Annotates proteins and relates them to their PSCs.

Values: “T” (true), “F” (false)
Default: “F”

Protein annotation module

step5BI Annotates proteins against the InterPro database (Finn *et al.* 2017) and relates them to their PSCs.
Values: “T” (true), “F” (false)
Default: “F”

step5BpV Annotates proteins against the pVOGs database (Grazziotin *et al.* 2017) and relates them to their PSCs.
Values: “T” (true), “F” (false)
Default: “F”

step5BVO Annotates proteins against the VOGDB (Virus Orthologous Groups) database (Kiening *et al.* 2019) and relates them to their PSCs.
Values: “T” (true), “F” (false)
Default: “F”

step5BPH Annotates proteins against the PHROGS database (Terzian *et al.* 2021) and relates them to their PSCs.
Values: “T” (true), “F” (false)
Default: “F”

step5BE Annotates proteins against the Efam database (Zayed *et al.* 2021) and relates them to their PSCs.
Values: “T” (true), “F” (false)
Default: “F”

step5BXC Annotates proteins against the Efam-XC database (Zayed *et al.* 2021) and relates them to their PSCs.
Values: “T” (true), “F” (false)
Default: “F”

step5BN Annotates proteins against the BLAST NR database and relates them to their PSCs.
Values: “T” (true), “F” (false)
Default: “F”

step5BM Merges the results from all searched annotation databases and relates them to their PSCs.
Values: “T” (true), “F” (false)
Default: “F”

Branch C: Calculations based on protein super-superclusters.

Protein clustering module

step1C groups PSCs into protein super-clusters (PSSCs)
Values: “T” (true), “F” (false)
Default: “F”

Genome clustering module

step2C Orders genomes hierarchically (calculates tree) based on their protein super-supercluster content. The distance used for genome clustering takes into account the PSSC content of the genomes.
Values: “T” (true), “F” (false)
Default: “F”

step2C_plot Outputs a PDF with an ordered heatmap of the PC-based intergenomic distances calculated at step 2C.

step3C Splits genomes into clusters based on the tree calculated at 2C and on the given clust_dist_c parameter.
Values: “T” (true), “F” (false)
Default: “F”

step3C_Plot Outputs PDF of the genome clustering based on PSSCs.
Values: “T” (true), “F” (false)
Default: “F”

Core proteins module

step4C Calculates core proteins for each genome cluster based on PSSCs.
Values: “T” (true), “F” (false)
Default: “F”

Protein annotation module

step5CI Annotates proteins against the InterPro database (Finn *et al.* 2017) and relates them to their PSSCs.
Values: “T” (true), “F” (false)
Default: “F”

step5CpV Annotates proteins against the pVOGs database (Grazziotin *et al.* 2017) and relates them to their PSSCs.
Values: “T” (true), “F” (false)
Default: “F”

step5CVO Annotates proteins against the VOGDB (Virus Orthologous Groups) database (Kiening *et al.* 2019) and relates them to their PSSCs.
Values: “T” (true), “F” (false)
Default: “F”

step5CPH Annotates proteins against the PHROGS database (Terzian *et al.* 2021) and relates them to their PSSCs.
Values: “T” (true), “F” (false)
Default: “F”

step5CE Annotates proteins against the Efam database (Zayed *et al.* 2021) and relates them to their PSSCs.
Values: “T” (true), “F” (false)
Default: “F”

step5CXC Annotates proteins against the Efam-XC database (Zayed *et al.* 2021) and relates them to their PSSCs.
Values: “T” (true), “F” (false)
Default: “F”

step5CN Annotates proteins against the BLAST NR database and relates them to their PSSCs.
Values: “T” (true), “F” (false)
Default: “F”

step5CM Merges the results from all searched annotation databases and relates them to their PSSCs.
Values: “T” (true), “F” (false)
Default: “F”

Optional parameters

General parameters

cpu sets the number of cores to be used by those steps in VirClust doing multithreading.
Value: an integer, >1
Default: half of the cores in the machine on which VirClust is running

continue Should VirClust continue from previously computed steps? It works only if at least one step (i.e. step1A) was previously calculated. If continue is “yes”, and a certain step is selected, all the previous results of the respective and any following steps that depend on it are removed and recalculated. For example, if continue=yes and step2A=T, all results from steps >= 2A will be removed and recalculated. See “**Individual steps -** which VirClust steps do you want to run?” Section.
Options: “no”, “yes”.

Default: “yes”.

Options for step 1A

gene_code chooses the genetic code to be used when translating the genes to proteins.
Options: any number between 1 and 26 representing a valid genetic code
Default: 11

Options for step 2A– grouping the proteins into protein clusters, based on BLASTp searches

clust_PC chooses based on which features should the proteins be clustered in PCs.
Options: “evalue”, “evalue_log”, “bitscore” or “norm_bitscore”
Default: “evalue_log”

eval_PC Which e-value threshold should be used to filter out matches from the reciprocal BLASTP? Matches with an e-value bigger than eval_PC will be removed.
Options: numeric value between 0 and 0.01.
Default: 0.00001

bitsc_PC Which bitscore threshold should be used to filter out matches from the reciprocal BLASTP? Matches with a bitscore smaller than bitsc_PC will be removed.
Options: integer value >= 20.
Default: 50

cov_PC Which coverage (subject and query) threshold should be used to filter out matches from the reciprocal BLASTP? Matches with a coverage smaller than cov_PC will be removed.
Options: integer value between 0 and 100.
Default: 0 (coverage is not considered)

Options for step 1B – grouping the proteins clusters into protein superclusters, based on HMM profiles

prob1_PSC Minimum probability for a hit to be considered when performing protein clustering based on reciprocal HMM searches. It applies to the first hit filtering step.
Options: integer between 1 and 100.
Default: 90

prob2_PSC Minimum probability for a hit to be considered when performing PC clustering based on reciprocal HMM searches. It applies to the second hit filtering step.
Options: integer between 1 and 100.
Default: 99

cov1_PSC Minimum query and subject (template) coverage for a hit to be considered when performing PC clustering based on reciprocal HMM searches. It applies to the first hit filtering step.
Options: integer between 1 and 100.
Default: 50

cov2_PSC Minimum query and subject (template) coverage for a hit to be considered when performing PC clustering based on reciprocal HMM searches. It applies to the second hit filtering step.
Options: integer between 1 and 100.

Default: 20

alig_PSC Minimum alignment length for a hit to be considered when performing PC clustering based on reciprocal HMM searches. It applies to the second hit filtering step.

Options: integer >= 1.

Default: 100

Options for step 1C – grouping the proteins superclusters into protein super-superclusters, based on HMM profiles

prob1_PSSC Minimum probability for a hit to be considered when performing PSC clustering based on reciprocal HMM searches. It applies to the first hit filtering step.

Options: integer between 1 and 100.

Default: 90

prob2_PSSC Minimum probability for a hit to be considered when performing PSC clustering based on reciprocal HMM searches. It applies to the second hit filtering step.

Options: integer between 1 and 100.

Default: 99

cov1_PSSC Minimum query and subject (template) coverage for a hit to be considered when performing PSC clustering based on reciprocal HMM searches. It applies to the first hit filtering step.

Options: integer between 1 and 100.

Default: 50

cov2_PSSC Minimum query and subject (template) coverage for a hit to be considered when performing PSC clustering based on reciprocal HMM searches. It applies to the second hit filtering step.

Options: integer between 1 and 100.

Default: 20

alig_PSSC Minimum alignment length for a hit to be considered when performing PSC clustering based on reciprocal HMM searches. It applies to the second hit filtering step.

Options: integer between 1 and 100.

Default: 100

Options for the genome clustering steps 3A, 2B and 2C

aglom_a Agglomeration method to be used for the hierarchical clustering of the viral genomes in step 4a.

Options: “complete”, “average”

Default: “complete”

At the beginning of the clustering, each viral genome represents a cluster on its own, and the distance between clusters is given by the intergenomic distance calculated based on P(SS)Cs. The genomes with the smallest distance will be assigned to the same cluster. Then, the clusters with the shortest distance will be combined into larger clusters, in an iterative process, until only one big cluster is left. The way to calculate the shortest distance is given by the “complete” or “average” options. “Complete” refers to the “complete linkage” clustering, in which the distance between clusters represents the maximum distance of all genome pairs in the two clusters (one genome from each cluster). “Average” refers to the “unweighted pair group method with arithmetic mean” (UPGMA) linkage clustering, in which the distance between clusters represents the average distance between the genome pairs in the two clusters.

aglom_b Agglomeration method to be used for the hierarchical clustering of the viral genomes in step 4b.
Options: “complete”, “average”
Default: “complete”

aglom_c Agglomeration method to be used for the hierarchical clustering of the viral genomes in step 4c.
Options: “complete”, “average”
Default: “complete”

boot_pv_a if the genome clustering tree based on PCs should be calculated with or without bootstraping (very computationally intensive, could take very long times, disabled in the VirClust web if more than 50 genomes are given as input).
Options: “no”, “yes”.
Default: “no”.

bootstrap_no_a Number of bootstraps to be performed when calculating the genome clustering tree based on PCs. Needed only if boot_pv_a=yes.
Default: 1000
Options: >1

boot_pv_b if the genome clustering tree based on PSCs should be calculated with or without bootstraping (very computationally intensive, could take very long times, disabled in the VirClust web if more than 50 genomes are given as input).
Options: “no”, “yes”.
Default: “no”.

bootstrap_no_b Number of bootstraps to be performed when calculating the genome clustering tree based on PSCs. Needed only if boot_pv_b=yes.
Default: 1000
Options: >1

boot_pv_c if the genome clustering tree based on PSSCs should be calculated with or without bootstraping (very computationally intensive, could take very long times, disabled in the VirClust web if more than 50 genomes are given as input).
Options: “no”, “yes”.
Default: “no”.

bootstrap_no_c Number of bootstraps to be performed when calculating the genome clustering tree based on PSSCs. Needed only if boot_pv_b=yes.
Default: 1000
Options: >1

Options for the plotting steps 3A_Plot, 2B_Plot, and 2C_plot – generating an ordered heatmap of the intergenomic distances calculated at steps 3A, 2B or 2C

inc_fact_w_Pd The width and height of one heatmap cell, changing it will increase/decrease the heatmap width and height.
Default: 0.3.

font_row_Pd The font size of the row names (genome names).
Min value: 1.
Default: 12.

font_col_Pd The font size for the column names (genome names).
Min value: 1.

Default: 12.

font_cell_Pd The font size for the distance values that are displayed in each cell of the heatmap.
Min value: 1.
Default: 6.

lgd_width_Pd The width of the legend for the heatmap, normalized to the width of the heatmap.
Min value: 0.
Default: 15.

lgd_height_Pd The height of the legend for the heatmap (normalized to the height of the heatmap).
Min value: 0.
Default: 9.

lgd_font_Pd The size of the legend name.
Min value: 0.
Default: 5.

lgd_lab_font_Pd The font size for the labels of the legend.
Min value: 0.
Default: 4.

lgd_pos_Pd The position of the legend title.
Values: "topleft", "topcenter", "leftcenter", "lefttop", "leftcenter-rot", "lefttop-rot"
Default: "leftcenter-rot"

Options for steps 4A, 3B and 3C – calculating VGCs and their stats

clust_dist_a The intergenomic distances based on PCs to be used for splitting the viral genomes in clusters.
Options: 0.1 to 1.0. Note: at clust_dist=1.0 there will be only one single genome cluster (no splitting).
Default: “0.9”

clust_dist_b The intergenomic distances based on PSCs to be used for splitting the viral genomes in clusters.
Options: 0.1 to 1.0. Note: at clust_dist=1.0 there will be only one single genome cluster (no splitting).
Default: “0.9”

clust_dist_c The intergenomic distances based on PSSCs to be used for splitting the viral genomes in clusters.
Options: 0.1 to 1.0. Note: at clust_dist=1.0 there will be only one single genome cluster (no splitting).
Default: “0.9”

Option for the plotting steps 4A_Plot, 3B_Plot, and 3C_Plot

Tree parameters

show_tree if in the PDF the tree used to cluster the genomes should be plotted or not.
Options: “no”, “yes”.
Default: “yes”.

tree_width The width of the tree plot is relative to the heatmap width. (100 = the heatmap width, 50 = half of the heatmap width)
Default: 50.
Min value: 1.

show_clust_ID if the ID of the genome cluster should be shown.
Options: "no", "yes".
Default: "yes".
clustID_width The width of the cluster ID column is relative to the inc_fact_w.
Min value: 1.
Default: 5.

Heatmap parameters

show_heat If the heatmap with the distribution of the P(SS)Cs should be shown.
Options: "no", "yes".
Default: "yes".

inc_fact_w The width of one heatmap column, it will increase/decrease the heatmap width.
Default: 0.03.

max_cols_HT The maximum number of P(SS)C columns to be plotted in the heatmap. If the number of P(SS)Cs (excluding singletons) is bigger than max_col_HT, then only the columns corresponding to the most common P(SS)Cs (found in 75% of the genomes from each genome cluster) will be plotted. This option activates steps step4A, or step3B, or step3C.

font_row The font size for the row names (genome names) and the genome cluster IDs.
Min value: 1.
Default: 12.

font_col The font size for the column names (protein (super-)cluster IDs).
Min value: 1.
Default: 2.

Stats parameters

show_protein_stats If the stats for each genome should be plotted or not in the PDF.
Options: "no", "yes".
Default: "yes".

show_sil If the stat "silhouette width" should be displayed. If clust_dist_a/b is 1, then silhouette width will not be calculated.
Options: "no", "yes".
Default: "yes".

stats_width The total width of all the stats plot, it is relative to the heatmap width (100 = the heatmap width, 50 = half of the heatmap width).
Min value: 0.
Default: 40.

sil_stats_width The width of the silhouette stat is relative to inc_fact_w.
Min value: 1.

Default: 5.

stats_font The font size for the stat plots titles.
Min value: 1.
Default: 5.

stats_lab_font The font size for the axis labels of the stat plots.
Min value: 1.
Default: 5.

Heatmap legend parameters

lgd_width The width of the legend for the heatmap.
Min value: 0.
Default: 40.

lgd_height The height of the legend for the heatmap.
Min value: 0.
Default: 3.

lgd_font The size of the legend name.
Min value: 0.
Default: 30.

lgd_lab_font The font size for the labels of the legend.
Min value: 0.
Default: 15.

lgd_pos The position of the legend title.
Values: "topleft", "topcenter", "leftcenter", "lefttop", "leftcenter-rot", "lefttop-rot"
Default: "leftcenter-rot"

Options for steps 6A/5B/5C – protein annotations

If annotations are performed, the following parameters are mandatory:

interproscan represents the path toward the folder where InterProScan is installed on your system

blastdb represents the path toward the folder where the BLAST NR database is found on your system

databases represents the path toward the folder where Efam, Efam_XC, PHROGS, pVOGs, and VOGDB are installed on your system.

The following parameter is optional:

prot_type Selects which proteins should be annotated and to which tables should the annotation information be added. Considered only if steps 6A/5B/5C=T.
Choices:
"all_PCs", annotates all proteins and adds the annotations to the genome and protein tables containing the PC assignments.

"core_PCs", annotates the core proteins calculated based on PCs and adds the annotation information to the respective core protein tables

"all_PSCs", annotates all proteins and adds the annotations to the genome and protein tables containing the PC and PSC assignments.

"core_PSCs", annotates the core proteins calculated based on PSCs and adds the annotation information to the respective core protein tables

"all_PSSCs", annotates all proteins and adds the annotations to the genome and protein tables containing the PC, PSCs, and PSSC assignments.

"core_PSSCs", annotates the core proteins calculated based on PSCs and adds the annotation information to the respective core protein tables

Default: "all_PCs"

Options for VirClust's internal use:

- pc_type: to specify if steps 4-7 should be calculated based on PCs or PSCs

Usable outputs

Protein clustering module

Step 1A

- Folder *01/*
  - Subfolders:
    - *01_out_faa/*, contains the proteins for each genome (one .faa file per genome). The proteins are named in the format *genome-name_geneID*
    - *01_all_proteins_individual/*, contains all proteins from all the genomes, one protein per .faa file, labeled with their VirClust protein ID
  - Files:
    - *01_all_proteins.faa*, a multifasta file containing all proteins from all genomes, labeled with their VirClust protein ID (see the *01_genome_protDF_PCs.tsv/.RDS* file for further info about the proteins).
    - *01_genome_protDF_PCs.tsv/.RDS*, a table in .tsv and .RDS format, containing all the predicted genes (including start, end, length, etc) and their corresponding proteins for every viral genome.

Step 2A

- Folder *02/*
  - Files:
    - *02_04_genome_protDF_PCs.tsv/.RDS*, represents a table with all genes from all viral genomes (one gene per row), their genome location, their corresponding proteins (including the unique protein IDs) and the assigned PCs and PSCs.

Step 1B

- Folder *03/*
  - Files:
    - *03_09_genome_protDF_PCs_PSCs.tsv/RDS*, represents a table with all genes from all viral genomes (one gene per row), their genome location, their corresponding proteins (including the unique protein IDs), and the assigned PCs and PSCs.
  - Subfolder:

- *03_02_aligned_PCs/out/*, contains aligned multifasta files, one for each PC produced at step 2A

Step 1C

- Folder *03C/*
  - Files:
    - *03C_09_genome_protDF_PCs_PSCs.tsv/RDS*, represents a table with all genes from all viral genomes (one gene per row), their genome location, their corresponding proteins (including the unique protein IDs) and the assigned PCs, PSCs and PSSCs.
  - Subfolder:
    - *03C_02_aligned_PSCs/out/*, contains aligned multifasta files, one for each PSC produced at step 1B

Genome clustering module

Steps 3A/2B/2C

- Folders:
  - *04a-06a_genome_clustering_PC/04/* for step 3A
    - Files:
      - *MyDistPCs_MA.tsv/.RDS*, a table with the calculated intergenomic distances for each genome pair
      - *MyDistPCs_MA_ordered.tsv/.RDS*, a table with the calculated intergenomic distances for each genome pair. The genomes on the rows and columns are ordered based on their intergenomic distances.
      - *unord_hDF.RDS*, a table which PCs / PSCs are found in which viral genome, with the rows and the columns not particularly ordered.
      - *hc_tree.newick/hc.RDS* – hierarchical tree without bootstrapping
      - *pv_tree_bp.newick/pv.RDS* – hierarchical tree with bootstrapping
  - *04b-06b_genome_clustering_PSC/04/* for step 2B
    - Files: same as above
  - *04c-06c_genome_clustering_PSSC/04/* for step 2C
    - Files: same as above

Steps 3A_Plot/2B_Plot/2C_Plot

- Folders:
  - *04a-06a_genome_clustering_PC/04/* for step 3A
    - Files:
      - Dist_heatmap_PC_all_genomes.PDF, a PDF file with a clustered heatmap of the intergenomic distances between all genome pairs
  - *04b-06b_genome_clustering_PSC/04/* for step 2B
    - Files: same as above
  - *04c-06c_genome_clustering_PSSC/04/* for step 2C
    - Files: same as above

Step 4A/3B/3C

- Folders:
  - *04a-06a_genome_clustering_PC/05/* for step 4A
    - Files:
      - *virDF.tsv/.RDS*, contain the following per genome statistics:
        - length (genome length in bases)

- gene_count (total number of genes/proteins per genome)
- Single_Proteins (total number of proteins that belong to singletons, that is to P(SS)Cs that have only one protein and thus, are not shared with any other viral genome),
- Proteins_shared (total number of proteins found in P(SS)Cs shared with other viral genomes in the dataset)
- Proteins_shared_inOwn_GC (total number of proteins found in P(SS)Cs shared with viral genomes from the same genome cluster, regardless if they are shared with viral genomes from outside the genome cluster as well)
- Proteins_shared_only_In_Own_GC (total number of proteins found in P(SS)Cs shared exclusively with viral genomes only from the same genome cluster)
- Proteins_shared_also_Out_Own_GC (total number of proteins found in P(SS)Cs shared with viral genomes from other genome clusters, regardless if they are shared with viral genomes from the same genome cluster as well)
- Proteins_shared_only_Out_Own_GC (total number of proteins found in P(SS)Cs shared exclusively with viral genomes from other genome clusters)
- genome_cluster_ID
- silhouette_neighbour
- silhouette_width

- *ord_hDF.tsv/.RDS*, represents a table with witch which PCs / PSCs are found in which viral genome, with the rows ordered by viral genomes (based on the tree calculated at step 3A/2B/2C) and the columns by PCs/PSCs.

- *Subfolder:*
  - *VGCs_dist/* – contains data for each Viral Genome Clusters
    - Files
      - *VGC_#_all_genomes.fna* – multifasta genome files, one per VGC (# represents the number of the VGC), each containing all the viral genomes in the respective VGC.
      - *VGC_#_dist.RDS/.tsv* – a table with pairwise intergenomic distances for the respective VGC
      - *VGC_#_stats.RDS/.tsv* – a table with the stats (see virDF.tsv file above) for the respective VGC
    - Subfolders
      - */VGC_#* – folders, one per VGC (# represents the number of the VGC), each containing all individual files with the viral genomes from the respective VGC.

- *04b-06b_genome_clustering_PSC/05/* for step 3B
  - Files and subfolders: same as above
- *04c-06c_genome_clustering_PSSC/05/* for step 3C
  - Files and subfolders: same as above

Step 4A_Plot/3B_Plot/3C_Plot

- Folders:
  - *04a-06a_genome_clustering_PC/*
    - *File:*
      - *06-Heatmap_PC.PDF*, contains the integrated visualization of the genome clustering, including the clustering tree, the heatmap with the protein distribution in genomes, and various statistics
  - 04b-06b_genome_clustering_PSC/
    - *File:*

            - *06-Heatmap_PSC.PDF*, contains the integrated visualization of the genome clustering, including the clustering tree, the heatmap with the protein distribution in genomes, and various statistics
    - 04c-06c_genome_clustering_PSSC/
        - *File:*
            - *06-Heatmap_PSSC.PDF*, contains the integrated visualization of the genome clustering, including the clustering tree, the heatmap with the protein distribution in genomes, and various statistics

Core proteins module

Steps 5A/4B/4C

- Folders:
    - *07/core_a/* for step 5A
        - *Files:*
            - *core_prots_for_annots_all.faa* contains the core proteins from all VGCs, labeled with their unique VirClust protein ID.
            - *genome-cluster-#_core-proteins.faa* – one file for each genome cluster, containing all core proteins. The naming scheme of the proteins is: >P(SS)C-#__organisms_name_gene_#
            - *genome-cluster-#_core-proteins_for_annots.faa* – one file for each genome cluster, containing all core proteins. The proteins are labeled with their unique VirClust protein ID.
            - *genome-cluster-#_table-core-proteins.tsv/.RDS* – one file for each genome cluster, containing a table with all core genes from all viral genomes (one gene per row), their genome location, their corresponding proteins (including the unique protein IDs), and the assigned PCs and PSCs.
        - *Subfolders:*
            - /all_proteins_indiv – a folder containing all core proteins from all VGCs, labeled with their unique VirClust protein ID.
    - *07/core_b/* for step 4B
        - Files and subfolders: same as above
    - *07/core_c/* for step 4C
        - Files and subfolders: same as above

NOTE: No core P(SS)Cs are calculated for virus clusters with one single genome.

Protein annotation module

Steps 6A/5B/5C

- Folders:
    - *08_annots/* – for the annotation of all proteins (steps 6A/5B/5C)
        - *Subfolders:*
            - interproscan_out/
                - Files:
                    - *interpro_TB_joined.tsv/.RDS* – a table with the annotation results from the InterPro database, and proteins assigned to their respective P(SS)Cs
            - hhsearch_pVOGs/
                - Files:
                    - *hhsearch_pVOGs_TB_joined.tsv/.RDS* – a table with the annotation results from the pVOGs database, and proteins assigned to their respective P(SS)Cs
            - hhsearch_PHROGS/

- Files:
  - *hhsearch_PHROGS_TB_joined.tsv/.RDS* – a table with the annotation results from the PHROGS database, and proteins assigned to their respective P(SS)Cs
- hhsearch_VOGDB/
  - Files:
    - *hhsearch_VOGDB_TB_joined.tsv/.RDS* – a table with the annotation results from the VOGDB database, and proteins assigned to their respective P(SS)Cs
- hmmscan_Efam/
  - Files:
    - *hmmscan_Efam_joined.tsv/.RDS* – a table with the annotation results from the Efam database, and proteins assigned to their respective P(SS)Cs
- hmmscan_Efam_XC/
  - Files:
    - *hmmscan_Efam_XC_joined.tsv/.RDS* – a table with the annotation results from the Efam-XC database, and proteins assigned to their respective P(SS)Cs
- BlastP_NR/
  - Files:
    - *blastp_TB_joined.tsv/.RDS* – a table with the annotation results from the BLAST NR database, and proteins assigned to their respective P(SS)Cs
- all_outputs/
  - Files:
    - *annots_and_interpro_TB_P(SS)C.tsv/.RDS* – a table with the annotations results from all databases, and proteins assigned to their respective P(SS)Cs
    - *annots_TB_P(SS)C.tsv/.RDS* – a table with the annotations results from all databases, except InterPro, and proteins assigned to their respective P(SS)Cs

- 08_annots_core_a/ – for the annotation of core proteins in Branch A, step 6A
  - Files and subfolders: same as above, but only for core PCs
- 08_annots_core_b/ – for the annotation of core proteins in Branch B, step 5B
  - Files and subfolders: same as above, but only for core PSCs
- 08_annots_core_c/ – for the annotation of core proteins in Branch C, step 5C
  - Files and subfolders: same as above, but only for core PSSCs

Shiny specific options (not for the standalone user):

- shiny (if VirClust is run from shiny; options “no”, “yes”)
- multiF is “yes” when the input genomes are found in one multifasta file and “no” when the input genomes are found in multiple, single genome files. Only for the shiny version is possible to give multiple files. For the standalone version, all genomes need to be given in a single multifasta file. Options: “yes” (multifasta file), “no” (single genome files, but many of them).
- both .fasta or .fna input files are possible (as opposed to the command line version, where only .fasta are allowed as input)
- boot_pv_a or boot_pv_b is always “no” if more than 50 genomes are given as input. For more than 50 genomes these options are available in VirClust singularity.

References

Finn, R.D., Attwood, T.K., Babbitt, P.C., Bateman, A., Bork, P., Bridge, A.J., Chang, H.-Y., Dosztányi, Z., El-Gebali, S., Fraser, M., Gough, J., Haft, D., Holliday, G.L., Huang, H., Huang, X., Letunic, I., Lopez, R., Lu, S., Marchler-Bauer, A., Mi, H., Mistry, J., Natale, D.A., Necci, M., Nuka, G., Orengo, C.A., Park, Y., Pesseat, S., Piovesan, D., Potter, S.C., Rawlings, N.D., Redaschi, N., Richardson, L., Rivoire, C., Sangrador-Vegas, A., Sigrist, C., Sillitoe, I., Smithers, B., Squizzato, S., Sutton, G., Thanki, N., Thomas, P.D., Tosatto, S.C.E., Wu, C.H., Xenarios, I., Yeh, L.-S., Young, S.-Y., and Mitchell, A.L. (2017) InterPro in 2017-beyond protein family and domain annotations. *Nucleic acids research, doi:* 10.1093/nar/gkw1107.

Grazziotin, A.L., Koonin, E.V., and Kristensen, D.M. (2017) Prokaryotic virus orthologous groups (pVOGs). A resource for comparative genomics and protein family annotation. *Nucleic acids research, doi:* 10.1093/nar/gkw975.

Kiening, M., Ochsenreiter, R., Hellinger, H.-J., Rattei, T., Hofacker, I., and Frishman, D. (2019) Conserved Secondary Structures in Viral mRNAs. *Viruses, doi:* 10.3390/v11050401.

Terzian, P., Olo Ndela, E., Galiez, C., Lossouarn, J., Pérez Bucio, R.E., Mom, R., Toussaint, A., Petit, M.-A., and Enault, F. (2021) PHROG: families of prokaryotic virus proteins clustered using remote homology. *NAR genomics and bioinformatics, doi:* 10.1093/nargab/lqab067.

Zayed, A.A., Lücking, D., Mohssen, M., Cronin, D., Bolduc, B., Gregory, A.C., Hargreaves, K.R., Piehowski, P.D., White, R.A., Huang, E.L., Adkins, J.N., Roux, S., Moraru, C., and Sullivan, M.B. (2021) efam: an expanded, metaproteome-supported HMM profile database of viral protein families. *Bioinformatics (Oxford, England), doi:* 10.1093/bioinformatics/btab451.
