# Supplementary material for: VirClust—A Tool for Hierarchical Clustering, Core Protein Detection and Annotation of (Prokaryotic) Viruses
Source: Viruses. 2023 Apr 19;15(4):1007. doi: 10.3390/v15041007 (PMC10143988; doi:10.3390/v15041007)
Supplement: Supplementary file 1 [file viruses-15-01007-s001.zip › SI_file4_Crz_DB_interegenomic_distances_genomic_trees.pdf]

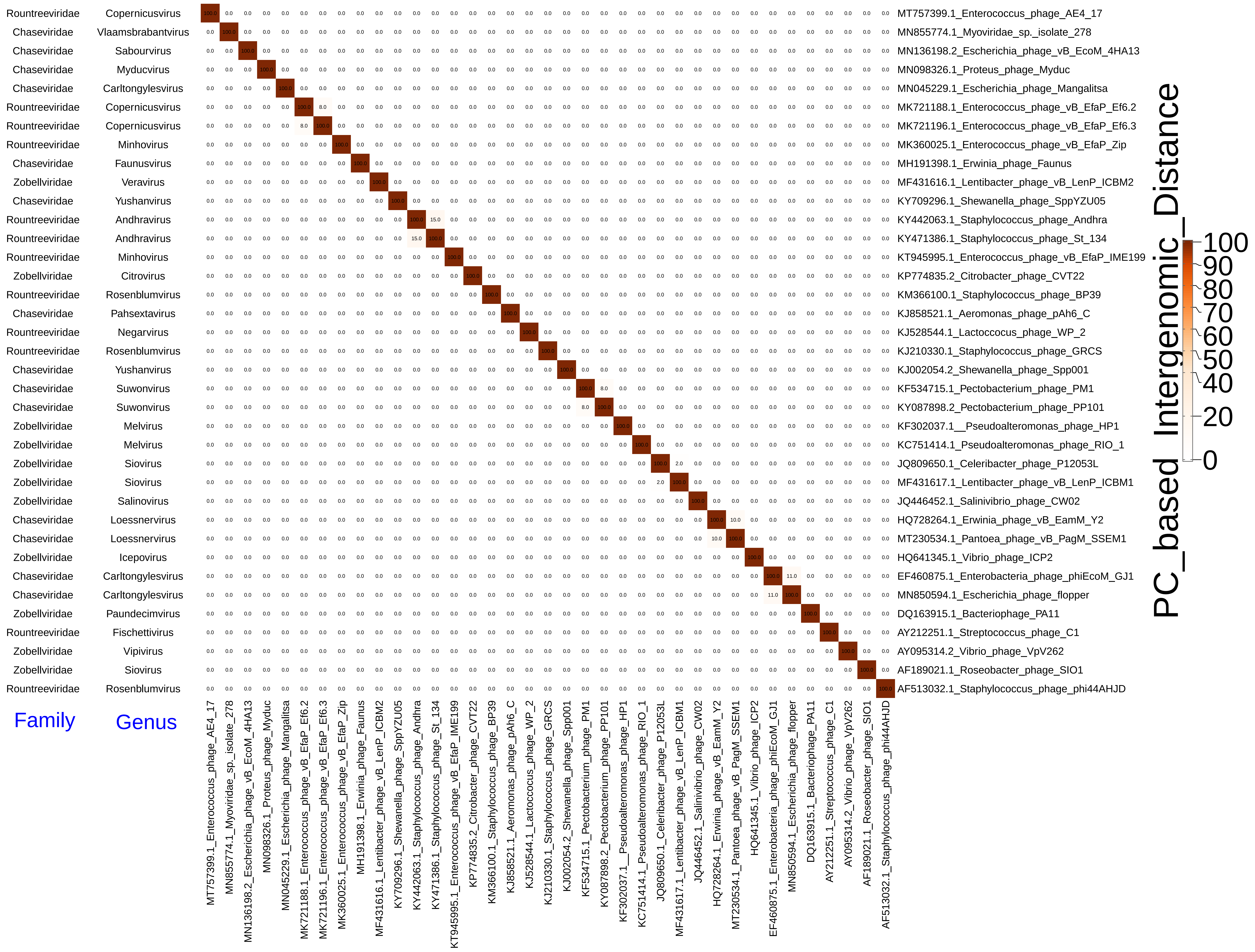

Fig. S1: Intergenomic distances for Crz_DB dataset, calculated with the following protein clustering parameters: coverage = 100%, % identity = 100%.

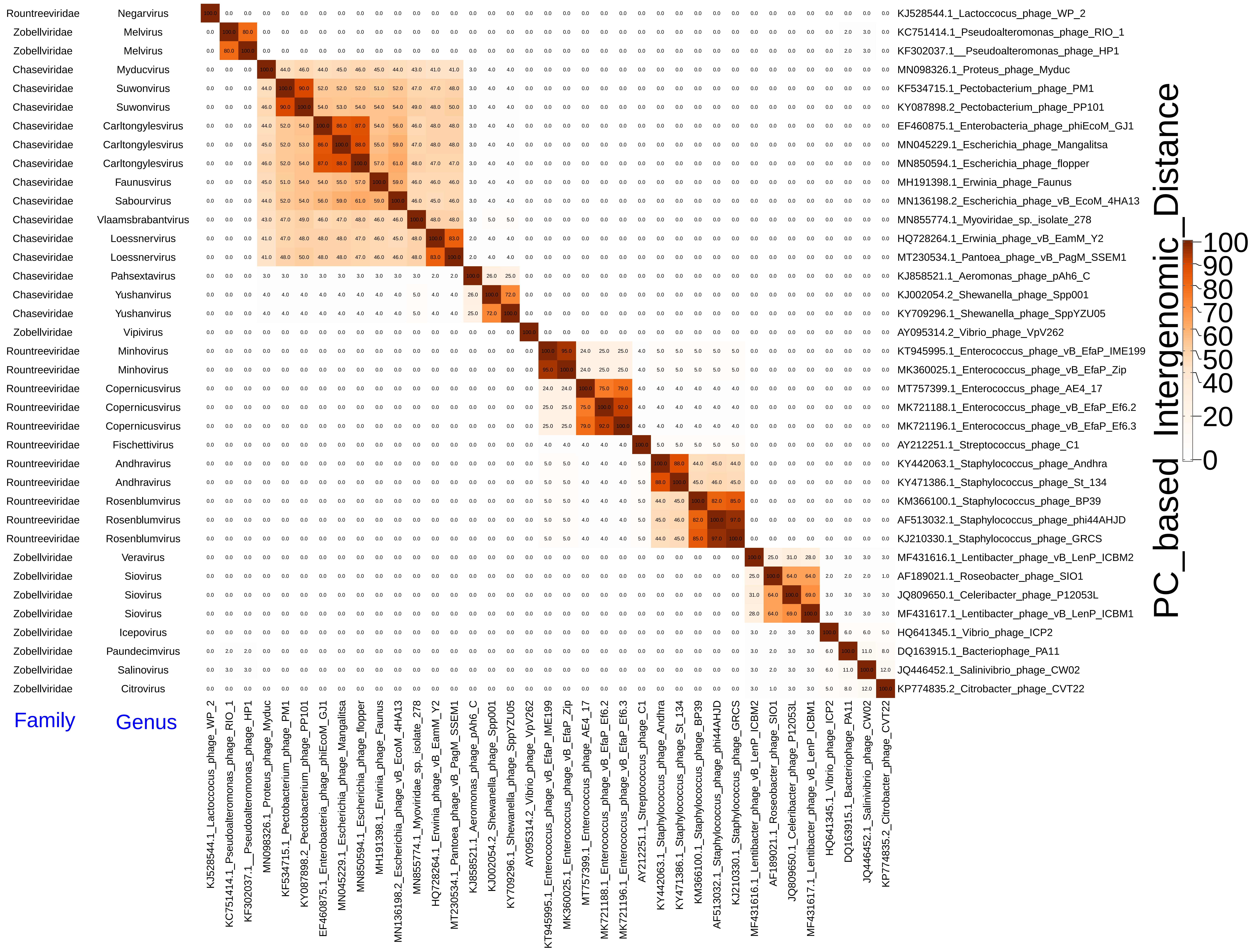

Fig. S2: Intergenomic distances for Crz_DB dataset, calculated with the following protein clustering parameters: coverage = 80%, % identity = 50%.

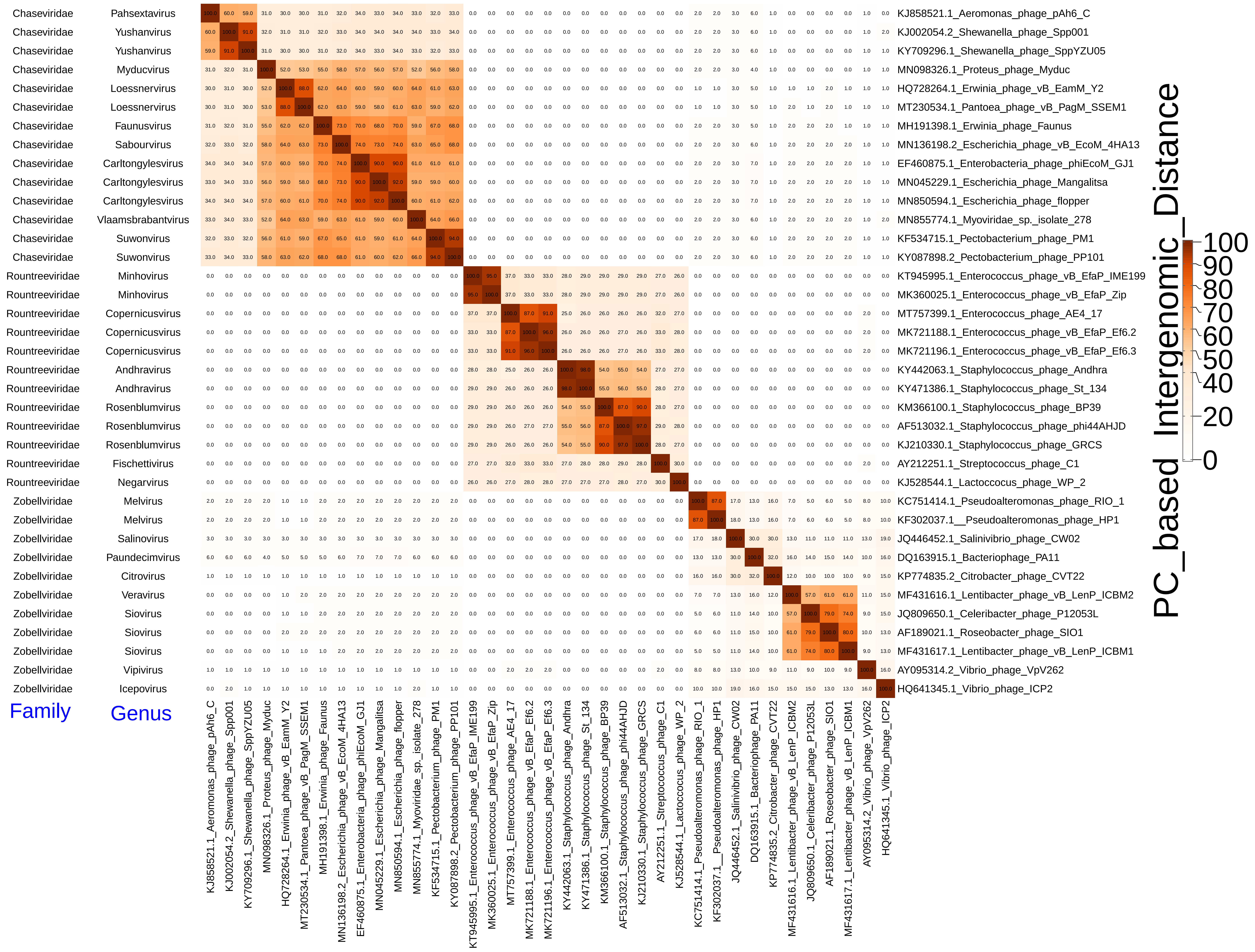

Fig. S3: Intergenomic distances for Crz_DB dataset, calculated with the following protein clustering parameters: coverage = 0%, % identity = 0%.

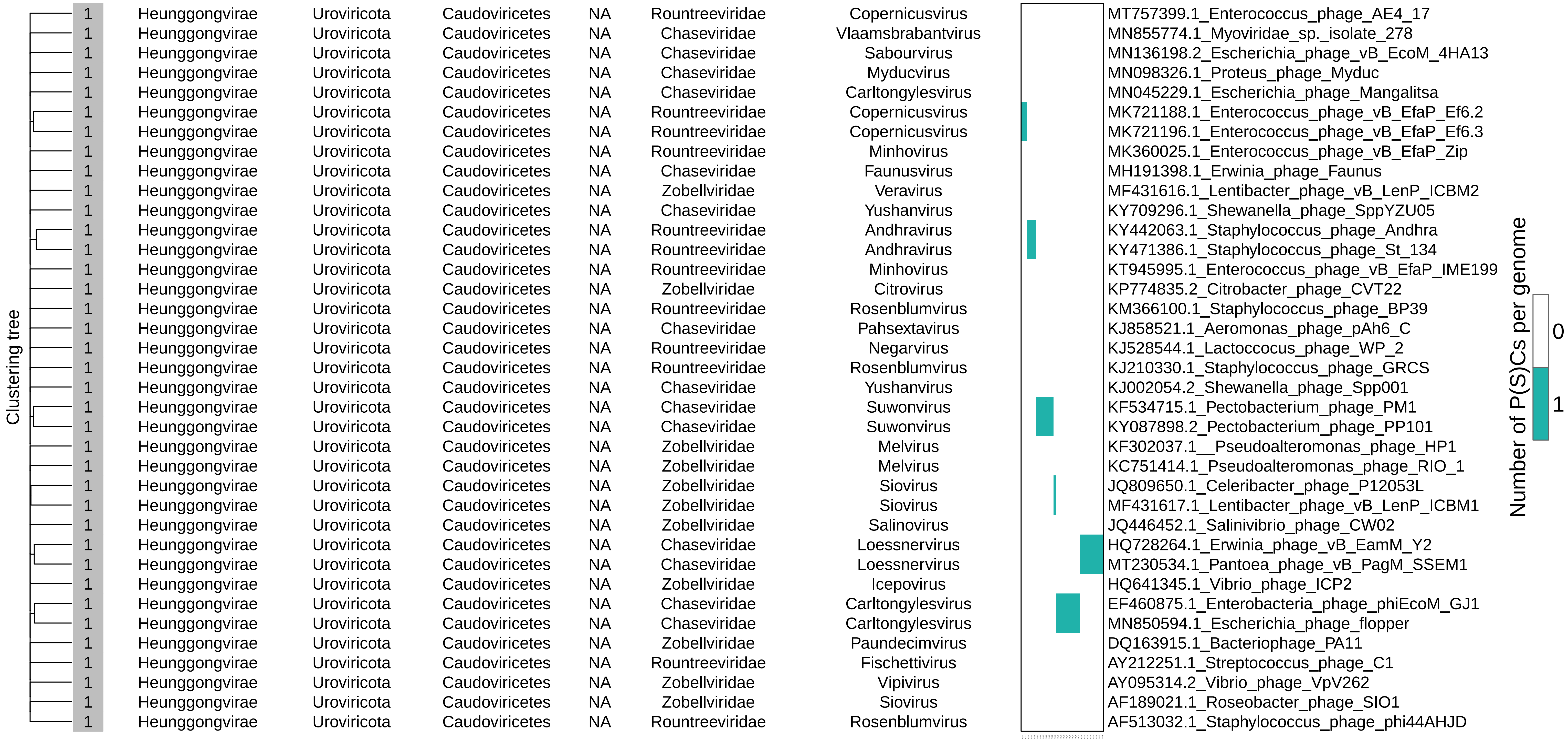

Fig. S4: Integrated visualization of the genome clustering of the Crz-DB dataset, calculated with the following protein clustering parameters: coverage = 100% and percent identity = 100%. Columns: 1) genome clustering tree; 2) Silhouette width; 3) VGC number; 4) Family; 5) Genus; 6) PC distribution; 7) genome names.

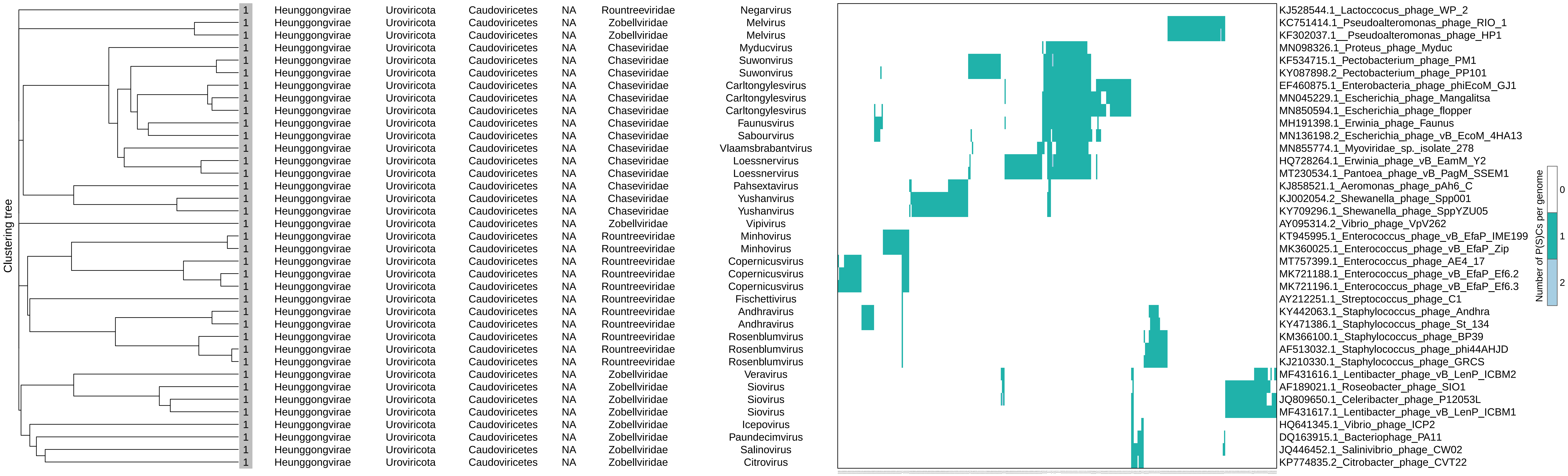

1	Heunggongvirae	Uroviricota	Caudoviricetes	NA	Rountreeviridae	Negarvirus	KJ528544.1_Lactoccocus_phage_WP_2
1	Heunggongvirae	Uroviricota	Caudoviricetes	NA	Zobellviridae	Melvirus	KC751414.1_Pseudoalteromonas_phage_RIO_1
1	Heunggongvirae	Uroviricota	Caudoviricetes	NA	Zobellviridae	Melvirus	KF302037.1__Pseudoalteromonas_phage_HP1
1	Heunggongvirae	Uroviricota	Caudoviricetes	NA	Chaseviridae	Myducvirus	MN098326.1_Proteus_phage_Myduc
1	Heunggongvirae	Uroviricota	Caudoviricetes	NA	Chaseviridae	Suwonvirus	KF534715.1_Pectobacterium_phage_PM1
1	Heunggongvirae	Uroviricota	Caudoviricetes	NA	Chaseviridae	Suwonvirus	KY087898.2_Pectobacterium_phage_PP101
1	Heunggongvirae	Uroviricota	Caudoviricetes	NA	Chaseviridae	Carltongylesvirus	EF460875.1_Enterobacteria_phage_phiEcoM_GJ1
1	Heunggongvirae	Uroviricota	Caudoviricetes	NA	Chaseviridae	Carltongylesvirus	MN045229.1_Escherichia_phage_Mangalitsa
1	Heunggongvirae	Uroviricota	Caudoviricetes	NA	Chaseviridae	Carltongylesvirus	MN850594.1_Escherichia_phage_flopper
1	Heunggongvirae	Uroviricota	Caudoviricetes	NA	Chaseviridae	Faunusvirus	MH191398.1_Erwinia_phage_Faunus
1	Heunggongvirae	Uroviricota	Caudoviricetes	NA	Chaseviridae	Sabourvirus	MN136198.2_Escherichia_phage_vB_EcoM_4HA13
1	Heunggongvirae	Uroviricota	Caudoviricetes	NA	Chaseviridae	Vlaamsbrabantvirus	MN855774.1_Myoviridae_sp._isolate_278
1	Heunggongvirae	Uroviricota	Caudoviricetes	NA	Chaseviridae	Loessnervirus	HQ728264.1_Erwinia_phage_vB_EamM_Y2
1	Heunggongvirae	Uroviricota	Caudoviricetes	NA	Chaseviridae	Loessnervirus	MT230534.1_Pantoea_phage_vB_PagM_SSEM1
1	Heunggongvirae	Uroviricota	Caudoviricetes	NA	Chaseviridae	Pahsextavirus	KJ858521.1_Aeromonas_phage_pAh6_C
1	Heunggongvirae	Uroviricota	Caudoviricetes	NA	Chaseviridae	Yushanvirus	KJ002054.2_Shewanella_phage_Spp001
1	Heunggongvirae	Uroviricota	Caudoviricetes	NA	Chaseviridae	Yushanvirus	KY709296.1_Shewanella_phage_SppYZU05
1	Heunggongvirae	Uroviricota	Caudoviricetes	NA	Zobellviridae	Vipivirus	AY095314.2_Vibrio_phage_VpV262
1	Heunggongvirae	Uroviricota	Caudoviricetes	NA	Rountreeviridae	Minhovirus	KT945995.1_Enterococcus_phage_vB_EfaP_IME199
1	Heunggongvirae	Uroviricota	Caudoviricetes	NA	Rountreeviridae	Minhovirus	MK360025.1_Enterococcus_phage_vB_EfaP_Zip
1	Heunggongvirae	Uroviricota	Caudoviricetes	NA	Rountreeviridae	Copernicusvirus	MT757399.1_Enterococcus_phage_AE4_17
1	Heunggongvirae	Uroviricota	Caudoviricetes	NA	Rountreeviridae	Copernicusvirus	MK721188.1_Enterococcus_phage_vB_EfaP_Ef6.2
1	Heunggongvirae	Uroviricota	Caudoviricetes	NA	Rountreeviridae	Copernicusvirus	MK721196.1_Enterococcus_phage_vB_EfaP_Ef6.3
1	Heunggongvirae	Uroviricota	Caudoviricetes	NA	Rountreeviridae	Fischettivirus	AY212251.1_Streptococcus_phage_C1
1	Heunggongvirae	Uroviricota	Caudoviricetes	NA	Rountreeviridae	Andhravirus	KY442063.1_Staphylococcus_phage_Andhra
1	Heunggongvirae	Uroviricota	Caudoviricetes	NA	Rountreeviridae	Andhravirus	KY471386.1_Staphylococcus_phage_St_134
1	Heunggongvirae	Uroviricota	Caudoviricetes	NA	Rountreeviridae	Rosenblumvirus	KM366100.1_Staphylococcus_phage_BP39
1	Heunggongvirae	Uroviricota	Caudoviricetes	NA	Rountreeviridae	Rosenblumvirus	AF513032.1_Staphylococcus_phage_phi44AHJD
1	Heunggongvirae	Uroviricota	Caudoviricetes	NA	Rountreeviridae	Rosenblumvirus	KJ210330.1_Staphylococcus_phage_GRCS
1	Heunggongvirae	Uroviricota	Caudoviricetes	NA	Zobellviridae	Veravirus	MF431616.1_Lentibacter_phage_vB_LenP_ICBM2
1	Heunggongvirae	Uroviricota	Caudoviricetes	NA	Zobellviridae	Siovirus	AF189021.1_Roseobacter_phage_SIO1
1	Heunggongvirae	Uroviricota	Caudoviricetes	NA	Zobellviridae	Siovirus	JQ809650.1_Celeribacter_phage_P12053L
1	Heunggongvirae	Uroviricota	Caudoviricetes	NA	Zobellviridae	Siovirus	MF431617.1_Lentibacter_phage_vB_LenP_ICBM1
1	Heunggongvirae	Uroviricota	Caudoviricetes	NA	Zobellviridae	Icepovirus	HQ641345.1_Vibrio_phage_ICP2
1	Heunggongvirae	Uroviricota	Caudoviricetes	NA	Zobellviridae	Paundecimvirus	DQ163915.1_Bacteriophage_PA11
1	Heunggongvirae	Uroviricota	Caudoviricetes	NA	Zobellviridae	Salinovirus	JQ446452.1_Salinivibrio_phage_CW02
1	Heunggongvirae	Uroviricota	Caudoviricetes	NA	Zobellviridae	Citrovirus	KP774835.2_Citrobacter_phage_CVT22

Fig. S5: Integrated visualization of the genome clustering of the Crz-DB dataset, calculated with the following protein clustering parameters: coverage = 80% and percent identity = 50%. Columns: 1) genome clustering tree; 2) Silhouette width; 3) VGC number; 4) Family; 5) Genus; 6) PC distribution; 7) genome names.

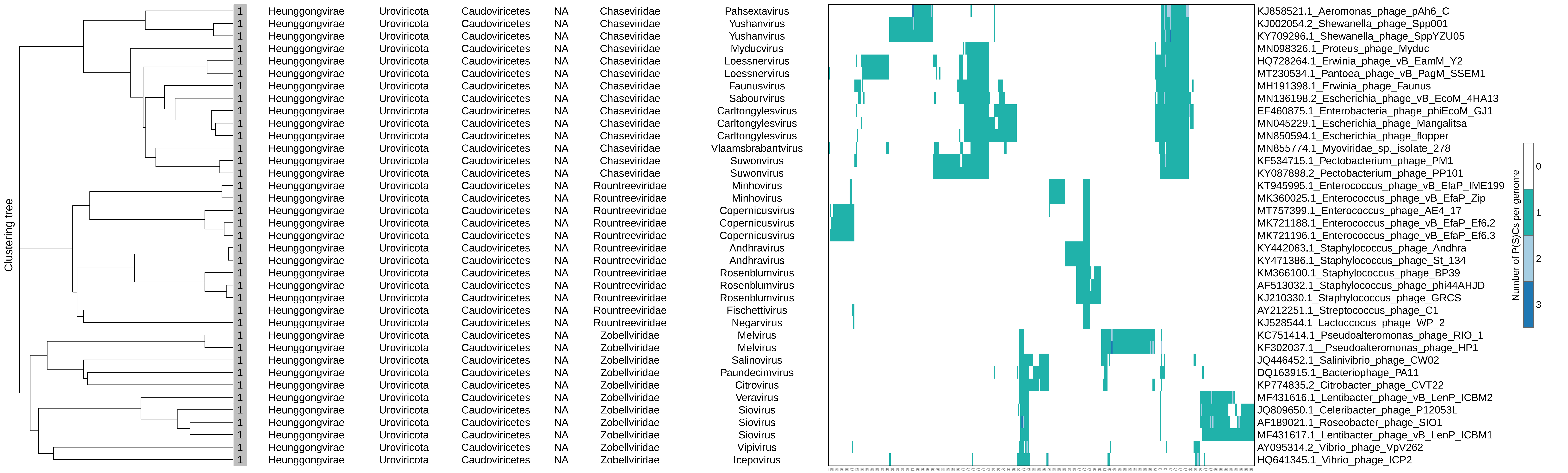

Fig. S6: Integrated visualization of the genome clustering of the Crz-DB dataset, calculated with the following protein clustering parameters: coverage = 0% and percent identity = 0%. Columns: 1) genome clustering tree; 2) Silhouett width; 3) VGC number; 4) Family; 5) Genus; 6) PC distribution; 7) genome names.
