# Supplementary material for: VirClust—A Tool for Hierarchical Clustering, Core Protein Detection and Annotation of (Prokaryotic) Viruses
Source: Viruses. 2023 Apr 19;15(4):1007. doi: 10.3390/v15041007 (PMC10143988; doi:10.3390/v15041007)
Supplement: Supplementary file 1 [file viruses-15-01007-s001.zip › SI_File9_Binder_alldist_Duplod_Varid.pdf]

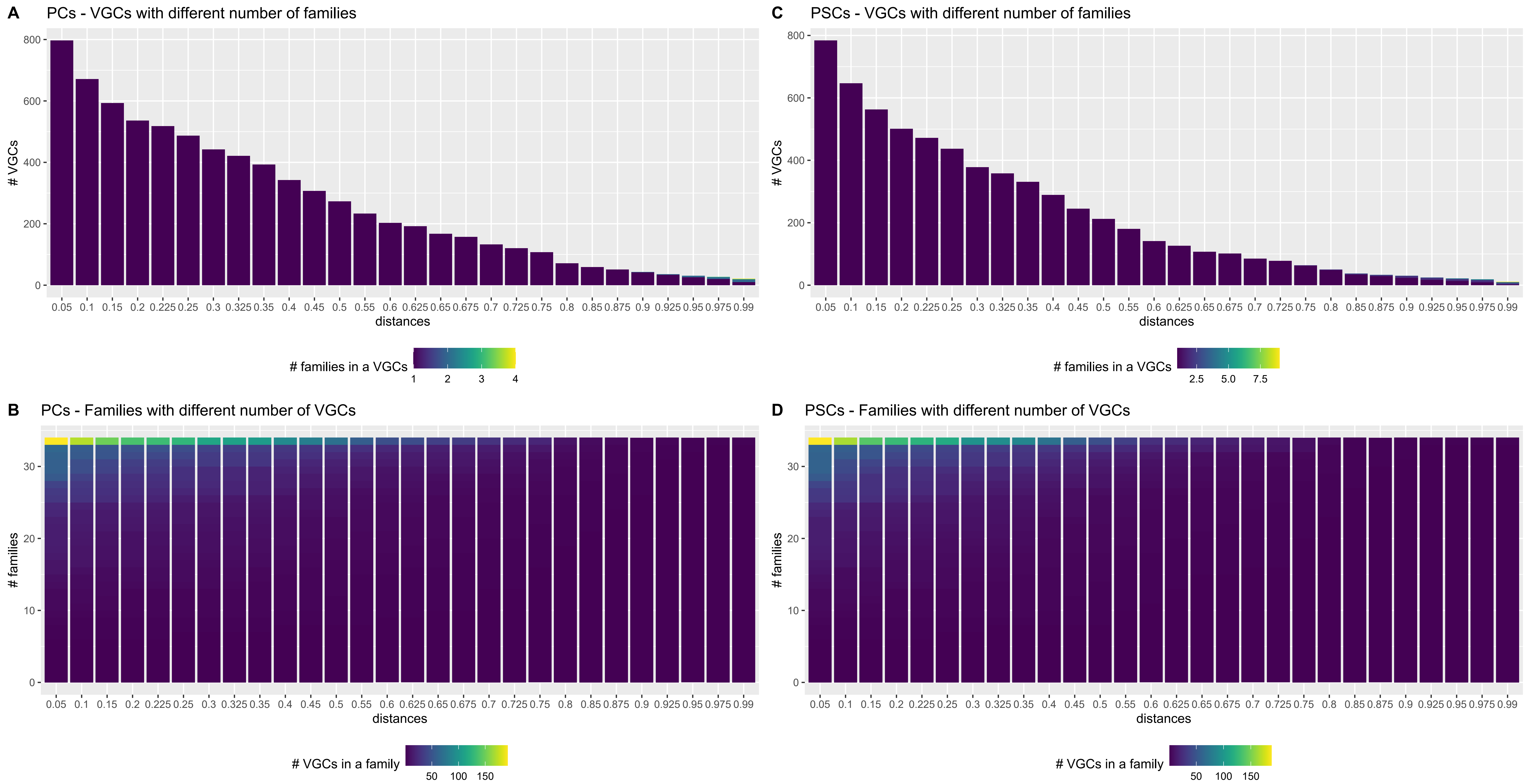

Fig. S1: Relationship between the number of VGCs and families for *Duplodnaviria*, when the PC- and PSC-based genomic trees of the Fam_DB dataset were cut with different intergenomic distances.

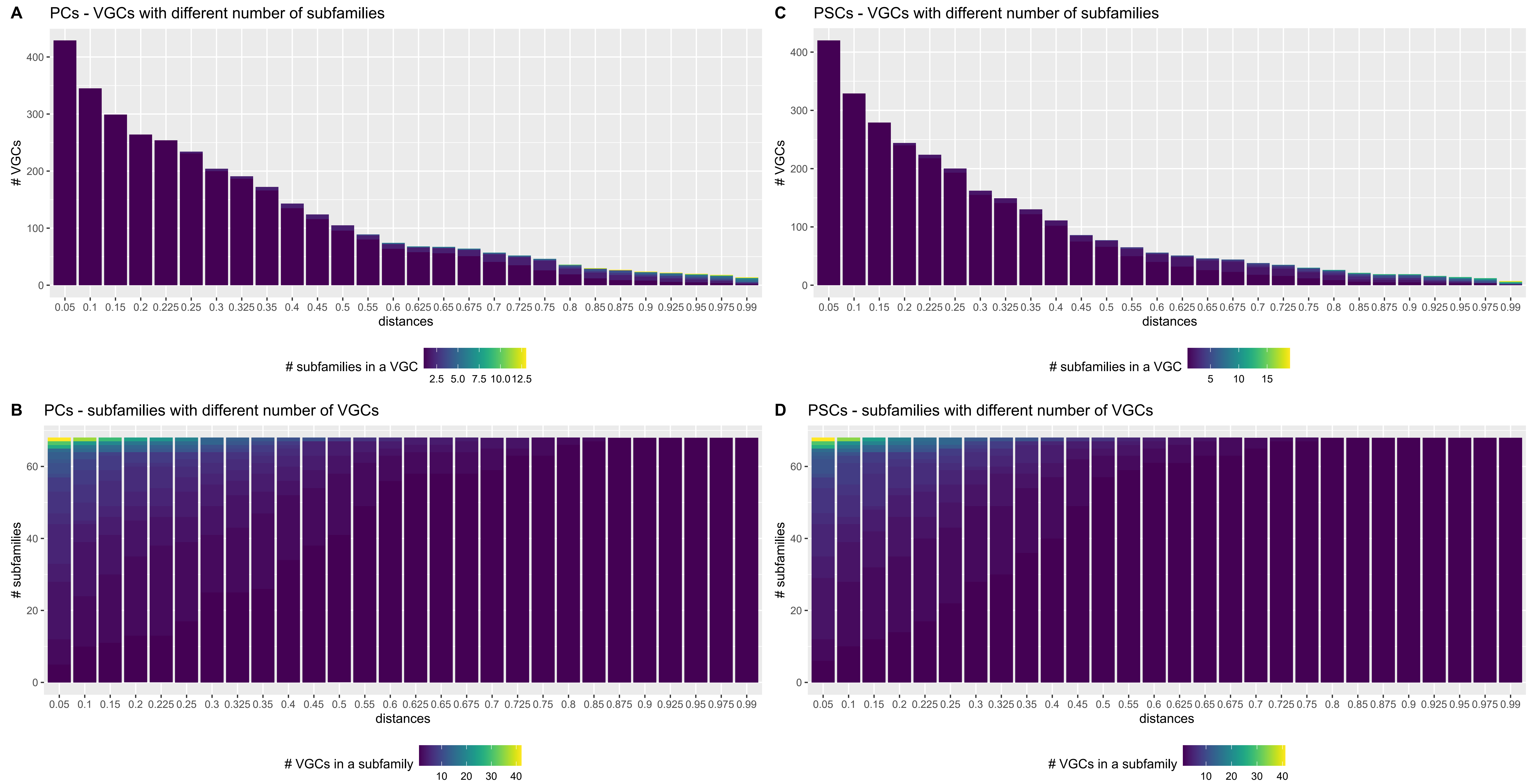

Fig. S2: Relationship between the number of VGCs and subfamilies for *Duplodnaviria,* when the PC- and PSC-based genomic trees of the Fam_DB dataset were cut with different intergenomic distances.

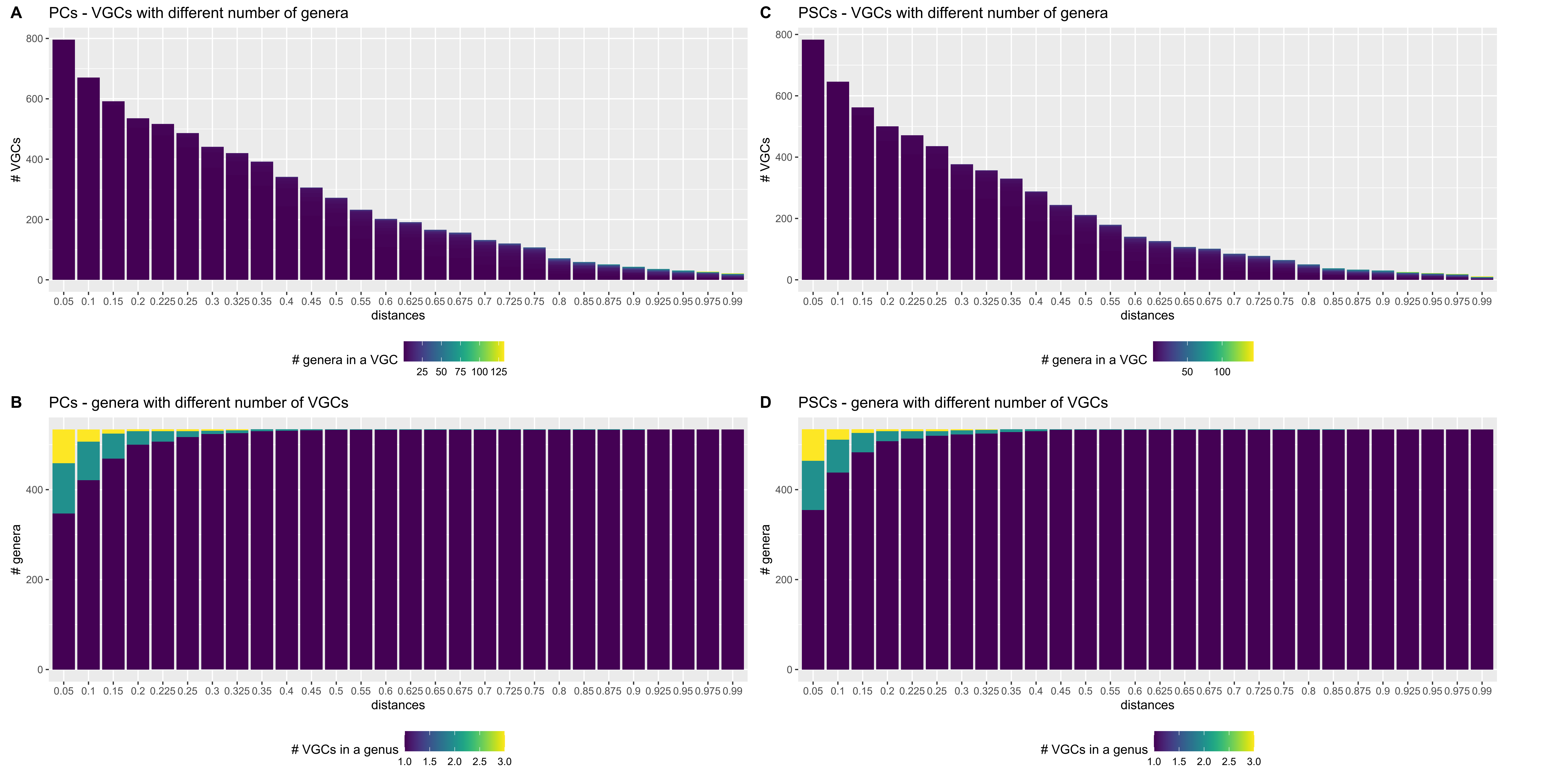

Fig. S3: Relationship between the number of VGCs and genera for *Duplodnaviria*, when the PC- and PSC-based genomic trees of the Fam_DB dataset were cut with different intergenomic distances.

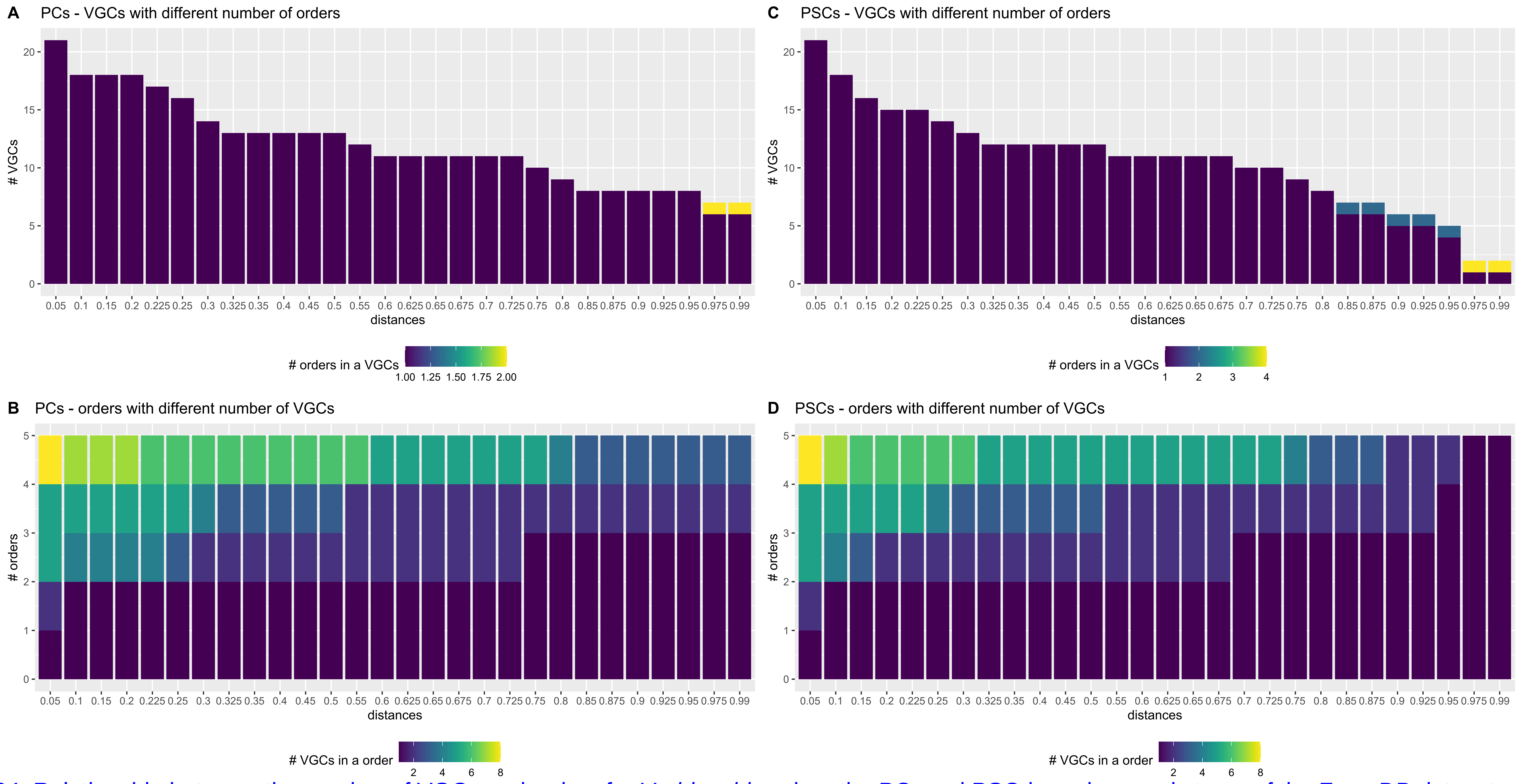

Fig. S4: Relationship between the number of VGCs and orders for *Varidnaviria*, when the PC- and PSC-based genomic trees of the Fam_DB dataset were cut with different intergenomic distances.

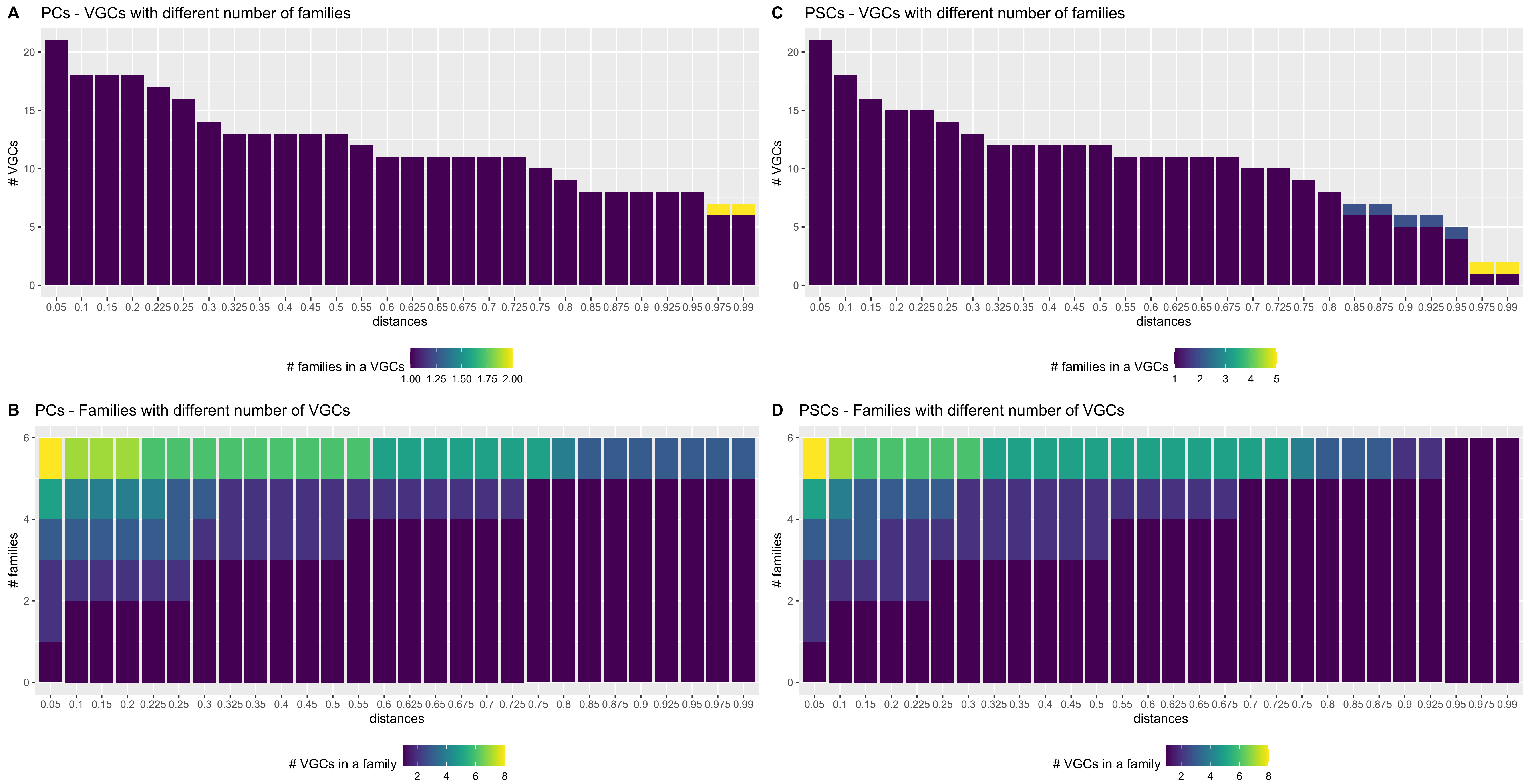

Fig. S5: Relationship between the number of VGCs and families for *Varidnaviria*, when the PC- and PSC-based genomic trees of the Fam_DB dataset were cut with different intergenomic distances.

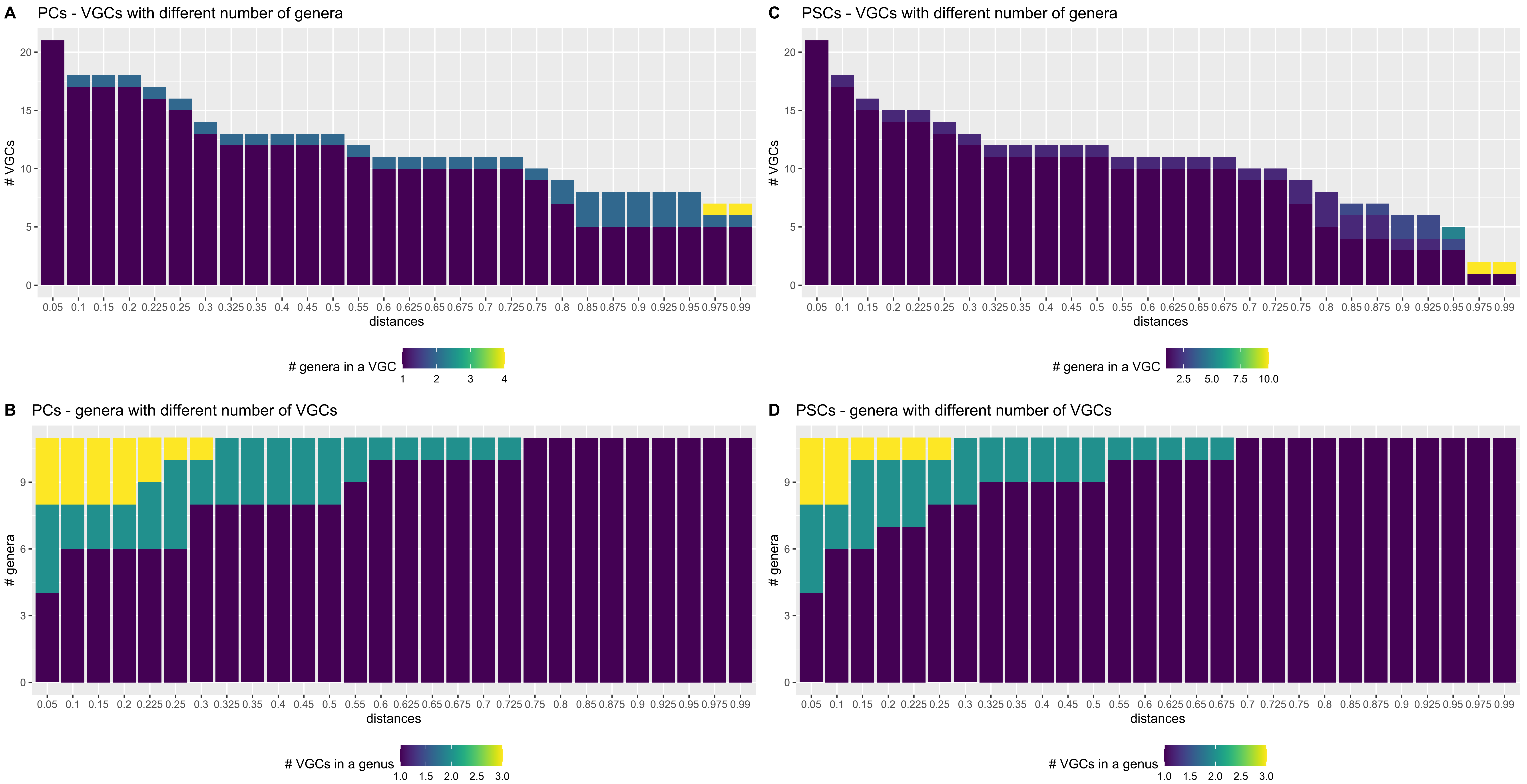

Fig. S6: Relationship between the number of VGCs and genera for *Varidnaviria*, when the PC- and PSC-based genomic trees of the Fam_DB dataset were cut with different intergenomic distances.
